# The Role of Microbiota and Fecal Transplantation in Inflammatory Bowel Disease

**DOI:** 10.3390/pathogens15040451

**Published:** 2026-04-21

**Authors:** Isabel Lagos, Edith Pérez de Arce, Ilaria Faggiani, Ferdinando D’Amico, Alessandra Zilli, Federica Furfaro, Sara Massironi, Clelia Cicerone, Virginia Solitano, Tommaso Lorenzo Parigi, Laurent Peyrin-Biroulet, Silvio Danese, Mariangela Allocca

**Affiliations:** 1 Department of Gastroenterology, Clinica Las Condes, Estoril 450, Santiago 8380453, Chile; ilagos@clinicalascondes.cl; 2 Department of Gastroenterology, Hospital Clinico Universidad de Chile, Dr. Carlos Lorca Tobar 999, Santiago 8380456, Chile; eperezdearce@gmail.com; 3 Department of Gastroenterology and Endoscopy, IRCCS San Raffaele Hospital, Via Oglettina 60, 20132 Milan, Italy; faggiani.ilaria@hsr.it (I.F.); damico.ferdinando@hsr.it (F.D.); zilli.alessandra@hsr.it (A.Z.); sara.massironi@libero.it (S.M.); cicerone.clelia@hsr.it (C.C.); tommaso.parigi@gmail.com (T.L.P.); danese.silvio@hsr.it (S.D.); 4 Department of Gastroenterology, Vita Salute San Raffaele University, Via Oglettina 60, 21132 Milan, Italy; 5 Department of Gastroenterology, INFINY Institute, INSERM NGERE, CHRU Nancy, F-54500 Vandoeuvre-lès-Nancy, France; peyrinbiroulet@gmail.com

**Keywords:** inflammatory bowel disease, Crohn’s disease, ulcerative colitis, microbiota, gastrointestinal microbiome, fecal microbiota transplantation, bile acids

## Abstract

Inflammatory bowel diseases (IBDs), including ulcerative colitis (UC) and Crohn’s disease (CD), are consistently associated with alterations in gut microbial communities, although the extent and characteristics of these alterations vary across studies, supporting a potential role of the microbiota in disease pathogenesis and therapeutic modulation. We conducted a systematic review to synthesize current evidence on microbiota alterations in IBD and the clinical application of fecal microbiota transplantation (FMT). A total of 118 studies were included (76 focused on microbiota profiling and 42 evaluated FMT as therapy). Across heterogeneous study designs and microbial characterization methods, reduced microbial diversity was the most consistently reported alteration, generally more pronounced in CD than in UC. Depletion of *Faecalibacterium prausnitzii*—a key butyrate producer with anti-inflammatory properties—was commonly reported, often accompanied by functional impairment in short-chain fatty acid production. Microbial patterns were frequently associated with mucosal inflammation and varied across disease phenotypes; these patterns have been increasingly explored as predictors of treatment response and relapse, although mechanistic interpretation remains limited and causal relationships are difficult to establish. Evidence from randomized controlled trials suggests potential efficacy of FMT in UC, particularly with intensive or repeated protocols, whereas data in CD remain limited and heterogeneous, with signals of benefit often appearing transient. FMT was generally well tolerated, but long-term safety data remain scarce. Emerging multi-omic approaches are reshaping the field by integrating taxonomic and functional insights, with potential implications for risk stratification, diagnosis, prognosis, and therapeutic optimization. Further standardized, longitudinal, and mechanistically oriented studies are required to translate microbiome research into clinically actionable strategies in IBD.

## 1. Introduction

Inflammatory bowel disease (IBD), encompassing Crohn’s disease (CD) and ulcerative colitis (UC), is a chronic relapsing condition with a multifactorial etiology, involving genetic susceptibility, immune dysregulation, environmental factors, and alterations in the intestinal microbiota [1]. Long before the era of high-throughput sequencing, clinical observations of bacterial overgrowth and ileal dysfunction in patients with CD suggested a possible role for microbial communities in disease pathogenesis [2]. Subsequent molecular analyses have demonstrated both compositional and functional alterations in the gut microbiome of IBD patients compared to healthy controls, with partially distinct features observed in CD and UC [3,4,5].

What emerges across cohorts is not a uniform microbial signature, but a recurring pattern of reduced diversity, depletion of protective commensals—*Faecalibacterium prausnitzii* being among the most consistently depleted—and expansion of bacteria with pro-inflammatory or pathogenic potential [4,6,7,8]. Beyond taxonomic shifts, functional changes in microbial metabolism, including altered short-chain fatty acid (SCFA) production, bile acid transformation, and interactions with the mucosal barrier, are closely linked to immune activation and intestinal inflammation [3,6,8,9]. Evidence from multiple studies suggests that microbiota contributes to disease pathogenesis, beyond serving as a secondary indicator of inflammation [5,10,11].

This perspective has stimulated interest in microbiota-targeted therapeutic strategies, particularly fecal microbiota transplantation (FMT), which has emerged as a direct approach to restoring microbial homeostasis in selected patients [12,13]. While FMT has demonstrated robust efficacy in recurrent *Clostridioides difficile* infection, its positioning in IBD remains under investigation [11,14,15]. Early clinical trials suggest potential benefits for inducing and maintaining remission in both UC and CD [16,17,18], although optimal protocols, donor selection, long-term effectiveness, and safety profiles remain incompletely defined [19,20,21].

In this context, the role of FMT in IBD remains unsettled [15,22]. This systematic review brings together current evidence on microbiota alterations in IBD and examines clinical data on FMT in both CD and UC. Mechanistic findings are discussed alongside clinical to provide perspective on the therapeutic role of microbiota-based interventions and the uncertainties that persist.

## 2. Materials and Methods

This systematic review was conducted in accordance with Preferred Reporting Items for Systematic Reviews and Meta-Analyses (PRISMA) 2020 guidelines [23]. A comprehensive literature search was performed in PubMed and the Cochrane Library from database inception through 31 December 2025, to identify relevant original articles. To enhance coverage, a complementary search on Google Scholar search was conducted to identify potentially relevant articles not captured by the primary database searches.

The search strategy combined Medical Subject Headings (MeSH) and keywords, including: “IBD”, “Inflammatory Bowel Diseases”, “Crohn’s Disease”, “colitis, ulcerative”, AND “microbiota”, “gastrointestinal microbiome”, “gut microbiota”, “gut microbiome”, “fecal microbiota transplantation”, and “bile acids”. All retrieved records were exported to SciSpace (Typeset.io) for management and deduplication. Duplicates were removed using the platform’s automated function, followed by manual verification to ensure accuracy and prevent erroneous exclusions. The full database-specific search strategies, complementary search procedure, search dates, and applied restrictions are provided in Supplementary Materials.

### 2.1. Eligibility Criteria

Eligible studies comprised those involving human subjects aged 18 years or older diagnosed with IBD, including clinical trials and observational studies that explored microbiome composition, metabolites, microbiota-targeted therapeutic interventions, and fecal microbiota transplantation. Exclusion criteria involved studies that did not focus primarily on IBD and the gut microbiome, as well as investigations of non-intestinal microbiomes (e.g., blood, skin, or urogenital microbiota). Additionally, studies not published in English, review papers, conference abstracts, case reports, editorials, and those with insufficient data for analysis were excluded.

### 2.2. Study Selection and Data Extraction

To ensure methodological rigor and minimize selection bias, two authors (I.L. and E.P.A.) independently reviewed study eligibility using predefined inclusion and exclusion criteria. Full-text articles were subsequently assessed for eligibility. Data extraction was also performed independently, with discrepancies resolved by consensus.

### 2.3. Quality Assessment

Methodological quality was assessed independently by two reviewers. Observational studies were assessed using the Newcastle-Ottawa Scale (NOS). Studies were categorized as low risk, some concerns, or high risk of bias (for RCTs) and as low, moderate, or high methodological quality (for observational studies). Disagreements were resolved by consensus. Results of Risk of Bias and Quality Assessment are provided in Supplementary Table S2.

Given the heterogeneity in study design, microbiome methodologies, and outcome reporting, quantitative meta-analysis was not performed.

### 2.4. Use of Artificial Intelligence Tools

Generative AI tools (Claude by Anthropic, Sonnet 4.6) were used to assist with language editing and manuscript polishing during the preparation of this review. These tools were not used for data extraction, quality assessment, or any analytical processes. All content, interpretations, and conclusions remain the sole responsibility of the authors.

## 3. Results

### 3.1. Search Results and Study Selection

A total of 632 records were identified through database search (PubMed, Cochrane, Google Scholar). After removing 317 duplicates, 315 unique records were screened for title and abstract. Of these, 152 records were excluded because of inappropriate study design, lack of a primary focus on microbiota analysis, non-IBD populations, or ineligible publication-type exclusions (review articles, editorials, conference abstracts, case reports). A PRISMA 2020 flow diagram is provided in Supplementary Table S1.

A total of 163 full-text articles were assessed for eligibility. Of these, 45 full-text articles were excluded due to insufficient microbiome data (*n* = 18), absence of extractable clinical outcomes (*n* = 13), case reports (*n* = 8), or non-English language (*n* = 6). Ultimately, 118 studies were included in the qualitative synthesis, including 76 evaluating microbiota alterations in IBD and 42 assessing FMT. Full-text articles excluded after eligibility assessment are detailed in Table S3.

**Figure 1:**
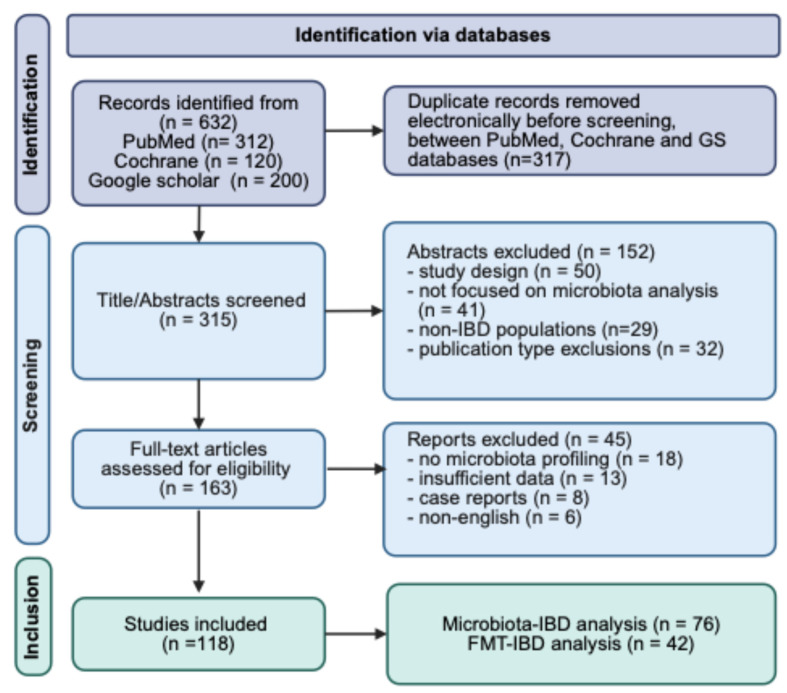
PRISMA flow diagram of study identification, screening, and inclusion.

The details of the article selection process are summarized in Figure 1.

Methodological quality was assessed for all 118 included studies. Among the 16 randomized controlled trials evaluated for FMT, risk of bias (RoB 2) was rated as low in 2 studies (12.5%), as raising some concerns in 14 studies (87.5%), and high risk in none. The main domains flagging concern were blinding challenges and missing outcome data. Among the 76 observational microbiota studies assessed by the Newcastle-Ottawa Scale (NOS), 5 studies (6.6%) were rated as good quality (NOS ≥ 7), 38 (50.0%) as fair quality (NOS 4–6), and 33 (43.4%) as poor quality (NOS ≤ 3). The most common limitations were insufficient comparability adjustment and small sample sizes. Full quality assessment details are provided in Supplementary Table S2.

### 3.2. Microbiota Alterations in Inflammatory Bowel Disease

**Figure 2:**
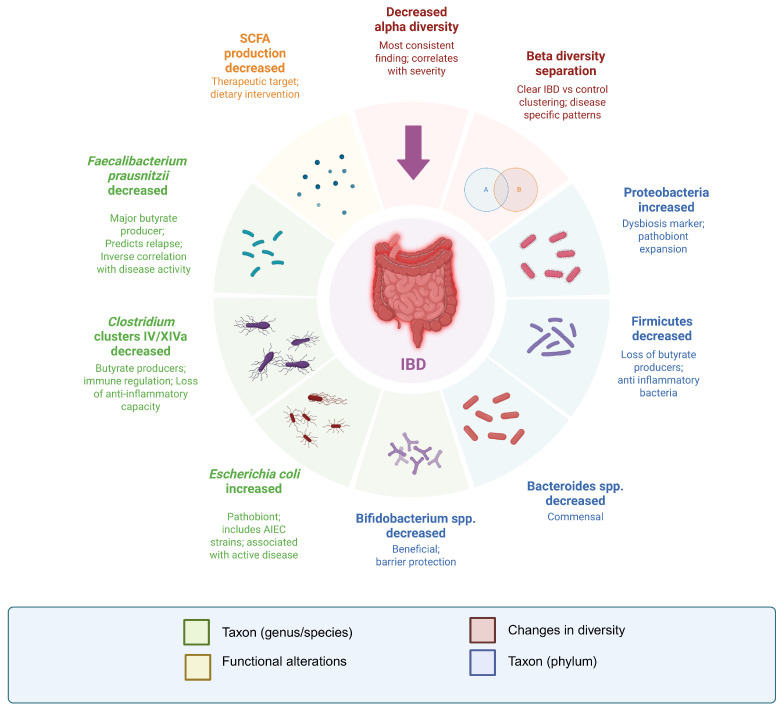
Microbiota alterations in IBD.

The intestinal microbiota has been extensively investigated as a central component of IBD pathogenesis. Over three decades of investigation, a consistent pattern emerges: IBD is not defined as a single microbial signature, but by reproducible disturbances involving reduced microbial diversity, depletion of beneficial commensals, expansion of pathobionts, and functional impairment. These recurring alterations are summarized in Figure 2. The following section synthesizes shared and phenotype-specific alterations identified among the 76 included studies.

The 76 included studies spanned three decades (1995–2025), with most published between 2020 and 2024. Among the 76 microbiota studies, cross-sectional designs were most frequent (*n* = 18), followed by prospective cohorts (*n* = 16), case–control studies (*n* = 11), and randomized controlled trials (RCTs) (*n* = 12); 19 studies used other designs. Microbiome profiling methods varied substantially: 16S rRNA sequencing was the most common approach (43/76, 56.6%), followed by shotgun metagenomics (22/76, 28.9%). Culture-based approaches were uncommon (3/76, 3.9%, predominantly in earlier publications). Other methods included targeted qPCR (3/76, 3.9%), FISH (2/76, 2.6%), microarray-based profiling (2/76, 2.6%), and T-RFLP (1/76, 1.3%). Overall, the evidence base is shaped by methodological heterogeneity and has recently shifted toward higher-resolution, multi-omics approaches. The characteristics of the included studies are provided in Supplementary Table S4.

#### 3.2.1. Microbial Diversity Changes

Reduced microbial diversity was the most consistently reported alteration, identified in nearly all included studies. Decreased alpha diversity—reflecting reduced species richness and evenness—was observed in both UC and CD compared with healthy controls. At the community level, beta diversity—capturing differences in overall microbial composition between groups—demonstrated clear separation between IBD and control samples in the vast majority of studies. Ordination analyses (e.g., PCoA plots based on Bray–Curtis or UniFrac distances) showed clustering of IBD samples distinct from healthy individuals, suggesting a structured disease-associated shift in microbial community composition rather than random inter-individual variation.

Collectively, these findings indicate that loss of microbial diversity is a hallmark feature of IBD-associated dysbiosis.

#### 3.2.2. Shared Taxonomic Shifts in UC and CD

At the phylum level, depletion of Firmicutes was reported in 57 of 76 studies (75%), whereas expansion of Proteobacteria was observed in 48 of 76 (63.2%). This pattern reflects a compositional shift from obligate anaerobic, short-chain fatty acid (SCFA)-associated communities toward facultative anaerobes that are more competitive under inflammatory and oxidative stress conditions.

At the species level, the most consistently reported commensal loss was *Faecalibacterium prausnitzii* (*F. prausnitzii*), identified in 69 of 76 studies (90.8%), and frequently described as inversely associated with disease activity or poorer outcomes. Conversely, *Escherichia coli* enrichment was reported in 52 of 76 studies (68.4%), noting a predominance in CD, particularly in patients with ileal involvement. Additionally, several studies specifically identified Adherent-Invasive *Escherichia coli* (AIEC) strains in CD cohorts [24].

#### 3.2.3. Differential Microbiota Patterns in UC and CD

Although UC and CD shared core dysbiotic features—including reduced microbial diversity, depletion of SCFA-producing Firmicutes, and enrichment of facultative organisms—the included studies support phenotype-specific differences in pattern and consistency (Table 1) [7,25]. Overall, dysbiosis appeared more distinct in CD, particularly in patients with ileal involvement [26,27,28]. Recurrent signals in CD included greater depletion of *F. prausnitzii* and *Roseburia*, and enrichment of Enterobacteriaceae- and *Escherichia*/*Shigella*-related taxa. In some CD cohorts, *Ruminococcus gnavus* (*R. gnavus*) was also enriched, especially in ileal disease [8,26,27,28].

In contrast, UC showed a similar but less uniform pattern, more closely linked to severity than to a single disease-specific pathobiont. Collectively, the evidence suggests that while UC and CD share a common dysbiotic framework, CD–particularly ileal disease–is more often characterized by deeper loss of butyrate-associated commensal and enrichment of facultative, inflammation-adapted organisms [8,26,27,28,29].

#### 3.2.4. Microbial Signatures Associated with Disease Activity, Phenotype, and Complications in IBD

Rather than supporting a single pathogen-driven signature, the included studies indicate that microbiota alterations in IBD are context-dependent and more closely related to the degree of mucosal inflammation, disease location, and clinical settings than to any single organism [30,31]. Higher disease activity often showed a more dysbiotic configuration, but this was not uniform across all cohorts, and was more consistently captured by biologic or endoscopic activity rather than symptom-based scores [8,32,33,34,35]. In ulcerative colitis, active disease and acute severe colitis (ASUC) were associated with lower diversity and greater loss of commensal Clostridial taxa than in controls or in milder disease [27,28]. In Crohn’s disease, patients with both active and quiescent disease exhibited lower diversity compared to healthy siblings. Active disease was associated with a wider range of inflammation-related taxa, including increased levels of Escherichia, Shigella, and Enterobacteriaceae-related signals, which correlated with serum inflammatory markers [8]. Importantly, not all CD cohorts showed activity-related differences in microbiota composition; in some studies, dysbiosis was observed regardless of disease activity, indicating that factors beyond disease activity influence microbiota composition [26].

A recurring observation across active colitis, quiescent CD, and adverse-outcome cohorts was the depletion of SCFA-associated taxa, especially *F. prausnitzii* and other members of the *Lachnospiraceae* and *Ruminococcaceae*. In active IBD, Sokol et al. reported lower levels of Firmicutes, including *F. prausnitzii*, in active CD and UC compared to healthy subjects, with <109 cells/g predicting postoperative recurrence in CD (HR = 1.8, *p* = 0.03) [7]. Nishikawa et al. observed a loss of multiple clostridial species in UC mucosa [27]. In ASUC, Kedia et al. further demonstrated a progressive decline in health-associated Clostridia, such as *Roseburia* and *Faecalibacterium*, with increasing disease severity [28]. In quiescent CD, Chen et al. identified reduced levels of *Faecalibacterium*, *Dorea*, and *Fusicatenibacter*, despite clinical remission, with these taxa correlating to lower SCFA concentrations (r = 0.62, *p* < 0.001), indicating ongoing metabolic dysfunction even during remission [8]. In the STORI cohort, low *F. prausnitzii* predicted relapse after infliximab withdrawal, independently of CRP [36]. Overall, these findings demonstrate disruption of butyrate-producing bacteria, which are likely to exert anti-inflammatory effects.

Phenotype-specific and complication-specific signatures were also described. Ileal disease was repeatedly distinguished from colonic CD by stronger depletion of *Faecalibacterium* and *Roseburia* and greater enrichment of Enterobacteriaceae-related taxa [26,27,28]. Postoperative CD patients who later recurred showed reduced alpha diversity and depletion of *Lachnospiraceae*/*Ruminococcaceae*-associated taxa, including *Anaerostipes* and *Faecalibacterium*, as well as enrichment of *Gammaproteobacteria*-associated organisms such as *Klebsiella*, *Escherichia-Shigella*, and *Enterococcus* [34,37,38]. These data support a shift toward inflammation-adapted organisms across different clinical contexts, with specific taxonomic variations associated with the clinical setting rather than a single IBD signature [39].

The protective role of *F. prausnitzii* extends beyond simple abundance metrics: multiple studies documented inverse correlations between *F. prausnitzii* levels and fecal calprotectin, CRP, and pro-inflammatory cytokines, including TNF-α and IL-6 [7,9,39]. *F. prausnitzii* depletion was consistently observed in IBD patients [20,24], with mechanistic studies showing NF-κB pathway inhibition [40] and butyrate-mediated anti-inflammatory effects. Other butyrate-producing taxa, including *Roseburia* spp., showed similar depletion patterns in active IBD and inverse correlations with calprotectin and CRP [40,41]. Bacteroides fragilis, which promotes regulatory T cell (Treg) differentiation, was also depleted in active UC [20]. *Bifidobacterium* spp., which produce acetate and lactate and have documented anti-inflammatory properties, showed variable patterns across different disease contexts [26,41,42].

When functional assessments were available, the results were directionally consistent with impaired SCFA-related metabolism, but not sufficiently uniform to justify pooling percentages or fixed-effect sizes. Chen et al. linked the quiescent CD signature to lower fecal SCFAs and showed enrichment of oxygen-dependent metabolic pathways in active CD [8]. Further supporting data come from Borren et al., who described depletion of the butyrate synthesis pathway in fatigued quiescent IBD [6]. Metagenomic analysis revealed downregulation of butyrate synthesis pathways (butyryl-CoA: acetate CoA-transferase and butyrate kinase) and an expansion of pathways associated with oxidative stress response and antibiotic resistance. These metabolic shifts may have functional consequences: butyrate is the primary energy source for colonocytes, regulates intestinal barrier function, and promotes regulatory T cell differentiation. Consequently, butyrate depletion may perpetuate inflammation [43,44].

These compositional changes contribute to interconnected pathways through which dysbiosis promotes mucosal inflammation. An increase in Gram-negative Enterobacteriaceae [20,30,33,40,43] raises luminal lipopolysaccharide (LPS) levels; when the barrier is compromised, translocated LPS binds Toll-like receptor 4 (TLR4) on immune and epithelial cells, thereby activating NF-κB and producing TNF-α, IL-6, IL-8, and IL-1β [20,38]. Dysbiosis-related inflammation also disrupts tight junction proteins (zonula occludens-1, occludin, claudins), increases intestinal permeability, and facilitates microbial antigen translocation, thereby forming a self-amplifying cycle in which barrier breakdown triggers immune activation, which further damages epithelial integrity [4,30,38]. Higher disease activity was also linked to a greater abundance of pathobionts, especially *Enterobacteriaceae* (including *E. coli*) observed in numerous studies in both UC and CD patients [8,26,27,35,40,45]. In CD, *E. coli* enrichment is particularly pronounced in ileal disease [26,27,35]. Adherent-invasive *E. coli* (AIEC) strains have been identified in multiple studies, demonstrating enhanced capacity to adhere to the intestinal epithelium, while their abundance has been associated with elevated pro-inflammatory cytokine levels and with granuloma formation [46,47]. *E. coli* from CD biopsies demonstrates enhanced pathogenic properties, including survival within macrophages and increased invasion capabilities [26,35]. In UC, *E. coli* enrichment is associated with elevated inflammatory markers, including fCP and CRP elevation [8,20,40], with the most pronounced increases observed in patients with extensive colitis. Likewise, *Fusobacterium* spp. and *Enterococcus* spp. expanded during active disease [20,34,35].

*R. gnavus* shows variable patterns across IBD subtypes, with enrichment reported in some CD cohorts [34,42], but decreased abundance in other IBD populations [40]. Its mucin-degrading ability may compromise the protective mucus layer [48]. Notably, *R. gnavus* produces inflammatory polysaccharides that may stimulate pro-inflammatory cytokine production, potentially contributing to damage of the protective mucus layer [33].

#### 3.2.5. Preclinical Microbiota Signatures

While most disease activity-associated microbiota signatures reflect clinically manifest inflammation, emerging evidence indicates that microbiota alterations may precede IBD onset. In a cross-sectional study, healthy co-twins of IBD patients exhibit intermediate dysbiosis profiles between affected siblings and healthy controls, including reduced expression of *F. prausnitzii* [49]. In the long term, declining diversity precedes clinical relapse by 2–3 months [50], and asymptomatic first-degree relatives display subclinical dysbiosis [51]. These preclinical findings suggest early pathogenic changes that may serve as biomarkers for risk stratification in genetically susceptible individuals [34,37,38,39,52,53,54].

#### 3.2.6. Phenotype-Related Associations

Phenotype-related differences in dysbiosis were more clearly defined in CD than in UC. In UC, the included studies more consistently linked microbiota alterations to inflammatory severity than anatomical extent. Active UC showed reduced mucosa-associated diversity and loss of commensal clostridial taxa compared with controls or inactive disease, whereas ASUC exhibited greater microbial disruption than mild-to-moderate disease [32,33,55]. In CD, phenotype-specific patterns were more distinct. Ileal CD showed lower diversity than colonic CD and healthy controls, and was characterized by greater depletion of *F. prausnitzii* and *Roseburia*, with enrichment of *Enterobacteriaceae*/*E. coli* and, in some cohorts, *R. gnavus* [26,27,28,47]. Complementing these findings, small-intestinal luminal metagenomes (ileostomy/ileal pouch) showed reduced diversity, enrichment of oral and upper gastrointestinal taxa (e.g., *Streptococcus*, *Veillonella*, *Actinomyces*), and depletion of butyrate pathways, consistent with a distinct small-intestinal ecosystem [56]. Likewise, postoperative ileal and ileocolonic CD studies have associated reduced diversity with enrichment of Proteobacteria and Enterobacteriaceae, which increases the risk of endoscopic recurrence, whereas *Lachnospiraceae*-dominant communities were linked with a lower recurrence risk [35,36].

#### 3.2.7. Treatment Response and Relapse Prediction

Predicting therapeutic response and anticipating relapse remain major challenges in IBD, generating growing interest in the gut microbiota as a tool for risk stratification and outcome prediction [39,57,58]. Several included studies linked the baseline gut microbiota to treatment response, particularly in patients treated with anti-TNF agents [50,58,59]. Responders generally showed higher alpha diversity and relative preservation of beneficial commensals such as Faecalibacterium, whereas non-responders more often evidenced reduced diversity [57,60]. In CD, Rajca et al. reported that low baseline diversity (Shannon index < 2.5) was associated with infliximab failure, with 78% sensitivity and 7% specificity, suggesting a similar pattern in non-responders [50]. Overall, predictive signals were directionally consistent but heterogeneous through the analysed data set.

Beyond global diversity measures, several studies suggested that basal community composition adds a clinically relevant signal for response prediction. Responders more often presented a commensal-enriched profile—particularly higher *Faecalibacterium* and *Roseburia*—*whereas* non-responders tended to show lower diversity and enriched pathobiont configuration [58,59]. These observations support the use of microbiota profiling as a complementary tool for outcome prediction, although the specific features and thresholds remain to be defined [50,58,59].

Relapse prediction was also commonly evaluated, with approximately one-third of UC and CD reports assessing microbiota-based forecasting. Longitudinal data suggest that microbial deterioration can precede relapse by weeks, particularly reductions in diversity and in butyrate. Machiels et al. showed in UC that combined depletion of *F. prausnitzii* and *Roseburia hominis* during remission predicted 6-month relapse (AUC 0.79; 95% CI: 0.67–0.91), outperforming fecal calprotectin (fCP) alone (AUC 0.68) [39]. In CD, Rajca et al. analyzed patients from the STORI cohort after Infliximab discontinuation (33 patients; 19 relapsed) and found that a probiotic profile characterized by reduced *Firmicutes* (notably low *F. prausnitzii* and low Bacteroidetes predicted relapse independently of CRP and was associated with a shorter time to relapse [50]. A systematic summary of microbiota-based response and relapse predictors across included studies is provided in Supplementary Table S7.

#### 3.2.8. Biological Therapy and Microbiota Alterations

A subset of studies evaluated longitudinal changes in the microbiota during biologic therapy, most commonly anti-TNF agents, with limited evidence for gut-selective anti-integrin and anti-IL-12/23 pathways [57,60].

In responders, anti-TNF treatment was frequently associated with a “health-associated” profile, characterized by an increase in alpha diversity and enrichment of butyrate-associated commensals (*Faecalibacterium* and *Roseburia*), together with attenuation of Proteobacteria and Enterobacteria [60].

Doherty et al. studied baseline microbiota predictors of response in a prospective CD cohort treated with ustekinumab. High baseline Faecalibacterium (>3% relative abundance) and Roseburia (>2%) associated with 8-week response (OR 4.2, 95% CI: 1.8–9.7, *p* = 0.001) [57]. In contrast, high *Escherichia* (>5%) predicted non-response (OR 0.3, 95% CI: 0.1–0.7, *p* = 0.006) [57]. On the other hand, evidence on the effects of small-molecule agents on the microbiota (JAK inhibitors, S1P modulators) was limited in this systematic review, indicating inconsistent reporting or sparse coverage.

#### 3.2.9. Gut Virome

Viral microbiome (virome) profiling was nearly absent from the dataset (1/76 studies). This represents a critical knowledge gap, as bacteriophages are the most abundant entities in the intestine and regulate bacterial populations. Majzoub et al. reported a secondary analysis of existing FMT trials (FOCUS and LOTUS), demonstrating phageome dysbiosis in active UC and linking remission to an *Oscillospiraceae*-associated phage signature [61].

#### 3.2.10. Mycobiome

Mycobiome reporting was minimal, with only 1 study (1/76) performing combined bacterial 16S rRNA and fungal Internal Transcribed Spacer (ITS) sequencing. Liguori et al. analyzed mucosal samples from patients with CD in both flare and remission, stratified by anatomical location (inflammatory vs. non-inflammatory mucosa). The study identified alterations in Candida species as the primary dysbiosis profile among fungi [62]. Active CD exhibited 3-fold higher Candida Albicans abundance in mucosal active disease compared to remission (*p* = 0.02), together with reduced fungal diversity (Shannon 1.8 vs. 2.6, *p* = 0.04). It is important to highlight that fungal dysbiosis correlated with bacterial alterations, suggesting coordinated multi-kingdom adaptations. The scarcity of mycobiome data is a major limitation, as fungi account for ~0.1% of gut microbiota biomass but exert strong immunomodulatory effects [55,56].

#### 3.2.11. Metabolites and Pathogen-Host Interactions

When metabolomic or functional profiling was available, findings showed reduced SCFA-generating capacity (particularly butyrate), along with altered bile acid metabolism and disrupted amino acid pathways, consistent with an inflammation-linked dysbiosis profile [6,9,57]. These shifts appear biologically meaningful. Lower butyrate availability is commonly linked to reduced epithelial energy support and barrier integrity [29,30], while bile acid alterations may disrupt FXR/TGR5 signaling and downstream mucosal immune regulation [58,59]. Complementary evidence comes from in vitro fermentation, which shows reduced butyrate production in UC-derived communities compared with healthy inoculum [63]. Facchin et al. further support the modifiability of this functional axis: microencapsulated butyrate supplementation induced measurable changes in microbiota composition and quality of life in IBD [64]. Shotgun metagenomics also linked symptom phenotypes to functional capacity in quiescent IBD: fatigue was associated with depletion of key butyrate producers (*F. prausnitzii*, *Roseburia hominis*), reduced butyrate-pathway abundance, enrichment of *R. gnavus*, and circulating metabolites (tryptophan-related), suggesting a potential link between microbial functional capacity and extraintestinal symptom burden [6]. Multi-omics and metabolomic data across included studies are summarized in Supplementary Table S8.

### 3.3. Fecal Microbiota Transplantation and Pathogen Modulation

Fecal microbiota transplantation has been investigated as a therapeutic strategy to directly modulate the intestinal microbial ecosystem in IBD [13,61,62]. Its proven efficacy in recurrent *Clostridioides difficile* infection established a foundational concept that extensive microbial replacement can lead to significant clinical improvements, encouraging investigation into its potential in IBD [64,65]. However, unlike in *C. difficile* infection, dysbiosis is only one of the etiological factors in the pathogenesis. IBD often presents a more complex therapeutic scenario, characterized by immune dysregulation, persistent mucosal inflammation, and associated complications [1,29,39]. Against this background, the clinical application of FMT in IBD has raised important questions regarding its optimal delivery, efficacy, safety, and role within existing therapeutic strategies [22,63,66,67,68].

The present systematic review evaluated the clinical efficacy, durability, and mechanistic correlates of FMT across IBD phenotypes. The evidence base was dominated by UC, with substantially fewer CD studies and a small number of mixed UC/CD cohorts in recurrent *Clostridioides difficile* infection. Study designs were heterogeneous, including randomized and non-randomized studies. Because some reports represented maintenance extensions or subgroup analyses, a unique pooled patient count was not derived. Key FMT study characteristics and outcomes are summarized in Table 2 and detailed in Supplementary Table S9.

#### 3.3.1. Efficacy of FMT in Ulcerative Colitis

RCTs provide the most robust evidence supporting the efficacy of FMT in UC, although remission rates varied considerably across studies. An early placebo-controlled trial by Moayyedi et al., using weekly retention enemas for six weeks, induced clinical and endoscopic remission in 9 of 38 patients (24%) receiving FMT compared with 2 of 37 (5%) in the placebo arm (95% CI 2–33; *p* = 0.03) with no significant difference in serious adverse events (SAEs) between groups [66]. In contrast, the multicenter double-blind RCT by Rossen et al., which delivered FMT via duodenal infusion through gastroscopy, did not demonstrate a significant difference over placebo, with clinical remission at week 12 observed in 30% of FMT-treated patients versus 20% in the autologous stool group (*p* = 0.051) [67]. Building on these earlier findings, Paramsothy et al. implemented a more intensive protocol that combined an initial colonoscopic infusion with repeated maintenance enemas, five times per week for 8 weeks. This approach achieved steroid-free clinical and endoscopic remission in 27% of patients receiving FMT compared with 8% in the placebo group (*p* = 0.02), without a significant increase in SAEs. The magnitude of benefit in this trial suggests that treatment intensity, defined by exposure frequency and duration, may be a key determinant of efficacy [16].

Across the UC literature, a wide range of delivery routes have been explored, including the upper digestive tract (capsules) [15,22], mid-gut delivery (naso-duodenal/jejunal tubes, gastroscopy, or transendoscopic enteral tubing [TET]) [67], and lower digestive tract (enema, colonoscopy, or colonic TET) [16,61,69]. The available evidence suggests that differences in clinical outcomes may relate more to treatment intensity and cumulative exposure than to route of administration alone. Trials reporting stronger clinical outcomes often combine an initial colonoscopic infusion (to enhance proximal colonic distribution) with repeated enemas to reinforce exposure [16,17]. Capsule-based approaches represent a different balance, offering reduced procedural burden and improved patient acceptance, but with variable dependence on pretreatment (e.g., antibiotics) and uncertain comparability with lower gastrointestinal administration. In the STOP-colitis pilot RCT, clinical response was achieved in 50% of patients receiving colonic FMT compared with 17% in the nasogastric delivery group, with a favorable safety profile and no excess in serious adverse events [14,43].

Consistent with this, protocol intensity emerged as a key determinant of clinical response. Trials using shorter or single-dose regimens generally reported lower remission rates, whereas multi-dose protocols administered over 8–12 weeks achieved remission in a larger proportion of patients [16,17,61,62,70]. More recent trials have explored alternative formulations and conditioning strategies. In the LOTUS trial, oral lyophilized FMT capsules administered after antibiotic pretreatment achieved corticosteroid-free clinical remission in 53% of patients at week 8 (*p* < 0.001), compared with 15% in the placebo group, and sustained remission was observed in a subset of patients receiving maintenance therapy [22]. In contrast, a maintenance-focused randomized trial in quiescent UC using a single colonoscopic FMT did not demonstrate a statistically significant advantage over placebo at one year, underscoring the importance of baseline disease activity and repeated exposure at the time of intervention [71].

Endoscopic outcomes generally paralleled clinical responses. Among patients achieving clinical remission, endoscopic remission or significant mucosal improvement—defined as Mayo endoscopic subscores ≤ 1—was reported in approximately 20–40% of cases [16,17,61].

Donor-related factors also appear to influence outcomes, with several studies reporting improved outcomes with unrelated or pooled donors, possibly reflecting the combined effects of rigorous screening protocols, greater microbial diversity, and enhanced representation of key functional taxa [17]. Donor microbiome composition and functional capacity appear relevant, but most clinical trials do not yet define “optimal” donor features in a way that is transferable across settings.

Pretreatment strategies may influence both microbial engraftment and clinical response to FMT in IBD. In an RCT, Costello et al. reported clinical remission in 12 of 38 patients (32%) receiving anaerobically prepared multidonor FMT compared with 3 of 35 patients (9%) in the placebo arm (*p* = 0.03) [17]. Although this trial did not directly compare aerobic versus anaerobic processing methods, the use of strict anaerobic preparation was hypothesized to preserve obligate anaerobes, such as *F. prausnitzii*, thereby enhancing microbial viability and therapeutic outcomes.

Antibiotic conditioning has been associated with higher remission rates in UC [22]. Smith et al. reported improved donor strain engraftment and clinical outcomes when antibiotics were administered pre-FMT [72], suggesting that reducing colonization resistance may enhance integration of the donor microbiota. In contrast, in a randomized pilot study, van Lingen et al. found that 3 weeks of budesonide (9 mg daily) prior to FMT did not significantly improve donor engraftment (*p* = 0.56) or clinical remission at week 14 (38% overall; *p* = 1.0). Notably, the response appeared donor-dependent (80% of responders linked to a single donor; *p* < 0.05) [19]. Detailed protocol variables and their associated outcomes across FMT trials are compiled in Supplementary Table S10.

Recipient-related factors are equally important and may explain much of the inter-study variability. Baseline inflammatory burden, degree of mucosal disruption, recent antibiotic exposure, and concomitant immunosuppressive therapy are plausible modifiers of engraftment and response [73]. Both disease extent and timing may further influence outcomes. Trials enrolling patients with left-sided or extensive UC, particularly in earlier disease stages, reported more favorable outcomes than studies dominated by long-standing or refractory disease, although these observations remain exploratory and were not uniformly assessed across trials [16,61,69,74].

Microbiome analyses provide additional biological context. In studies incorporating longitudinal microbial profiling, clinical response was consistently associated with successful donor microbiota engraftment, as reflected by increased alpha diversity and shifts in beta diversity toward donor-like profiles [16,61]. Responders frequently showed an expansion of taxa within the Firmicutes and Bacteroidetes phyla, including SCFA–producing organisms such as *F. prausnitzii* and *Roseburia* [17,70,74]. While these patterns support a link between microbial reconstitution and therapeutic response, the directionality and durability of these changes remain incompletely defined. Long-term follow-up data from FMT studies are compiled in Supplementary Table S10.

#### 3.3.2. Efficacy of FMT in Crohn’s Disease

In contrast to UC, evidence supporting FMT in CD remains limited and heterogeneous. Two small randomized trials now provide the highest-quality CD-specific evidence, though their results diverge substantially. In the multicenter, double-blind, placebo-controlled trial by Kao et al., patients with mild-to-moderate active CD received FMT via colonoscopy, followed by weekly oral capsules for 7 weeks. The study was stopped early for futility, and combined clinical and endoscopic remission at week 8 was not improved with FMT (0/15 vs. 1/11 [8.3%]) [75]. Sokol et al., by contrast, tested FMT as a maintenance strategy following steroid-induced remission, randomizing patients to single colonoscopic FMT or sham. Despite not meeting its primary endpoint of donor microbiota engraftment, steroid-free clinical remission was more frequent in the FMT group at week 10 (87.5% vs. 44%) and week 24 (50% vs. 33%) [76].

Beyond these trials, observational data show that FMT can produce short-term clinical improvement in a subset of patients with active CD [71,72,74,77,78,79]. Vaughn et al. found that 58% (11/19) achieved clinical remission after 12 weekly colonoscopic infusions, though remission required continued treatment to be maintained. Li et al. found a median sustained response of only ~4 months, reinforcing the need for repeated or sequential FMT to sustain any benefit [80], particularly in selected phenotypes such as inflammatory masses [18,72,77]. Better outcomes in colonic or ileocolonic CD compared with isolated ileal or stricturing disease have been reported, though these associations remain hypothesis-generating and require prospective confirmation [18,71,72,74,77,78,79].

#### 3.3.3. Safety and Pathogen Transmission

Across the included studies, FMT was generally well tolerated. Mild to moderate adverse events were reported in approximately 20–40% of treated patients, depending on study design and administration intensity (e.g., transient abdominal discomfort, diarrhea, bloating, flatulence, low-grade fever) [16,17,22,61,81]. These events were typically self-limited and did not require discontinuation of therapy.

SAEs were uncommon, occurring in approximately 0–5% of patients. In RCTs in UC, rates of hospitalization, disease flare, and need for escalation of medical therapy were comparable between FMT and control arms, suggesting that many SAEs reflected underlying disease activity rather than a direct treatment effect [16,17,22]. Importantly, no FMT-related mortality was reported across the 42 included studies, and colectomy rates did not differ significantly between intervention and control groups in randomized trials [16,61].

Because IBD populations frequently receive immunomodulators or biologic therapies, donor screening represents a central safety determinant. Protocols in the included studies consistently incorporated comprehensive donor assessment, including stool and blood testing to reduce transmission risk, alongside exclusion criteria targeting infectious exposures and high-risk comorbidities [16,17,22]. Fischer et al. [20] reported a case of extended-spectrum beta-lactamase (ESBL) producing *Escherichia coli* bacteremia following FMT in an immunosuppressed IBD patient, leading to enhanced screening protocols for multidrug-resistant organisms. More recent studies have increasingly relied on centralized stool banks and standardized manufacturing processes, which may enhance consistency, traceability, and safety monitoring [17,19,77].

Despite comprehensive screening and processing protocols, the potential for pathogen transmission cannot be fully eliminated, especially with repeated FMT exposure in immunosuppressed populations. Long-term safety beyond 12 months remains limited, with most studies reporting follow-up of 8–12 weeks [16,17,22,66]. This emphasizes the need to continue optimizing donor selection, processing protocols, and long-term safety surveillance as FMT is further evaluated.

## 4. Discussion

This systematic review synthesizes current evidence on alterations in the gut microbiota in IBD and on the therapeutic application of FMT. Consistent patterns emerged, characterized by reduced microbial diversity, depletion of obligate anaerobes linked to SCFA metabolism, and expansion of pathogens that thrive under inflammatory conditions [7,26,27]. Although UC and CD share a common dysbiotic framework, CD more often exhibits Enterobacteriaceae expansion and AIEC-related signals, aligning with previously reported associations [31,32,45,46]. Nonetheless, inter-study variability in both UC and CD remains substantial—driven by geography, diet, microbial sampling (stool vs. mucosa), and medication exposure—so clinical interpretation should emphasize pattern directionality and function over single-taxon claims [3,4].

Among UC and CD, Firmicutes depletion—reported in 75% of studies—and particularly the loss of *F. prausnitzii* (depleted in 90.8% of studies) were among the most reproducible findings. As a key butyrate producer with anti-inflammatory properties (including NF-κB inhibition and regulatory T-cell induction), its loss reflects not only compositional imbalance but also functional impairment [8]. Indeed, when metabolomic data were available, reduced SCFA–generating capacity, particularly butyrate, consistently paralleled dysbiosis [7,27].

Microbial alterations correlated strongly with mucosal inflammation and varied across disease phenotypes, supporting the concept that dysbiosis is both a consequence and a potential driver of disease activity. Emerging longitudinal data suggest that some alterations, including depletion of *F. prausnitzii*, may precede disease onset [34,35]. Approximately half of the studies examined microbiota composition as a predictor of treatment response or relapse, yet mechanistic understanding remains limited; baseline microbial features may influence therapeutic efficacy through immune modulation or pharmacokinetic interactions [27,35,55,56,57]. Notably, only 2 of 76 studies addressed the virome or mycobiome, highlighting major knowledge gaps [54,55,56]. The growing adoption of multi-omic approaches represents a critical step toward integrating taxonomic and functional insights and advancing microbiome-informed precision medicine.

RCT evidence supports a role for FMT in inducing remission in UC, although response rates vary widely. Treatment outcomes appear to depend less on the route of administration alone and more on the interaction between protocol intensity, donor characteristics, microbial viability, and recipient-related factors such as disease activity and extent [16,17,22]. More intensive or repeated regimens are generally associated with higher remission rates, and strategies that enhance donor engraftment may further improve response [16,17,61,62,70]. In CD, evidence remains limited and inconsistent. While short-term clinical improvement has been observed in some patients, durable remission after a single intervention is uncommon, and benefits are often transient, suggesting that repeated administrations may be necessary [71,72,74,77,78,79]. Overall, FMT is generally well tolerated, with mostly mild adverse events, but long-term safety data are scarce due to short follow-up in most studies.

Over the last few years, the microbiota in UC and CD have shifted from being viewed primarily as a disease-associated “fingerprint” to a dynamic, stage-dependent ecosystem that may contribute to risk, refine diagnosis, and inform treatment selection and prognosis. In the pre-disease phase, prospective and high-risk cohort work increasingly supports the concept that microbial and functional deviations can be detectable around clinical onset, strengthening the rationale for prevention-oriented studies that integrate host, exposome, and microbial trajectories [82]. From a diagnostic standpoint, metagenomic integration across large datasets is now being translated into scalable assays: recent work has developed and validated microbiome-derived biomarker panels for noninvasive IBD detection and discrimination, illustrating a path from sequencing signatures to clinically deployable tests (e.g., multiplex ddPCR platforms), albeit with ongoing challenges related to confounding by inflammation, medications, geography, and diet [83]. Therapeutically, it is increasingly clear that a single universal microbiota-based intervention—one that could be applied uniformly across all IBD patients and disease contexts—is unlikely, given the extent of inter-individual variability in microbial composition, functional capacity, and treatment response documented across this review. The field is therefore shifting toward stratified modulation of the microbiome. In this baseline, microbial states and multi-omics features are evaluated as effect modifiers of drug response, and where the microbiota is framed as a targetable determinant of mucosal immune tone and pharmacologic efficacy [84]. Finally, recent studies suggest that baseline fecal microbiome features can reflect disease state and may provide additional prognostic information for clinically relevant outcomes, supporting the development of risk models that combine microbiome metrics with established clinical, endoscopic, and inflammatory biomarkers [41].

Overall, these developments demonstrate that microbiome-informed management of IBD cannot rest on a single universal strategy. The significant diversity in microbial profiles—influenced by disease phenotype, inflammatory state, sampling site, medication exposure, and clinical context—underscores the need for patient stratification to enable meaningful clinical translation. Progress will depend on identifying which patient subgroups are most likely to benefit from specific microbiome-targeted interventions, supported by standardized multi-omic profiling and rigorous validation across heterogeneous real-world cohorts [85].

### 4.1. Clinical and Translation Implications

#### 4.1.1. Clinical Implications

At present, microbiome assays should be interpreted as supportive evidence rather than diagnostic endpoints. Collectively, microbial profiles are consistent (reduced diversity, depletion of butyrate producers, pathobiont enrichment). Still, the magnitude of effects and the taxa involved differ by geography, sampling, and study methodology, so routine decision-making should not rely on single-taxon findings.

A realistic near-term role is to improve risk assessment in defined patient subgroups. Baseline microbial features can complement conventional markers to identify patients at higher risk of relapse, postoperative recurrence, or complicated courses, potentially guiding surveillance interval and need for earlier treatment optimization.

Functional shifts are more clinically interpretable than taxonomy alone. When available, reduced SCFA functionality and bile-acid remodeling help frame practical advice–diet quality, avoiding unnecessary antibiotics, and supporting fiber-driven (saccharolytic) fermentation rather than protein-driven (proteolytic) metabolism.

Sample type matters for interpretation. Stool, mucosa, and luminal aspirates can yield different profiles; clinicians should interpret “microbiome results” in the context of the sample type.

#### 4.1.2. Translational Implications

Universal microbiota-based interventions are unlikely to succeed; a stratified, patient-specific approach is required. The significant differences among individuals in microbial composition, functional capacity, and treatment response mean that no single microbiome-targeted approach will likely work for all IBD patients. Efforts to translate research should focus on defining criteria for patient groups—such as disease type, inflammation level, prior medications, and initial microbial function—to identify which subgroups are most likely to benefit from specific treatments, such as FMT, dietary changes, or postbiotics.

Translation requires clinically meaningful endpoints. Beyond taxonomic shifts, studies should link alterations in the microbiota to objective inflammation, patient-reported outcomes, and long-term outcomes (relapse, hospitalizations, surgery), with clear thresholds for what constitutes a clinically relevant microbiome “response”.

Pathway-based signatures should be prioritized. Shotgun metagenomics and multi-omics shift interpretation from community composition to functional capacity, supporting biomarkers based on butyrate pathways, bile-acid transformation potential, and amino-acid/tryptophan metabolism over single taxa.

Mechanism-guided interventions represent the most actionable pathway to clinical translation. The recurrent functional pattern (reduced butyrate capacity; altered bile acids; pathobiont-favoring functions) indicates testable therapeutic targets: diet modulation, postbiotics (e.g., butyrate formulations), and bile-acid microbiome approaches.

## 5. Conclusions

In conclusion, UC and CD are consistently associated with context-dependent microbial and functional alterations closely linked to inflammatory activity and disease phenotype. FMT currently shows the strongest clinical signal in active UC, where repeated or intensive protocols outperform single-dose strategies; in CD, available data remain limited and mostly observational, with benefits often transient and insufficient to support routine use. Short-term safety has been acceptable under rigorous donor screening, but long-term safety data are sparse, and fundamental questions around donor selection, recipient stratification, and protocol standardization remain unresolved. Multi-omic profiling is beginning to clarify the functional significance of these microbial shifts and may ultimately guide patient selection and response prediction, but mechanistic understanding remains incomplete and long-term data are lacking. Addressing these gaps through standardized trial designs, validated biomarkers of engraftment and response, and adequate follow-up will be essential to realize the potential of microbiome-targeted therapies in IBD.

## Figures and Tables

**Table 1:** Comparative microbiota patterns in ulcerative colitis (UC) and Crohn’s disease (CD): descriptive synthesis.

Feature	Comparison	UC	CD
Alpha diversity			
Reduction	Tendency toward greater reduction in CD.	Frequently reduced versus healthy controls.	Frequently reduced versus healthy controls; often lowest in ileal CD and postoperative recurrence settings.
Beta diversity			
Separation from controls/community structure	Clear separation from controls is common in both.	Most studies reported distinct clustering from healthy controls.	Most studies reported distinct clustering from healthy controls.
Phylum-level changes			
Firmicutes depletion/butyrate producers	Shared depletion in UC and CD.	Frequent depletion of Firmicutes and other butyrate-associated taxa.	Frequent depletion of Firmicutes and butyrate producers, often more marked in ileal disease
Proteobacteria/Enterobacteria expansion	Shared expansion linked to inflammation; signal more frequent in CD.	Frequent enrichment, particularly with active disease.	Frequent enrichment, often more pronounced in active and ileal CD.
Key depleted taxa			
*F. prausnitzii*	Among the most reproducible depleted taxa in both diseases.	Frequently reduced; lower abundance often tracked active inflammation and less favorable trajectories.	Frequently reduced; depletion was often highlighted in ileal CD, postoperative disease, and active inflammation.
*Roseburia* spp.	Shared depletion; no clear disease-specific predominance.	Frequently reported.	Frequently reported.
Clostridium clusters IV/XIVa	Shared depletion, with a possible trend toward greater depletion in CD.	Frequently reported.	Frequently reported, possibly more pronounced.
Key exapanded taxa			
*Escherichia coli*	Expanded in both UC and CD; evidence suggests greater prominence in CD.	Frequently reported.	Frequently reported; often more prominent in CD, particularly in inflammatory and ileal phenotypes.
*E. coli* magnitude	Available studies suggest higher abundance in CD than UC.	Increased vs. controls.	Increased vs. controls, with a trend toward higher abundance.
*Fusobacterium* spp.	Best interpreted as a context-dependent inflammatory signal rather than a robust UC/CD discriminator.	Reported in a subset of studies.	Reported in a subset of studies.
*Enterococcus* spp.	Expanded in both diseases, with a possible trend toward greater frequency in CD	Reported in a subset of studies.	Reported in a subset of studies, possibly more frequent.
Disease specific signals			
AIEC *E. coli*	*E. coli* enrichment occurs in both; AIEC signal is much more characteristic of CD.	*E. coli* enrichment is reported.	*E. coli* enrichment is recurrent, and AIEC-related signals are more prominent than in UC.
*R. gnavus* enrichment	Phenotype-linked signal; not disease-defining	Some UC cohorts reported enrichment, particularly in active inflammation.	Also reported in CD.
*Bacteroides fragilis* depletion	Strain-specific; depletion of protective *B. fragilis* appears more consistent in CD.	Reduced in a subset of studies.	More consistently reduced.
Muccus associated taxa (e.g., *Akkermansia muciniphila* depletion)	Altered in both; evidence suggests a stronger signal in CD.	Reduced in a subset of studies.	Reduced in a subset of studies.
*Campylobacter* spp. enrichment	Not a robust disease discriminator.	Reported in a minority of studies.	Reported in a minority of studies.
Functional alterations			
Reduced SCFA-producing capacity	Shared functional consequence of dysbiosis in UC and CD, with possible greater impairment in CD	Reported in a subset of studies; butyrate-related impairment recurrent	Reported in a subset of studies; butyrate-related impairment recurrent, possibly more pronounced
Bile acid metabolism disruption	Evidence is stronger in CD, especially ileal disease.	Reported in UC, but less consistently.	More consistently disturbed, particularly in ileal disease.
Clinical associations			
Association with activity/biomarker	Dysbiosis tracks mucosal inflammation in both diseases.	Active disease is generally associated with lower diversity, loss of commensals, and enrichment of facultative/pathobiont taxa.	Active disease shows the same pattern, often with stronger Enterobacteriaceae/AIEC-related signals.
Relapse prediction	Exploratory signal only; not ready for stand-alone use.	Higher diversity and commensal-enriched states were linked to better outcomes in a subset of studies.	Similar exploratory findings were reported in postoperative and biologic-response settings.

Abbreviations: AIEC, adherent-invasive *Escherichia coli*; CD, Crohn’s disease; SCFA, short-chain fatty acids; UC, ulcerative colitis. Notes: This table presents a descriptive synthesis rather than a quantitative meta-analysis. Disease-specific evidence was drawn from UC studies (*n* = 68) and CD studies (*n* = 63); 37 comparative studies included both diseases and contributed to both disease-specific columns only when subgroup-specific findings were extractable. Comprehensive data stratified by disease type are summarized in Supplementary Table S5 (UC) and Supplementary Table S6 (CD).

**Table 2:** Pivotal FMT studies in IBD.

Study, Year	Design, N	Core Protocol	Main Result	Key Notes
Ulcerative colitis (UC)				
Rossen, 2015	RCT, *n* = 48	Nasoduodenal; 2 doses	No significant benefit vs. autologous stool	Early negative study. Highlights route/protocol limitations
Moayyedi, 2015	RCT, *n* = 75	Enema; 6 weekly doses	Clinical/endoscopic remission 24% vs. 5%	First positive RCT. weekly enemas effective.
Paramsothy, 2017	RCT, *n* = 81	Colonoscopy + intensive enemas over 8 weeks	Steroid-free clinical/endoscopic remission 27% vs. 8%,	Landmark intensive multidonor UC trial
Costello, 2019	RCT, *n* = 73	Colonoscopy + enemas over 8 weeks	Clinical/endoscopic remission: 32% vs. 9%	Key trial supporting multidose lower-GI delivery. Anaerobic preparation.
Haifer (LOTUS), 2022	RCT, *n* = 35	Oral lyophilized FMT after antibiotic pretreatment	Clinical remission/repsonse week 8 53% vs. 15%	Oral capsules strategy
Lahtinen, 2023	RCT, *n* = 48	Single colonoscopic FMT for maintenance in quiescent UC	Primary endpoint 54% vs. 41%, not significant	Maintenance study
Crohn’s disease (CD)				
Vaughn, 2016	Prospective cohort, *n* = 19 NOS 7/9	Repeated colonoscopic FMT; 12 weekly doses	Clinical remission 58% (11/19)	Repeated dose CD cohort. Includes donor-like microbiota shift
Li, 2019	Prospective cohort, *n* =32	Repeated colonoscopic FMT	Clinical response: 56% (18/32). Median sustained benefit ~4 m.	Durability and need for repeat FMT
Kao, 2024	RCT, *n* = 32	Colonoscopic FMT + weekly oral capsules for 7 weeks	Combined clinical/endoscopic response: 0 vs. 8.3%, not significant	CD randomized evidence

Abbreviations: FMT, fecal microbiota transplantation; IBD, inflammatory bowel disease, RCT, randomized controlled trial; UC, ulcerative colitis; CD, Crohn’s disease; Note: Only pivotal studies most relevant to efficacy, delivery strategy, and durability are shown in the main text. Additional protocol-refinement, mechanistic, and phenotype-specific studies are reported in Supplementary Table S9. Outcome definitions varied across studies and should be interpreted according to each trial’s prespecified endpoint.

## Data Availability

The original contributions presented in this study are included in the article/Supplementary Materials. Further inquiries can be directed to the corresponding author.

## Supplementary Materials

The following supporting information can be downloaded at: https://www.mdpi.com/article/10.3390/pathogens15040451/s1, Supplementary Materials S1: Search Strategy and PRISMA 2020 Checklist; Table S1: PRISMA 2020 Checklist; Table S2: Risk of Bias and Quality Assessment; Table S3: Full-Text articles excluded after Eligibility; Table S4: Characteristics of Included Microbiota Studies; Table S5: Microbiota Alterations in UC; Table S6: Microbiota Alterations in CD; Table S7: Treatment Response and Microbiota Predictors; Table S8: Multi-omics and Metabolomics Findings in IBD Microbiota Studies; Table S9: Characteristics of Included FMT Studies; Table S10: Long-term Follow-up Data.

## Abbreviations

The following abbreviations are used in this manuscript:

16S rRNA	16S ribosomal RNA
AIEC	adherent-invasive *Escherichia coli*
AUC	area under the curve
CD	Crohn’s disease
CI	confidence interval
CRP	C-reactive protein
ddPCR	droplet digital polymerase chain reaction
fCP	fecal calprotectin
FMT	fecal microbiota transplantation
FMT-A	autologous fecal microbiota transplantation
FMT-D	donor fecal microbiota transplantation
FXR	farnesoid X receptor
HR	hazard ratio
IBD	inflammatory bowel disease
IL-12/23	interleukin-12/23
ITT	intention-to-treat
JAK	Janus kinase
MeSH	Medical Subject Headings
NF-κB	nuclear factor kappa B
NOS	Newcastle–Ottawa Scale
NR	not reported
OR	odds ratio
PCoA	principal coordinates analysis
PERMANOVA	permutational multivariate analysis of variance
PRISMA	Preferred Reporting Items for Systematic Reviews and Meta-Analyses
PSC	primary sclerosing cholangitis
R^2^	coefficient of determination
RCT	randomized controlled trial
RoB	risk of bias
SAE	serious adverse event
SCFA	short-chain fatty acid(s)
S1P	sphingosine-1-phosphate
TET	transendoscopic enteral tubing
TGR5	Takeda G-protein–coupled receptor 5 (GPBAR1)
TNF	tumor necrosis factor
UC	ulcerative colitis
