# Supplementary material for: The Role of Microbiota and Fecal Transplantation in Inflammatory Bowel Disease
Source: Pathogens. 2026 Apr 21;15(4):451. doi: 10.3390/pathogens15040451 (PMC13119295; doi:10.3390/pathogens15040451)
Supplement: Supplementary file 1 [file pathogens-15-00451-s001.zip › pathogens-4204418-supplementary.pdf]

pathogens

MDPI

Supplementary Materials

The Role of Microbiota and Fecal Transplantation in Inflammatory Bowel Disease

Supplementary Materials 1. Search Strategy and PRISMA 2020 Checklist

1.1 Databases and Sources Searched

A systematic literature search was performed across three primary electronic databases supplemented by manual reference screening. All records were managed and deduplicated in SciSpace (automated + manual verification).

1.2 Full PubMed/MEDLINE Search String

Database: PubMed (National Library of Medicine). Filters: Humans; English language; no date or article-type restrictions.

```
(("inflammatory bowel disease"[MeSH Terms] OR "inflammatory bowel disease"[Title/Abstract] OR
"ulcerative colitis"[MeSH Terms] OR "ulcerative colitis"[Title/Abstract] OR
"Crohn disease"[MeSH Terms] OR "Crohn's disease"[Title/Abstract] OR
"Crohns disease"[Title/Abstract] OR IBD[Title/Abstract] OR
UC[Title/Abstract] OR CD[Title/Abstract])
AND
("gastrointestinal microbiome"[MeSH Terms] OR "microbiota"[Title/Abstract] OR
"microbiome"[Title/Abstract] OR "dysbiosis"[Title/Abstract] OR
"gut bacteria"[Title/Abstract] OR "intestinal microbiota"[Title/Abstract] OR
"fecal microbiota transplantation"[MeSH Terms] OR
"fecal microbiota transplantation"[Title/Abstract] OR
"faecal microbiota transplantation"[Title/Abstract] OR FMT[Title/Abstract] OR
"stool transplant"[Title/Abstract] OR "bile acids"[MeSH Terms] OR "bile acids"[Title/Abstract])
AND
("16S ribosomal RNA"[MeSH Terms] OR "16S rRNA"[Title/Abstract] OR "16S"[Title/Abstract] OR
"metagenomics"[MeSH Terms] OR "metagenomics"[Title/Abstract] OR "metagenomic"[Title/Abstract] OR
"high-throughput nucleotide sequencing"[MeSH Terms] OR
"next-generation sequencing"[Title/Abstract] OR NGS[Title/Abstract] OR
"sequencing"[Title/Abstract] OR "randomized controlled trial"[Publication Type] OR
"clinical trial"[Publication Type]))
```

1.3 Cochrane CENTRAL Search String

Database: Cochrane Library (cochranelibrary.com). Filters: All trials; English; no date restrictions.

```
#1  MeSH descriptor: [Inflammatory Bowel Diseases] explode all trees
#2  (inflammatory bowel disease):ti,ab,kw OR (ulcerative colitis):ti,ab,kw OR
```

 https://doi.org/10.3390/pathogens15040451

```
(Crohn* disease):ti,ab,kw OR IBD:ti,ab,kw OR UC:ti,ab,kw
#3  #1 OR #2
#4  MeSH descriptor: [Gastrointestinal Microbiome] explode all trees
#5  (microbiota):ti,ab,kw OR (microbiome):ti,ab,kw OR (dysbiosis):ti,ab,kw OR
    (gut bacteria):ti,ab,kw OR (bile acids):ti,ab,kw
#6  MeSH descriptor: [Fecal Microbiota Transplantation] explode all trees
#7  (fecal microbiota transplantation):ti,ab,kw OR (faecal microbiota transplantation):ti,ab,kw
    OR FMT:ti,ab,kw OR (stool transplant):ti,ab,kw
#8  #4 OR #5 OR #6 OR #7
#9  (16S):ti,ab,kw OR (metagenomics):ti,ab,kw OR (sequencing):ti,ab,kw
#10 #8 OR #9
#11 #3 AND #10
```

1.4 Google Scholar Queries

Platform: scholar.google.com. Top 200 results per query screened by relevance (December 2025).

Query 1: "inflammatory bowel disease" OR "ulcerative colitis" OR "Crohn's disease" microbiota OR microbiome 16S sequencing

Query 2: "fecal microbiota transplantation" OR FMT "ulcerative colitis" OR "Crohn's disease" randomized controlled trial OR RCT

1.5 Eligibility Criteria

Inclusion criteria:

- Human subjects ≥ 18 years with confirmed IBD (ulcerative colitis or Crohn's disease)
- Clinical trials and observational studies on microbiome composition, metabolites, microbiota-targeted interventions, or FMT
- Original research articles published in English

Exclusion criteria:

- Studies not primarily focused on IBD and the gut microbiome
- Non-intestinal microbiome investigations (blood, skin, urogenital)
- Review articles, editorials, conference abstracts, case reports (<5 patients)
- Studies with insufficient data or published in non-English language
- Animal studies, in vitro studies, organoid models

1.6 Study Selection and Data Extraction

Two authors (I.L. and E.P.A.) independently screened all records using predefined inclusion/exclusion criteria. Full-text articles were subsequently assessed for eligibility. Data extraction was performed independently, with discrepancies resolved by consensus. A third reviewer was consulted for unresolved disagreements.

1.7 PRISMA 2020 Study Selection Flow

Table S1.1. Results of the systematic search and study selection process in accordance with PRISMA 2020 (Page et al., BMJ 2021). A flow diagram is presented as Figure 1 in the main manuscript.

Stage	n
Records identified from databases (PubMed, Cochrane, Google Scholar)	632
Records identified from other sources (manual reference screening)	15
**Total records identified**	**647**
Duplicates removed	317
Records screened (title and abstract)	315
Records excluded at title/abstract stage	152
– Inappropriate study design or population	98
– Non-primary focus on microbiota/IBD	34
– Ineligible publication type	20
Full-text articles assessed for eligibility	163
Full-text articles excluded	45
– Insufficient microbiome data	18
– No extractable clinical outcomes	13
– Case reports	8
– Non-English language	6
**Studies included in qualitative synthesis**	**118**
– Microbiota profiling studies	76
– FMT intervention studies (RCTs and observational)	42

Supplementary Table S1. PRISMA 2020 Checklist

Preferred Reporting Items for Systematic Reviews and Meta-Analyses (PRISMA) 2020 checklist (Page et al., BMJ 2021;372:n71). All items are addressed in the manuscript unless otherwise noted.

Section / Topic	Item #	Checklist item	Location where item is reported
TITLE			
Title	1	Identify the report as a systematic review.	*Title page (p1)*
ABSTRACT			
Abstract	2	See the PRISMA 2020 for Abstracts checklist.	*Abstract (p1)*
INTRODUCTION			
Rationale	3	Describe the rationale for the review in the context of existing knowledge.	*Introduction (p2-2)*
Objectives	4	Provide an explicit statement of the objective(s) or question(s) the review addresses.	*Intro - Objectives (pp 2-3)*
METHODS			
Eligibility criteria	5	Specify the inclusion and exclusion criteria for the review and how studies were grouped for the syntheses.	*Methods - Eligibility (p3)*
Information sources	6	Specify all databases, registers, websites, organisations, reference lists and other sources searched or consulted to identify studies. Specify the date when each source was last searched or consulted.	*Methods (p2); Suppl Appendix 1*
Search strategy	7	Present the full search strategies for all databases, registers and websites, including any filters and limits used.	*Methods (p2); Suppl Appendix 1*
Selection process	8	Specify the methods used to decide whether a study met the inclusion criteria of the review, including how many reviewers screened each record and each report retrieved, whether they worked independently, and if applicable, details of automation tools used in the process.	*Methods - (p3)*
Data collection process	9	Specify the methods used to collect data from reports, including how many reviewers collected data from each report, whether they worked independently, any processes for obtaining or confirming data from study investigators, and if applicable, details of automation tools used in the process.	*Methods (p3); Suppl Appendix 1*
Data items	10a	List and define all outcomes for which data were sought. Specify whether all results that were compatible with each outcome domain in each study were sought (e.g. for all measures, time points, analyses), and if not, the methods used to decide which results to collect.	*Methods (p3)*

Data items	**10b**	List and define all other variables for which data were sought (e.g. participant and intervention characteristics, funding sources). Describe any assumptions made about any missing or unclear information.	*Methods –Quality assessment (p3)*
Study risk of bias assessment	**11**	Specify the methods used to assess risk of bias in the included studies, including details of the tool(s) used, how many reviewers assessed each study and whether they worked independently, and if applicable, details of automation tools used in the process.	*Methods - quality assessment (p3)*
Effect measures	**12**	Specify for each outcome the effect measure(s) (e.g. risk ratio, mean difference) used in the synthesis or presentation of results.	*Methods (p3)*
Synthesis methods	**13a**	Describe the processes used to decide which studies were eligible for each synthesis (e.g. tabulating the study intervention characteristics and comparing against the planned groups for each synthesis (item #5)).	*Methods – (p3)*
Synthesis methods	**13b**	Describe any methods required to prepare the data for presentation or synthesis, such as handling of missing summary statistics, or data conversions.	*Methods (p3)*
Synthesis methods	**13c**	Describe any methods used to tabulate or visually display results of individual studies and syntheses.	*Results (pp8-12); Suppl materials*
Synthesis methods	**13d**	Describe any methods used to synthesize results and provide a rationale for the choice(s). If meta-analysis was performed, describe the model(s), method(s) to identify the presence and extent of statistical heterogeneity, and software package(s) used.	*Methods – no meta-analysis was performed*
Synthesis methods	**13e**	Describe any methods used to explore possible causes of heterogeneity among study results (e.g. subgroup analysis, meta-regression).	*Methods - Subgroup analyses (p6)*
Synthesis methods	**13f**	Describe any sensitivity analyses conducted to assess robustness of the synthesized results.	*Methods (p3)*
Reporting bias assessment	**14**	Describe any methods used to assess risk of bias due to missing results in a synthesis (arising from reporting biases).	*Methods (p3)*
Certainty assessment	**15**	Describe any methods used to assess certainty (or confidence) in the body of evidence for an outcome.	*Methods (p3)*
**RESULTS**			
Study selection	**16a**	Describe the results of the search and selection process, from the number of records identified in the search to the number of studies included in the review, ideally using a flow diagram.	*Results – Study selection (p3–4); Fig 1; Suppl Appendix 1*
Study selection	**16b**	Cite studies that might appear to meet the inclusion criteria, but which were excluded, and explain why they were excluded.	*Results – Study selection (p3–4); Fig 1; Suppl Table S5*

Study characteristics	**17**	Cite each included study and present its characteristics.	*Results (pp3–17); Table 1; Suppl Tables S2–S3*
Risk of bias in studies	**18**	Present assessments of risk of bias for each included study.	*Results (pp3–17); Suppl Table S4*
Results of individual studies	**19**	For all outcomes, present, for each study: (a) summary statistics for each group (where appropriate) and (b) an effect estimate and its precision (e.g. confidence/credible interval), ideally using structured tables or plots.	*Results (pp3–17); Suppl Tables S2–S3*
Results of syntheses	**20a**	For each synthesis, briefly summarise the characteristics and risk of bias among contributing studies.	*Results (pp3–17)*
Results of syntheses	**20b**	Present results of all statistical syntheses conducted. If meta-analysis was done, present for each the summary estimate and its precision (e.g. confidence/credible interval) and measures of statistical heterogeneity. If comparing groups, describe the direction of the effect.	*Results (p 3-17); Table 1 – No meta-analysis*
Results of syntheses	**20c**	Present results of all investigations of possible causes of heterogeneity among study results.	*Results - Subgroup analyses (p 6-7)*
Results of syntheses	**20d**	Present results of all sensitivity analyses conducted to assess the robust-ness of the synthesized results.	*Results (pp3–17)*
Reporting biases	**21**	Present assessments of risk of bias due to missing results (arising from reporting biases) for each synthesis assessed.	*Results (pp3–17); Suppl Table S4*
Certainty of evidence	**22**	Present assessments of certainty (or confidence) in the body of evidence for each outcome assessed.	*Results; Suppl Table S4*
**DISCUSSION**			
Discussion	**23a**	Provide a general interpretation of the results in the context of other evi-dence.	*Discussion (p 17-18)*
Discussion	**23b**	Discuss any limitations of the evidence included in the review.	*Discussion (p 17-18)*
Discussion	**23c**	Discuss any limitations of the review processes used.	*Discussion (p 17-18)*
Discussion	**23d**	Discuss implications of the results for practice, policy, and future re-search.	*Clinical and translational impli-cations (p 18-19)*
**OTHER INFOR-MATION**			
Registration and pro-tocol	**24a**	Provide registration information for the review, including register name and registration number, or state that the review was not registered.	*Not registered*
Registration and pro-tocol	**24b**	Indicate where the review protocol can be accessed, or state that a pro-tocol was not prepared.	*Protocol nor prepared*

Registration and protocol	**24c**	Describe and explain any amendments to information provided at registration or in the protocol.	*Protocol nor prepared*
Support	**25**	Describe sources of financial or non-financial support for the review, and the role of the funders or sponsors in the review.	*No funding (p19-20)*
Competing interests	**26**	Declare any competing interests of review authors.	*Conflicts of interest (p 19-20)*
Availability of data, code and other materials	**27**	Report which of the following are publicly available and where they can be found: template data collection forms; data extracted from included studies; data used for all analyses; analytic code; any other materials used in the review.	*Supplementary Tables S1–S5 (provided with submission).*

Reference: Page MJ, McKenzie JE, Bossuyt PM, et al. The PRISMA 2020 statement: an updated guideline for reporting systematic reviews. ***BMJ****. 2021;372:n71. doi:10.1136/bmj.n71*

1.8 Quality Assessment

Risk of bias in RCTs was assessed using Cochrane Risk of Bias 2 (RoB 2). Observational studies were assessed using the Newcastle–Ottawa Scale (NOS). Both assessments were performed independently by two reviewers; disagreements were resolved by consensus.

RCTs (n=16) — RoB 2 summary:

Low risk of bias: 2 studies (12.5%); Some concerns: 14 studies (87.5%); High risk: 0 studies. Main concerns: blinding challenges (8 studies) and missing outcome data (6 studies).

Observational studies (n=102) — NOS summary:

Microbiota studies (n=76): Good quality (NOS ≥7): 5 (6.6%); Fair quality (NOS 4–6): 38 (50.0%); Poor quality (NOS ≤3): 33 (43.4%). Observational FMT studies (n=26): High quality: 16 (61.5%); Moderate: 9 (34.6%); Low: 1 (3.8%).

Supplementary Table S2. Risk of Bias and Quality Assessment

Risk of bias in randomised controlled trials (n=38) was assessed using the Cochrane Risk of Bias 2 (RoB 2) tool. Quality of observational microbiota studies (n=76) was assessed using the Newcastle–Ottawa Scale (NOS). Both assessments were performed independently by two reviewers; inter-rater agreement: Cohen's κ = 0.856 (95.2% agreement). Disagreements resolved by consensus.

SECTION A — Cochrane Risk of Bias 2 (RoB 2): FMT Randomised Controlled Trials (n=38)						
Study	Randomization Process	Deviations from Interventions	Missing Outcome Data	Measurement of Outcome	Selection of Reported Result	Overall Risk of Bias
Moayyedi P 2015	Some concerns	Low	Low	Low	Low	**Some concerns**
Rossen NG 2015	Low	Low	Some concerns	Low	Low	**Some concerns**
Paramsothy S 2017	Some concerns	Some concerns	Low	Low	Low	**Some concerns**
Costello SP 2019	Low	Low	Some concerns	Low	Low	**Some concerns**
Sood A 2019	Some concerns	Some concerns	Low	Low	Some concerns	**Some concerns**
Haifer C 2021	Low	Low	Some concerns	Low	Some concerns	**Some concerns**
Crothers JW 2021	Low	Low	Low	Low	Low	**Low**
Ding X 2021	Low	Some concerns	Low	Low	Some concerns	**Some concerns**

Baunwall SMD 2022	Some concerns	Some concerns	Low	Low	Low	**Some concerns**
Ooijevaar RE 2023	Low	Low	Some concerns	Low	Some concerns	**Some concerns**
Segal JP 2023	Some concerns	Low	Some concerns	Low	Low	**Some concerns**
Allegretti JR 2024	Some concerns	Low	Low	Low	Low	**Some concerns**
Johnsen PH 2024	Low	Some concerns	Low	Low	Some concerns	**Some concerns**
Zhang T 2024	Low	Some concerns	Low	Low	Some concerns	**Some concerns**
Sokol H 2020	Some concerns	Low	Some concerns	Low	Low	**Some concerns**
Angelberger S 2013	Some concerns	High	High	Some concerns	Some concerns	**High**
Kunde S 2013	High	Some concerns	Some concerns	Some concerns	High	**High**
Kump PK 2013	High	High	Some concerns	Some concerns	High	**High**
Vermeire S 2016	Low	Some concerns	Low	Low	Low	**Some concerns**
Nishida A 2017	Low	Low	Low	Low	Some concerns	**Some concerns**
Borody TJ 2003	High	High	Some concerns	Some concerns	High	**High**
Damman CJ 2015	Low	Low	Low	Low	Low	**Low**
Ishikawa D 2017	Some concerns	Some concerns	Some concerns	Low	Some concerns	**Some concerns**
Pai N 2021	Low	Some concerns	Some concerns	Low	Low	**Some concerns**
Schmartz GP 2021	Some concerns	Low	Some concerns	Low	Some concerns	**Some concerns**
Danne C 2021	Low	Some concerns	Low	Low	Some concerns	**Some concerns**
Haifer C 2022	Low	Some concerns	Some concerns	Low	Low	**Some concerns**
Vaughn BP 2023	Some concerns	Some concerns	Some concerns	Low	Low	**Some concerns**

Vaughn BP 2016	Low	Low	Some concerns	Low	Some concerns	**Some concerns**
He Z 2017	Low	Low	Low	Low	Some concerns	**Some concerns**
Sokol H 2017	Low	Low	Low	Low	Low	**Low**
Goyal A 2018	Some concerns	Some concerns	Low	Low	Low	**Some concerns**
Allegretti JR 2020	Some concerns	Some concerns	Some concerns	Low	Some concerns	**Some concerns**
Fischer M 2016	Some concerns	Some concerns	Low	Low	Some concerns	**Some concerns**
Allegretti 2025	High	High	High	Some concerns	Some concerns	**High**
Landy J 2019	Low	Low	Low	Low	Low	**Low**
Sood A 2021	Some concerns	Low	Some concerns	Low	Low	**Some concerns**
Costello SP 2021	Low	Low	Low	Low	Low	**Low**
**Summary (n=38): Low risk: 5 (13.2%) | Some concerns: 28 (73.7%) | High risk: 5 (13.2%)**						

Supplementary Table S2 (continued). Newcastle–Ottawa Scale — Microbiota Studies

SECTION B — Newcastle–Ottawa Scale (NOS): Observational Microbiota Studies (n=76)									
Study ID	First Author	Year	Study Design	Selection (0–4)	Comparability (0–2)	Outcome (0–3)	Total NOS (0–9)	Quality Rating	Justification
S001	Dorffel	2012	Cross-sectional	2	0	2	4	**Fair**	SELECTION (2/4): Cross-sectional multi-group study (n=66) comparing NET subtypes, CD patients, and healthy controls; CD enrolled as disease comparator, not from a dedicated IBD cohort, limiting IBD-specific representativeness; FISH provided validated quantification of pre-specified bacterial targets; no disease activity stratification within the CD subgroup. COMPARABILITY (0/2): Medication use noted but no statistical adjustment for age, sex, or disease severity. OUTCOME (2/3): Objective FISH-based bacterial counts; no longitudinal follow-up by cross-sectional design.
S002	Liguori	2016	Observational	3	1	1	5	**Fair**	SELECTION (3/4): Study of 23 participants (CD in active flare and remission, plus healthy controls) using mucosal biopsies from the right colon; disease state defined by clinical and endoscopic criteria; combined 16S rRNA and fungal ITS sequencing extends standard bacterial profiling; limited sample size reduces generalizability. COMPARABILITY (1/2): Controlled for disease activity (flare vs remission); concomitant medications not formally adjusted for. OUTCOME (1/3): Mucosal biopsy characterization; disease-free status at baseline not independently confirmed; no prospective follow-up period.
S003	Majzoub	2024	RCT	4	2	3	9	**Good**	SELECTION (4/4): Prospective cohort using a well-characterized multicenter UC population from the Paramsothy 2017 FMT trial; healthy donors served as controls; validated shotgun metagenomics applied with a standardized bioinformatics pipeline; baseline disease status documented by clinical, endoscopic, and microbiome assessments. COMPARABILITY (2/2): Randomized parent trial design controlled for known and unknown confounders; stratified analysis by key clinical variables. OUTCOME (3/3): Prospective longitudinal microbiome sampling at pre-defined clinical timepoints; validated clinical endpoints; adequate follow-up duration with assessor blinding.

S005	Angriman	2024	RCT	1	0	2	3	**Poor**	SELECTION (1/4): Single-center RCT of 52 post-proctocolectomy UC patients with IPAA at ileostomy closure; highly specific surgical phenotype limits representativeness to standard IBD outpatient populations; no healthy control group for microbiota comparison; validated 16S rRNA sequencing. COMPARABILITY (0/2): Trial randomization provided overall balance, but microbiota subgroup analyses lacked covariate adjustment; concomitant pouchitis treatment not consistently reported. OUTCOME (2/3): Validated 16S rRNA sequencing at T0 and T1 (8 weeks); adequate follow-up for intervention endpoint; outcome assessors not formally blinded to microbiome results.
S006	Bajer	2017	Observational	1	0	2	3	**Poor**	SELECTION (1/4): Study comparing PSC-IBD and UC patients vs controls (total n=32, 11 controls); study design not clearly reported; patient selection criteria and disease definitions not formally described; 16S rRNA sequencing applied to a small mixed cohort. COMPARABILITY (0/2): No confounders identified or statistically adjusted for; PSC co-disease not accounted for in microbiota models. OUTCOME (2/3): Validated 16S rRNA sequencing; disease-free status at enrolment not confirmed; no longitudinal follow-up.
S007	Bak	2025	Cohort	1	0	2	3	**Poor**	SELECTION (1/4): Prospective cohort of 60 CD patients after ileocolic resection, followed for anastomotic complications; population restricted to a postoperative surgical phenotype, limiting generalizability to non-surgical IBD; no healthy controls; baseline microbiome and clinical status documented before ileoscopy. COMPARABILITY (0/2): No formal adjustment for confounders such as resection extent, antibiotic use, or concomitant biological therapy. OUTCOME (2/3): Validated 16S rRNA sequencing on mucosal biopsies at standardized postoperative colonoscopy; prospective outcome assessment; blinding of outcome assessors not stated.
S009	Borren	2021	Cohort	1	1	2	4	**Fair**	SELECTION (1/4): Prospective cohort of 166 IBD patients (UC and CD) in clinical remission recruited from a single IBD clinic; without a healthy control arm, the fatigue-microbiota analysis is limited to IBD comparisons; convenience sampling from tertiary referral limits community representativeness; shotgun metagenomics applied. COMPARABILITY (1/2): Controlled for disease activity (quiescent IBD); no adjustment for IBD subtype, concomitant medication, or fatigue severity scale. OUTCOME (2/3): Validated shotgun metagenomics with standardized stool processing; prospective design with objective biomarkers (fCal); no long-term follow-up beyond cross-sectional sampling.

S010	Brand	2021	Cross-sectional	2	1	2	5	**Fair**	SELECTION (2/4): Cross-sectional study of 99 IBD-discordant and -concordant twin pairs (Netherlands) plus unrelated healthy controls; twin design provides partial genetic and early environmental matching; specialty clinic recruitment limits representativeness to IBD-diagnosed and genetically predisposed individuals; validated shotgun metagenomics. COMPARABILITY (1/2): Twin design reduces genetic confounding; age and sex comparable by design within pairs; no statistical adjustment for disease duration or medication. OUTCOME (2/3): Validated shotgun metagenomics; cross-sectional design with no longitudinal follow-up; objective sample processing.
S011	Chen	2017	Case-control	1	0	1	2	**Poor**	SELECTION (1/4): Small case-control study of 8 UC patients and 8 matched healthy partners from a single center (Lishui); partnership matching provides some control for shared household environment; shotgun metagenomics applied; very small sample severely limits statistical power and generalizability. COMPARABILITY (0/2): Partner-matching for shared living environment; no adjustment for disease activity, medication, or dietary differences. OUTCOME (2/3): Validated shotgun metagenomics; no longitudinal follow-up; cross-sectional outcome assessment.
S012	Chen	2024	Observational	3	1	2	6	**Fair**	SELECTION (3/4): Case-control discovery cohort (n=148: active/quiescent CD vs sibling controls) with two independent replication cohorts; paired sibling design partially controls for genetic confounders; validated shotgun metagenomics; CD phenotyping by rigorous clinical and endoscopic criteria. COMPARABILITY (1/2): Controlled for disease activity (active vs quiescent); sibling design accounts for some genetic and environmental confounders; no full multivariate adjustment for medication in all analyses. OUTCOME (2/3): Validated shotgun metagenomics across multiple cohorts; cross-sectional design; no long-term prospective follow-up.
S013	Hernandez Rocha	2025	Cohort	1	1	2	4	**Fair**	SELECTION (1/4): Prospective cohort of 262 CD patients in remission after surgical resection followed for endoscopic recurrence; population and recruitment center not fully described, limiting replication; no healthy controls; shotgun metagenomics applied to a defined postoperative phenotype. COMPARABILITY (1/2): Controlled for disease activity (surgical remission confirmed); no formal multivariate adjustment for other confounders such as smoking, medication, or disease location. OUTCOME (2/3): Validated shotgun metagenomics; prospective follow-up with standardized endoscopic recurrence endpoint; outcome assessment not stated as blinded.

S014	Clooney	2021	Cohort	2	2	3	7	**Good**	SELECTION (2/4): Large longitudinal cohort (n=692) of IBD patients and non-IBD controls from Ireland and Canada; 16S rRNA sequencing with repeated sampling over multiple disease states; geographic dual-site recruitment introduces variability; no formal population-based sampling frame. COMPARABILITY (2/2): Mixed-effects models controlled for disease activity, geographic site, and medication; individual-level longitudinal design reduces between-subject confounding. OUTCOME (3/3): Validated 16S rRNA sequencing at multiple timepoints; adequate longitudinal follow-up across disease transitions; objective and standardized sample processing.
S015	Cook	2020	Cross-sectional	1	0	0	1	**Poor**	SELECTION (1/4): Cross-sectional study with three cohorts (IBD: CD n=55, UC n=9 in cohort 1; CD n=20, UC n=20, HC in cohort 3); small UC subgroup in cohort 1 limits UC-specific conclusions; flagellin-specific immune assay used as proxy for microbiota exposure without validated sequencing; single-center recruitment. COMPARABILITY (0/2): No statistical adjustment for disease activity, medication, or confounders in microbiota analyses. OUTCOME (0/3): Flagellin antibody not a validated microbiota profiling method; cross-sectional design; no follow-up component.
S016	Cox	2020	RCT	1	1	2	4	**Fair**	SELECTION (1/4): Single-blind RCT of 52 quiescent IBD patients (UK) evaluating low-FODMAP diet vs control; microbiome was a secondary endpoint with no healthy reference arm; population restricted to clinically quiescent IBD with persistent functional symptoms; validated shotgun metagenomics applied. COMPARABILITY (1/2): Randomized allocation; controlled for disease activity (quiescent); no multivariate adjustment for IBD subtype or baseline microbiome composition. OUTCOME (2/3): Validated shotgun metagenomics at pre-defined timepoints; disease-free status confirmed at enrolment; no long-term follow-up beyond 4-week dietary intervention.
S017	Dicksved	2008	Cross-sectional	1	0	1	2	**Poor**	SELECTION (1/4): Cross-sectional twin case-control study of 10 monozygotic CD-discordant twin pairs plus concordant and healthy twin pairs; twin genetic matching is a strength but small sample limits generalizability; T-RFLP fingerprinting provides lower taxonomic resolution than modern 16S rRNA sequencing; single-country cohort. COMPARABILITY (0/2): Genetic control through twin design; no statistical adjustment for disease activity, disease location, or medication. OUTCOME (2/3): Validated T-RFLP profiling; cross-sectional design with no follow-up; objective and reproducible sample processing.

S018	Facchin	2025	RCT	1	0	2	3	**Poor**	SELECTION (1/4): Double-blind RCT of 140 IBD adults (UC and CD) evaluating oral butyrate (BLM) vs placebo; microbiome was a secondary endpoint with no healthy reference group; enterotype-stratified design but limited representativeness for general IBD populations; 16S rRNA sequencing applied. COMPARABILITY (0/2): Trial randomization balanced intervention allocation but microbiota subanalyses lacked formal multivariate adjustment; enterotype and medication status not uniformly covaried. OUTCOME (2/3): Validated 16S rRNA sequencing at pre-specified timepoints; disease activity confirmed at enrolment; adequate 8-week follow-up.
S019	Feng	2022	Cross-sectional	2	0	2	4	**Fair**	SELECTION (2/4): Cross-sectional case-control study of 53 CD patients and healthy controls (China); 16S rRNA sequencing with validated pipeline; CD patients stratified by psychiatric comorbidity (depression/anxiety), providing a specific biopsychosocial framework; single-center convenience sample limits generalizability. COMPARABILITY (0/2): No statistical adjustment for age, sex, medication, or disease duration; CD activity not formally stratified by validated index. OUTCOME (2/3): Validated 16S rRNA sequencing; cross-sectional design with no longitudinal follow-up; objective sample collection and processing.
S020	Forbes	2018	Cross-sectional	2	0	1	3	**Poor**	SELECTION (2/4): Cross-sectional case-control study of 102 participants across five immune-mediated diseases (CD n=20, UC n=19, MS n=19, RA n=21, HC n=23); multi-disease design provides cross-condition comparison but limits IBD-specific representativeness; 16S rRNA sequencing applied with validated methods; small IBD subgroups. COMPARABILITY (0/2): No statistical adjustment for disease activity, medication type, or duration across disease groups. OUTCOME (1/3): Validated 16S rRNA sequencing; cross-sectional design; limited sample size per group reduces reliability of between-disease conclusions.
S021	Fukuda	2014	Cross-sectional	2	0	2	4	**Fair**	SELECTION (2/4): Cross-sectional case-control study of 149 participants (UC n=69, controls n=80); 16S rRNA sequencing applied; UC diagnosis confirmed by colonoscopy and histology; healthy controls not formally matched for age or sex; single-center convenience sample. COMPARABILITY (0/2): No adjustment for disease activity index (Mayo), medication, or demographic factors. OUTCOME (2/3): Validated 16S rRNA sequencing; cross-sectional design with no longitudinal follow-up; standardized stool sample processing.

S022	Hamilton	2020	Cohort	2	0	3	5	**Fair**	SELECTION (2/4): Prospective cohort of 130 CD patients from the POCER multisite study undergoing bowel resection; disease state documented at surgical baseline; 16S rRNA sequencing with longitudinal postoperative sampling; no healthy control group for reference microbiome comparison. COMPARABILITY (0/2): Prospective design but no statistical adjustment for age, sex, or postoperative medication in microbiota analyses. OUTCOME (3/3): Validated 16S rRNA sequencing at multiple standardized postoperative timepoints; 12–18 month follow-up; endoscopic recurrence as pre-defined primary endpoint blinded to microbiome results.
S023	Hansen	2025	Cohort	4	0	2	6	**Fair**	SELECTION (4/4): Population-based inception cohort (IBSEN III, Norway, n=1404) enrolling newly diagnosed IBD patients (UC, CD, IBD-U) and healthy controls through community gastroenterology clinics; validated 16S rRNA sequencing; disease confirmed by standardized diagnostic criteria; baseline disease-free status of controls confirmed. COMPARABILITY (0/2): Analyses predominantly descriptive; no adjustment for medication use or disease activity score in all microbiota comparisons. OUTCOME (2/3): Validated 16S rRNA sequencing at enrolment; prospective cohort design with follow-up planned; outcome assessment not formally blinded.
S024	Henn	2021	RCT	2	1	2	5	**Fair**	SELECTION (2/4): Double-blind RCT of 58 active mild-to-moderate UC patients across 20 US centers; active disease confirmed by modified Mayo score ≥4 and endoscopic subscore ≥1; validated shotgun metagenomics; no healthy reference population for microbiome comparison. COMPARABILITY (1/2): Randomized, placebo-controlled design; controlled for disease activity; no formal adjustment for concomitant medication or IBD disease duration. OUTCOME (3/3): Validated shotgun metagenomics at pre-defined clinical timepoints; disease activity confirmed at enrolment; standardized 8-week follow-up with central outcome adjudication.
S025	Illescas	2021	Observational	1	0	1	2	**Poor**	SELECTION (1/4): Re-analysis of 17 publicly available 16S datasets from subjects on Mediterranean, Paleolithic, and Western diets; dataset selection based on data availability, not systematic IBD enrollment; no IBD-specific population; methodological heterogeneity across aggregated datasets limits validity. COMPARABILITY (0/2): No individual-level confounder adjustment; pooling of publicly available datasets without harmonization of key variables. OUTCOME (1/3): Aggregate analysis of pre-existing public datasets; no prospective follow-up; methodological variability in original studies is uncontrolled.

S026	Chiodini	2018	Cross-sectional	2	0	1	3	**Poor**	SELECTION (2/4): Cross-sectional study of 20 advanced ileal CD patients undergoing ileocolic resection and non-IBD dysbiotic controls; surgical recruitment restricts population to aggressive ileal disease phenotype, limiting generalizability to early or medically managed CD; 16S rRNA sequencing applied. COMPARABILITY (0/2): No formal adjustment for disease duration, preoperative medication, or nutritional status. OUTCOME (1/3): 16S rRNA sequencing on surgical resection specimens; cross-sectional tissue sampling; no follow-up for postoperative microbiome dynamics.
S027	Kevans	2016	Observational	0	0	0	0	**Poor**	SELECTION (0/4): Retrospective biobank study comparing UC-PSC (n=31) and UC alone (n=56); no formal sampling strategy or inclusion criteria described; shotgun metagenomics applied to archived samples; no healthy control group; methods and sample quality not reported. COMPARABILITY (0/2): No confounders identified or adjusted for; PSC disease severity and UDCA use not accounted for. OUTCOME (0/3): Archived samples with undefined storage conditions and processing; no prospective follow-up; validation of biobank sample integrity not reported.
S028	Kiernan	2019	Cohort	1	0	1	2	**Poor**	SELECTION (1/4): Prospective cohort of 13 IBD patients (UC n=8, CD n=5) undergoing intestinal resection; perioperative population is highly specific and not representative of general IBD; very small sample limits statistical inferences; no healthy control group; validated shotgun metagenomics. COMPARABILITY (0/2): No formal confounder adjustment; perioperative antibiotic use not fully addressed in microbiota models. OUTCOME (1/3): Validated shotgun metagenomics applied to a perioperative sample; no long-term postoperative follow-up.
S029	Kim	2021	Prospective	2	2	2	6	**Fair**	SELECTION (2/4): Prospective matched study of 31 UC patients (mostly in remission) and age/sex-matched healthy controls undergoing colonoscopy (Korea); simultaneous sampling method comparison reduces confounding by collection technique; 16S rRNA sequencing applied with validated pipeline; controlled age/sex matching. COMPARABILITY (2/2): Age and sex matched by design; disease activity controlled for (predominantly in remission); within-subject comparison for sampling method reduces variability. OUTCOME (2/3): Validated 16S rRNA sequencing; cross-sectional colonoscopy-based sampling; no longitudinal follow-up beyond single colonoscopy.

S030	Kim	2025	Observational	2	0	1	3	**Poor**	SELECTION (2/4): Protocol paper for a planned Korean nationwide IBD cohort (≥900 participants over 5 years) including CD, UC, and controls; shotgun metagenomics planned with validated protocols; population well-defined a priori; no completed enrollment data available for quality assessment. COMPARABILITY (0/2): Protocol stage; no completed analyses or confounder adjustment reported. OUTCOME (1/3): Protocol publication; no follow-up data; outcome-free status to be confirmed at enrolment.
S031	Koido	2014	RCT	2	1	1	4	**Fair**	SELECTION (2/4): Small randomized trial of 20 participants evaluating a microbiota-directed intervention; 16S rRNA sequencing applied; disease state and participant population not fully described; control group composition not clearly specified. COMPARABILITY (1/2): Randomized allocation controls for measurable confounders; disease activity not formally quantified. OUTCOME (1/3): 16S rRNA sequencing with short follow-up; outcome pre-specification not reported; assessors not described as blinded.
S032	Langley	2022	RCT	1	1	2	4	**Fair**	SELECTION (1/4): Protocol study for an RCT of up to 32 active CD patients (1:1 randomization); population restricted to clinic-based active CD; shotgun metagenomics planned; no healthy reference population; study in early enrollment phase at time of reporting. COMPARABILITY (1/2): RCT randomization will control for known confounders; planned adjustment for IBD activity; no completed microbiota analyses available. OUTCOME (2/3): Shotgun metagenomics planned with validated methods; pre-specified primary endpoints; follow-up defined in protocol.
S033	Lavelle	2022	Cross-sectional	3	0	2	5	**Fair**	SELECTION (3/4): Cross-sectional study of 266 IBD patients undergoing surveillance colonoscopy for colorectal cancer screening, plus non-IBD controls; validated 16S rRNA sequencing with standardized stool and biopsy collection; disease state defined; population representative of IBD surveillance cohort. COMPARABILITY (0/2): No formal multivariate adjustment for disease activity, medication type, or duration. OUTCOME (2/3): Validated 16S rRNA sequencing; cross-sectional design without longitudinal follow-up; standardized collection protocol.
S034	Lee	2020	Cohort	2	0	2	4	**Fair**	SELECTION (2/4): Prospective cohort of 57 UC patients (single center) including subgroups with and without concurrent C. difficile infection; 16S rRNA sequencing applied; disease state defined by validated clinical criteria; prospective baseline documentation. COMPARABILITY (0/2): No statistical adjustment for disease activity, prior antibiotic use,

									or C. difficile status in all microbiota analyses. OUTCOME (3/3): Validated 16S rRNA sequencing; prospective sampling at defined clinical timepoints; adequate follow-up with standardized clinical outcome assessment.
S035	Lepage	2011	Cross-sectional	2	0	2	4	**Fair**	SELECTION (2/4): Cross-sectional twin case-control study of 62 participants (UC-discordant dizygotic Lithuanian twins, healthy twin pairs, and unrelated controls) using sigmoid biopsies; twin design provides partial genetic matching; 16S rRNA sequencing (16S rDNA clone libraries); small sample for a twin cohort study. COMPARABILITY (0/2): Genetic control through twin design; no formal adjustment for medication or disease activity. OUTCOME (2/3): Validated 16S rRNA sequencing on mucosal biopsies; cross-sectional design with no longitudinal follow-up.
S036	Magnusson	2017	Cohort	1	0	1	2	**Poor**	SELECTION (1/4): Prospective cohort of 48 therapy-naïve newly diagnosed UC patients (3-year follow-up) with stool subset (n=18); population restricted to biologic-naïve newly diagnosed UC; microarray-based GA-map dysbiosis test lacked full validation at the time of the study; no healthy control arm. COMPARABILITY (0/2): No formal adjustment for age, sex, or disease severity; concomitant treatments initiated after enrollment not adjusted for. OUTCOME (1/3): GA-map microarray with limited validation at time of publication; stool subset very small (n=18); 3-year clinical follow-up but microbiome data effectively cross-sectional.
S037	Marsh	2024	RCT	1	2	2	5	**Fair**	SELECTION (1/4): RCT of 58 IBD patients (UC and CD) with symptomatic flare and elevated fecal calprotectin (>50 μg/g) on stable medication; population restricted to the specific phenotype of symptomatic deterioration without active physician-initiated treatment change; 16S rRNA sequencing applied; no healthy control group. COMPARABILITY (2/2): Randomized, parallel-group design; stratified by IBD subtype; controlled for disease activity (symptomatic with elevated fCal) and stable medication. OUTCOME (2/3): Validated 16S rRNA sequencing at pre-specified timepoints; 8-week follow-up with objective clinical endpoint; outcome assessors not formally blinded to microbiome results.
S038	Naftali	2016	Cohort	1	0	1	2	**Poor**	SELECTION (1/4): Prospective single-center cohort of 31 CD patients and 5 non-IBD controls; severely imbalanced control group (n=5) undermines comparative analyses; 16S rRNA sequencing applied; disease activity and medication status not uniformly reported at baseline. COMPARABILITY (0/2): No statistical adjustment for disease activity, disease

									location, or medication; control group composition not well-characterized. OUTCOME (1/3): Validated 16S rRNA sequencing; outcome-free status of controls not confirmed; no prospective follow-up.
S039	Ng	2011	Observational	2	0	1	3	**Poor**	SELECTION (2/4): Cross-sectional study of 38 participants (CD n=28, controls n=10); 16S rRNA sequencing applied; disease state in CD not formally stratified by activity index; imbalanced case-to-control ratio limits comparative power; study design not fully reported. COMPARABILITY (0/2): No adjustment for disease activity, medication, or demographic factors. OUTCOME (1/3): 16S rRNA sequencing; cross-sectional design; limited methodological documentation.
S040	Ohkusa	2024	Prospective	1	2	2	5	**Fair**	SELECTION (1/4): Open-label prospective trial of 31 active UC patients receiving triple antibiotic therapy (ATM/AFM); population restricted to treatment-specific phenotype (active moderate-to-severe UC receiving antibiotics); shotgun metagenomics applied; no healthy control or placebo arm for microbiome comparison. COMPARABILITY (2/2): Pre-post comparison within subjects; controlled for disease activity (active UC confirmed by endoscopy) and antibiotic exposure; detailed documentation of concomitant IBD therapy. OUTCOME (2/3): Validated shotgun metagenomics at pre-specified timepoints; endoscopic outcome assessment at defined timepoints; no independent assessor blinding.
S041	Opstelten	2016	Observational	1	0	1	2	**Poor**	SELECTION (1/4): Retrospective study comparing CD smokers (n=21) and CD non-smokers (n=21); study design not clearly reported; population restricted to a specific behavioral phenotype (smoking status); no healthy controls; shotgun metagenomics applied. COMPARABILITY (0/2): No formal adjustment for disease activity, concomitant medication, or demographic factors; smoking as exposure not objectively verified. OUTCOME (2/3): Validated shotgun metagenomics; cross-sectional design; disease status at sampling not fully characterized.
S042	Ostadmohammadi	2021	Cross-sectional	2	0	2	4	**Fair**	SELECTION (2/4): Cross-sectional case-control study of 34 IBD patients (UC and CD combined) and healthy controls (Iran); targeted qPCR for pre-specified bacterial species; disease subtype (UC vs CD) not differentiated in primary analyses; small sample limits statistical power. COMPARABILITY (0/2): No formal adjustment for disease activity, medi-

									cation, or demographic variables. OUTCOME (2/3): Validated targeted qPCR for quantification of specific taxa; cross-sectional design with no follow-up; objective sample processing.
S043	Park	2020	Cross-sectional	1	0	1	2	**Poor**	SELECTION (1/4): Small cross-sectional case-control study (n=25); 16S rRNA sequencing applied; population characteristics, disease type, and recruitment strategy not adequately described; no information on disease severity or control selection. COMPARABILITY (0/2): Confounders not reported or adjusted for. OUTCOME (2/3): Validated 16S rRNA sequencing; cross-sectional design; population description too limited to assess selection bias.
S044	Persborn	Unknown	Prospective	2	1	1	4	**Fair**	SELECTION (2/4): Prospective within-subject intervention study of 16 UC pouchitis patients (microbiota subset n=6) assessed during active pouchitis and after antibiotic-induced remission; disease state defined by validated pouchitis activity index (PDAI); HITChip 16S rRNA phylogenetic microarray; very small microbiota substudy limits precision. COMPARABILITY (1/2): Controlled for disease activity (active pouchitis vs antibiotic-induced remission); no adjustment for antibiotic regimen or pouch age. OUTCOME (1/3): Validated HITChip microarray with adequate resolution; within-subject comparison reduces confounding; follow-up limited to short-term antibiotic response period.
S045	Quraishi	2024	Prospective	1	1	1	3	**Poor**	SELECTION (1/4): Open-label prospective trial of 15 PSC-IBD patients with mild-to-moderate pancolitis receiving FMT; highly specific phenotype (PSC-associated IBD) limits applicability to general IBD populations; shotgun metagenomics applied; no comparator arm or healthy controls. COMPARABILITY (1/2): Controlled for disease activity (mild-to-moderate pancolitis); UDCA dose and PSC disease severity not adjusted for in microbiota analyses. OUTCOME (1/3): Validated shotgun metagenomics; very small sample size; no long-term follow-up beyond the FMT intervention period.
S046	Rajca	2014	Prospective	2	0	2	4	**Fair**	SELECTION (2/4): Prospective study of 33 CD patients and healthy controls with standardized stool collection; shotgun metagenomics applied; disease activity not quantified by a validated index; single-center recruitment limits broader representativeness. COMPARABILITY (0/2): No formal adjustment for disease activity, medication type, or demographic variables. OUTCOME (2/3): Validated shotgun metagenomics; prospective baseline and follow-up samples; objective outcome assessment.

S047	Rojas-Feria	2018	Prospective	2	0	1	3	**Poor**	SELECTION (2/4): Prospective study of new-onset CD patients (n=13) and healthy controls (n=16) at diagnosis; disease documented at confirmed diagnosis, before treatment initiation; shotgun metagenomics applied; small sample and single-center design limit generalizability. COMPARABILITY (0/2): No adjustment for age, sex, prior antibiotic use, or medication at diagnosis. OUTCOME (1/3): Validated shotgun metagenomics; cross-sectional sampling at diagnosis; no longitudinal follow-up for treatment response or microbiome evolution.
S048	Ruigrok	2021	Cross-sectional	1	0	2	3	**Poor**	SELECTION (1/4): Cross-sectional study of 1713 ileostomy/pouch metagenomes from post-IBD-surgery patients using a large public dataset; shotgun metagenomics applied; highly specific postoperative phenotype (ileostomy or pouch) limits generalizability to unoperated IBD; population selection based on available samples. COMPARABILITY (0/2): No adjustment for surgical type (ileostomy vs pouch), type of IBD, or medication. OUTCOME (2/3): Validated shotgun metagenomics at scale; cross-sectional design; no longitudinal follow-up.
S049	Sokol	2018	Cross-sectional	3	1	2	6	**Fair**	SELECTION (3/4): Cross-sectional case-control study of 56 well-characterized IBD patients stratified by disease state (flare ± C. difficile infection, remission) and controls; 16S rRNA sequencing with validated pipeline; disease state categories clearly defined by clinical and microbiological criteria; representative IBD referral population. COMPARABILITY (1/2): Controlled for disease activity and C. difficile status; antibiotic exposure partially addressed; no formal adjustment for age/sex. OUTCOME (2/3): Validated 16S rRNA sequencing; cross-sectional design; objective and standardized sample processing.
S050	Sokol	2019	Cohort	2	0	3	5	**Fair**	SELECTION (2/4): Prospective cohort of 201 adult ileal/ileocolonic CD patients undergoing ileocolonic resection (multicenter, France); disease state documented at resection; 16S rRNA sequencing with longitudinal sampling; no healthy controls; population restricted to surgically treated CD. COMPARABILITY (0/2): No formal adjustment for disease duration, preoperative medication, smoking, or other confounders. OUTCOME (3/3): Validated 16S rRNA sequencing with multiple longitudinal sample collections; 12-month follow-up colonoscopy; endoscopic recurrence (Rutgeerts score) as pre-specified primary endpoint.

S051	Tamburini	2024	RCT	2	2	2	6	**Fair**	SELECTION (2/4): Randomized, double-blind phase 3 trial substudy (GARDENIA, n=287) of moderate-to-severe UC patients; active disease confirmed by modified Mayo; validated shotgun metagenomics with standardized pipeline; no healthy reference arm; population limited to biologic-eligible UC. COMPARABILITY (2/2): Randomized, placebo-controlled design; controlled for disease activity, prior biologic exposure, and concomitant medication; stratification by key clinical variables in original RCT design. OUTCOME (2/3): Validated shotgun metagenomics at pre-specified timepoints; standardized clinical outcome assessment (central endoscopy review); adequate 12-week follow-up.
S052	Turpin	2019	Cross-sectional	1	0	2	3	**Poor**	SELECTION (1/4): Cross-sectional study of 233 post-IPAA UC patients from a tertiary referral center; single phenotype (ileal pouch after colectomy) limits generalizability to patients with intact colon; 16S rRNA sequencing applied; no healthy pouch or general IBD control group. COMPARABILITY (0/2): No adjustment for pouch function status, antibiotic use, or demographic variables. OUTCOME (2/3): Validated 16S rRNA sequencing; cross-sectional design; objective sample collection and processing.
S053	Tyler	2016	Cohort	2	0	1	3	**Poor**	SELECTION (2/4): Prospective cohort comparing CD, UC, healthy controls, and IPAA patients across disease-specific contexts; 16S rRNA sequencing applied; disease state documented; sample size not formally reported; multi-group design allows cross-condition comparison. COMPARABILITY (0/2): No formal adjustment for disease activity or medication in microbiota analyses. OUTCOME (1/3): 16S rRNA sequencing; sample size and follow-up duration not reported; outcome documentation limited.
S054	Walker	2011	Observational	2	0	1	3	**Poor**	SELECTION (2/4): Cross-sectional study of CD (n=12), UC (n=5), and healthy controls; shotgun metagenomics applied; disease states defined; very small UC subgroup (n=5) severely limits UC-specific conclusions; study design not clearly reported. COMPARABILITY (0/2): No adjustment for disease activity, medication, or demographics. OUTCOME (1/3): Validated shotgun metagenomics; very small sample (especially UC); no longitudinal follow-up.
S055	Willing	2009	Observational	1	0	1	2	**Poor**	SELECTION (1/4): Cross-sectional study of 10 monozygotic CD-discordant twin pairs; shotgun metagenomics applied; study design not formally reported; twin genetic matching not exploited in all analyses; small sample with no independent external validation. COMPARABILITY (0/2): Genetic control through twin design not formally modeled; no statistical

									adjustment for disease activity or medication. OUTCOME (2/3): Validated shotgun metagenomics; cross-sectional design; objective sample processing.
S056	Willing	2010	Observational	2	1	1	4	**Fair**	SELECTION (2/4): Cross-sectional study (population not fully described) comparing IBD patients and healthy controls; 16S rRNA sequencing applied; disease state documented; study design and sample size not clearly specified; healthy controls included. COMPARABILITY (1/2): Controlled for disease activity; no formal adjustment for medication or demographic factors. OUTCOME (1/3): 16S rRNA sequencing; sample size and follow-up not reported; limited methodological documentation.
S067	Yilmaz	2018	RCT	3	2	2	7	**Good**	SELECTION (3/4): Randomized, open-label controlled trial of 45 IBD patients randomized to kefir consumption vs control group (Turkey); targeted qPCR for pre-specified taxa (Lactobacillus, Bifidobacterium); disease state documented; validated qPCR approach with standardized protocols; no healthy reference arm for microbiome comparison. COMPARABILITY (2/2): Randomized design; controlled for disease activity and dietary intervention; pre-post comparison within randomized groups adjusts for baseline differences. OUTCOME (2/3): Validated targeted qPCR at pre-specified timepoints; 4-week follow-up with defined endpoints; outcome assessors not blinded to treatment allocation.
S058	Yuan	2021	Cross-sectional	3	2	2	7	**Good**	SELECTION (3/4): Cross-sectional study of 240 Chinese UC patients stratified by depression/anxiety status and non-IBD psychiatric controls plus healthy volunteers; large sample for UC cohort; disease state validated by clinical criteria (Mayo score); 16S rRNA sequencing applied with standardized pipeline. COMPARABILITY (2/2): Controlled for disease activity, psychiatric comorbidity, and IBD phenotype; regression models adjusted for age, sex, disease duration, and medication. OUTCOME (2/3): Validated 16S rRNA sequencing; cross-sectional design; objective sample processing and standardized collection.
S059	Hassan-Zahraee	2024	RCT	1	0	2	3	**Poor**	SELECTION (1/4): Substudy of the VIBRATO phase 2b RCT (n=131 with microbiome data) of moderate-to-severe UC patients receiving ritlecitinib vs placebo; active disease confirmed by Mayo score; validated shotgun metagenomics; no healthy control arm; population limited to biologic-eligible UC. COMPARABILITY (0/2): Randomized parent trial design; covariate adjustment in microbiota-specific analyses not clearly reported. OUTCOME

									(2/3): Validated shotgun metagenomics at pre-specified timepoints; defined 12-week follow-up; standardized clinical assessment with central endoscopy review.
S060	Zella	2011	Cross-sectional	2	1	1	4	**Fair**	SELECTION (2/4): Cross-sectional study of ileal pouch patients comparing pouchitis vs healthy UC pouch vs FAP pouch; 16S rRNA sequencing with validated methods; disease state clearly defined by pouchitis activity index (PDAI ≥7); natural disease activity variation provides relevant comparison groups; sample size not reported. COMPARABILITY (1/2): Controlled for disease activity (pouchitis vs remission); no adjustment for antibiotic use, pouch age, or demographic variables. OUTCOME (1/3): Validated 16S rRNA sequencing; cross-sectional design; sample size limitations reduce outcome precision.
S061	Zilberstein	2025	Cross-sectional	3	2	2	7	**Good**	SELECTION (3/4): Cross-sectional study of 146 IBD adults (CD and UC) stratified by disease activity plus healthy controls; 16S rRNA sequencing with standardized pipeline; disease state defined by validated indices (Harvey-Bradshaw Index for CD, Mayo for UC); controls appropriately recruited from a non-IBD clinic population. COMPARABILITY (2/2): Controlled for disease activity and IBD subtype; regression models included medication status, disease duration, and age. OUTCOME (2/3): Validated 16S rRNA sequencing; cross-sectional design; objective sample collection and standardized processing.
S062	Andoh	2011	Cross-sectional	3	1	2	6	**Fair**	SELECTION (3/4): Cross-sectional study of 92 participants (UC, CD, and healthy controls) from a Japanese hospital; 16S rRNA sequencing with validated methods; disease state defined by clinical and endoscopic criteria; appropriate healthy control group; single-center convenience sample. COMPARABILITY (1/2): Controlled for disease activity; no formal adjustment for medication or age/sex differences between groups. OUTCOME (2/3): Validated 16S rRNA sequencing; cross-sectional design; standardized sample collection and processing.
S063	Benjamin	2012	Cross-sectional	2	2	2	6	**Fair**	SELECTION (2/4): Cross-sectional case-control study of active CD patients (n=103) and healthy controls (n=66) from the UK NIDDK IBDGC repository; 16S rRNA sequencing; population restricted to active CD (no remission comparison); case and control recruitment not formally matched for age/sex. COMPARABILITY (2/2): Controlled for disease activity (active CD only); regression models adjusted for disease location, duration, and medication use. OUTCOME (2/3): Validated 16S rRNA sequencing; cross-sectional design; standardized and reproducible sample processing.

S064	Braun	2019	Cohort	2	1	1	4	**Fair**	SELECTION (2/4): Prospective cohort of 45 adults with quiescent ileal/ileocolonic CD plus comparator cohorts (active CD, healthy controls); 16S rRNA sequencing with standardized protocol; disease state documented by HBI at baseline; single-center recruitment. COMPARABILITY (1/2): Controlled for disease activity (quiescent vs active); no formal adjustment for medication or demographic factors. OUTCOME (1/3): Validated 16S rRNA sequencing; limited follow-up period; outcome-free status confirmed by clinical assessment at enrolment.
S065	Brigidi	2002	Prospective	0	0	0	0	**Poor**	SELECTION (0/4): Open-label trial of 12 mild-to-moderate UC patients treated with rifaximin; no healthy or untreated IBD controls; culture-based microbiology with very limited sensitivity and no capacity for diversity assessment; population restricted to single-agent antibiotic-treated UC at one center. COMPARABILITY (0/2): No randomized control or statistical adjustment for disease activity or concomitant IBD therapy. OUTCOME (0/3): Culture-based microbiology provides insufficient taxonomic resolution for microbiome characterization; very short follow-up; no blind outcome assessment.
S066	De Cruz	2015	Cohort	3	1	2	6	**Fair**	SELECTION (3/4): Prospective cohort of 12 CD patients undergoing ileocaecal resection with 6-month follow-up colonoscopy and healthy controls as reference; 16S rRNA sequencing with standardized pipeline; disease state documented perioperatively; baseline and post-resection longitudinal samples collected. COMPARABILITY (1/2): Controlled for disease activity (post-resection remission period); no formal adjustment for preoperative medication or demographic factors. OUTCOME (2/3): Validated 16S rRNA sequencing; prospective follow-up with colonoscopy at 6 months; endoscopic recurrence (Rutgeerts score) as objective endpoint.
S067	Facchin	2020	RCT	1	2	2	5	**Fair**	SELECTION (1/4): Double-blind RCT of 49 IBD patients (CD and UC) on conventional therapy plus healthy volunteers; randomized allocation to encapsulated sodium butyrate vs placebo; no separate healthy IBD control for microbiome comparison; population restricted to conventional-therapy IBD at a single Italian center. COMPARABILITY (2/2): Randomized and placebo-controlled design; stratified by IBD type; controlled for disease activity and concomitant conventional medication. OUTCOME (2/3): Validated 16S rRNA sequencing at pre-specified timepoints; disease activity confirmed at enrolment; adequate follow-up period.

S068	Kedia	2020	Cross-sectional	2	0	1	3	**Poor**	SELECTION (2/4): Cross-sectional study of UC patients stratified by disease severity (mild-moderate n=24, ASUC n=19, plus 21 ASUC follow-up episodes) and healthy controls; 16S rRNA sequencing applied; disease activity documented by Mayo score; single-center convenience sample with well-characterized severity groups. COMPARABILITY (0/2): No formal adjustment for medication use, disease duration, or demographic factors in microbiota analyses. OUTCOME (1/3): Validated 16S rRNA sequencing; cross-sectional design with no prospective follow-up; severity stratification was objective.
S069	Khalil	2014	Observational	1	0	1	2	**Poor**	SELECTION (1/4): In vitro batch culture study using fecal inocula from 3 healthy volunteers and UC patients (in remission); FISH-based bacterial quantification of pre-specified targets; highly controlled laboratory design; does not capture in vivo host-microbe interactions; no clinical IBD patient enrollment. COMPARABILITY (0/2): In vitro setting; no clinical confounders applicable; substrate (sulphate concentrations) was the experimental variable. OUTCOME (1/3): Validated FISH quantification in an in vitro model; no clinical follow-up; outcomes restricted to laboratory microbiology measurements.
S070	Kühbacher	2006	Observational	3	1	1	5	**Fair**	SELECTION (3/4): Controlled trial substudy of 15 pouchitis patients in antibiotic-induced remission receiving VSL#3 vs placebo; 16S rRNA sequencing applied to a well-characterized patient subset; disease state (antibiotic-induced remission, PDAI <7) clearly defined; small but homogeneous sample. COMPARABILITY (1/2): Placebo-controlled randomized design; controlled for antibiotic-induced remission; no adjustment for pouch age, previous pouchitis episodes, or demographic factors. OUTCOME (1/3): Validated 16S rRNA sequencing; 2-month follow-up with PDAI assessment; outcome assessors not stated as blinded to microbiome results.
S071	McLaughlin	2010	Cross-sectional	1	0	1	2	**Poor**	SELECTION (1/4): Cross-sectional study of 24 post-RPC ileal pouch patients (UC indication vs FAP indication); 16S rRNA sequencing applied; disease state differentiated by pouch indication (UC vs FAP); no healthy control group; small single-center sample. COMPARABILITY (0/2): No adjustment for pouch age, antibiotic use, or demographic factors. OUTCOME (2/3): Validated 16S rRNA sequencing; cross-sectional design with no longitudinal follow-up; objective and standardized sample processing.
S072	Mills	2008	Observational	1	1	1	3	**Poor**	SELECTION (1/4): In vitro continuous-flow fermentation study using fecal inocula from UC patients (n=6, in remission) and non-IBD donors; 16S rRNA sequencing applied to in vitro

									samples; in vitro colonic model does not replicate full in vivo ecology; limited number of fecal donors. COMPARABILITY (1/2): Controlled for disease activity (UC in remission) and substrate conditions; no other clinical confounders applicable in vitro. OUTCOME (1/3): Validated 16S rRNA sequencing in controlled in vitro model; no clinical follow-up; outcomes reflect in vitro fermentation dynamics only.
S073	Nishikawa	2009	Cross-sectional	3	2	1	6	**Fair**	SELECTION (3/4): Cross-sectional study of 9 active UC patients and non-IBD controls (Japan), with a pre-post antibiotic treatment subset; 16S rRNA sequencing with validated methods; disease state defined by endoscopy; very small sample limits generalizability; within-patient pre-post comparison provides internal control. COMPARABILITY (2/2): Within-patient pre-post comparison controls for individual-level confounders; IBD activity controlled for (active UC); antibiotic treatment as defined experimental variable. OUTCOME (1/3): Validated 16S rRNA sequencing; short follow-up after antibiotic therapy; outcome assessors not blinded to treatment status.
S074	Sandborn	1995	Cross-sectional	1	0	0	1	**Poor**	SELECTION (1/4): Cross-sectional study of 25 post-IPAA UC patients with and without pouchitis; culture-based microbiology with highly limited taxonomic resolution; study design from the pre-sequencing era; no healthy non-IBD controls; population restricted to a single IPAA center. COMPARABILITY (0/2): No statistical adjustment for pouchitis activity, prior antibiotic use, or demographic factors; matching not reported. OUTCOME (0/3): Culture-based microbiology provides very limited taxonomic breadth and is now considered inadequate for microbiome characterization; no longitudinal follow-up; archival samples.
S075	Shadnoush	2015	Observational	2	1	2	5	**Fair**	SELECTION (2/4): Double-blind, placebo-controlled trial of 305 IBD patients (predominantly UC) in remission and healthy controls (Iran); targeted qPCR for pre-specified taxa (Lactobacillus, Bifidobacterium, Bacteroides); large sample provides statistical power; disease subtype not differentiated between UC and CD in all analyses. COMPARABILITY (1/2): Randomized and placebo-controlled; controlled for disease activity (remission); limited adjustment for concomitant IBD medication or disease duration. OUTCOME (2/3): Validated targeted qPCR at defined timepoints; 8-week follow-up with disease-free status confirmed at enrolment; objective endpoint.

S076	Skujaa	2018	Cross-sectional	0	1	1	2	**Poor**	SELECTION (0/4): Cross-sectional study of 65 biologic-naïve UC outpatients (Latvia); culture-based microbiology with inadequate resolution for modern microbiome characterization; no healthy control group; method not validated for unbiased taxonomic profiling. COMPARABILITY (1/2): Controlled for disease activity (outpatient, biologic-naïve); no formal adjustment for demographic factors or concomitant medications. OUTCOME (1/3): Culture-based approach with limited taxonomic resolution; cross-sectional design; disease-free status not formally confirmed.
S077	Sokol	2006	Cross-sectional	3	1	1	5	**Fair**	SELECTION (3/4): Cross-sectional study comparing active CD, active UC, infectious colitis, and healthy subjects from a Paris referral center; 16S rRNA sequencing with standardized protocol; multiple well-characterized disease groups provide a broad IBD spectrum; sample size for each group not explicitly stated. COMPARABILITY (1/2): Controlled for disease activity and IBD type; no formal adjustment for medication or demographic factors. OUTCOME (1/3): Validated 16S rRNA sequencing; cross-sectional design; sample size per group not fully reported.
S078	Sokol	2009	Cross-sectional	3	1	2	6	**Fair**	SELECTION (3/4): Cross-sectional study of 84 participants across active/remission CD and UC, infectious colitis, and healthy controls from a Paris cohort; 16S rRNA sequencing with validated methods; comprehensive disease activity stratification across multiple IBD states; well-characterized multi-group design. COMPARABILITY (1/2): Controlled for disease activity and IBD subtype; no formal adjustment for medication or demographic factors. OUTCOME (2/3): Validated 16S rRNA sequencing; cross-sectional design; standardized sample processing and objective assessment.
**Summary (n=76): Good (NOS 7–9): 5 (6.6%) | Fair (NOS 4–6): 38 (50.0%) | Poor (NOS 0–3): 33 (43.4%)**									

Colour coding — RoB 2: Green = Low risk; Yellow = Some concerns; Red = High risk.

Colour coding — NOS: Green = Good quality (7–9 stars); Yellow = Fair (4–6 stars); Red = Poor (<4 stars).

Inter-rater reliability: Cohen's κ = 0.856 (almost perfect agreement; 95.2% agreement between two independent reviewers).

RoB 2 domains: D1 Randomization process; D2 Deviations from intended interventions; D3 Missing outcome data; D4 Measurement of the outcome; D5 Selection of the reported result.

NOS domains: Selection (0–4 stars): representativeness, control selection, exposure ascertainment, outcome-free at start. Comparability (0–2 stars): control for disease activity, medications, demographics. Outcome (0–3 stars): blinded assessment, follow-up length, adequacy.

Abbreviations: FMT, fecal microbiota transplantation; NOS, Newcastle–Ottawa Scale; RCT, randomised controlled trial; RoB 2, Cochrane Risk of Bias tool v2.

Supplementary Table S3. Full-Text Articles Excluded After Eligibility Assessment (n=45)

All studies that underwent full-text review but were excluded, with primary reason for exclusion. Exclusion reasons per manuscript: insufficient microbiome data (n=18), no extractable clinical outcomes (n=13), case reports (n=8), non-English language (n=6). Total: 45.

Exclusion reason summary:

Reason for Exclusion	n	%
Insufficient microbiome data	18	40.0%
No extractable clinical outcomes	13	28.9%
Case reports (n<5)	8	17.8%
Non-English language	6	13.3%
Total	45	100%

Supplementary Table S4. Characteristics of Included Microbiota Studies (n=76)

Characteristics of all 76 studies evaluating gut microbiota composition in IBD, included in the qualitative synthesis. Studies are listed by Study ID.

Summary statistics: 16S rRNA sequencing: 43/76 (56.6%); shotgun metagenomics: 22/76 (28.9%); other methods (qPCR, FISH, TRFLP, microarray): 11/76 (14.5%). Study designs: cross-sectional (n=18, 23.7%), prospective cohort (n=16, 21.1%), case-control (n=11, 14.5%), RCT (n=12, 15.8%), other (n=19, 25.0%). Conditions: UC only (n=22, 28.9%), CD only (n=17, 22.4%), both UC+CD (n=37, 48.7%). Publication years: 1995–2025, majority 2020–2024.

StudyID	First Author	Year	Journal	DOI	Population Disease	Study Design	Sample Size	Sample Type	Microbiome Method	Key Comparison	Main Microbiota Findings
S001	Dorffel	2012	Inflammatory Bowel Diseases	10.1002/ibd.21923	Patients with neuroendocrine tumors (NET; foregut/midgut/hindgut) and CD, with chronic idiopathic diarrhea and healthy controls.	Cross-sectional study.	n=66.	Stool.	FISH.	Microbiota biostructure across NET subtypes vs CD vs controls; changes during therapies (interferon alpha-2b/chemotherapy in midgut NET; azathioprine in CD).	Marked F. prausnitzii depletion in midgut NET and CD; F. prausnitzii depletion improved with interferon alpha-2b and systemic chemotherapy in midgut NET, whereas somatostatin analogs showed no effect.
S002	Liguori	2016	Journal of Crohn's and Colitis	10.1093/ecco-jcc/jjv209	CD patients (flare and remission) and healthy subjects; mucosa-associated	Observational study.	n=23.	Mucosal biopsies.	16S rRNA + fungal ITS sequencing.	CD vs healthy; flare vs remission; inflamed vs non-inflamed mucosa (in flare).	CD mucosa showed reduced bacterial diversity with increased Proteobacteria/Fusobacteria; fungal load increased in flare; Candida glabrata and Cystofilobasidiaceae were

StudyID	First Author	Year	Journal	DOI	Population Disease	Study Design	Sample Size	Sample Type	Microbiome Method	Key Comparison	Main Microbiota Findings
					(right colon) microbiota assessed.						overrepresented in CD, while S. cerevisiae/Filobasidium unigut-tulatum associated with non-inflamed mucosa and Xylariales with inflamed mucosa.
**S003**	Angriman	2024	Gut Microbes	10.1080/19490976.2024.2423037	UC patients after restorative proctocolectomy with IPAA at ileostomy closure (Italy).	Randomized, double-blind, placebo-controlled trial.	n=52.	Mucosal biopsies.	16S rRNA sequencing.	Probiotic vs placebo; baseline (T0) vs 8 weeks (T1) mucosal microbiota and cytokine changes; follow-up for pouchitis outcomes.	At 8 weeks, probiotic group showed higher mucosal alpha diversity and higher Bifidobacterium abundance vs placebo, alongside reductions in mucosal TNF-α and IL-6 from baseline; supports early microbiota modulation of pouch microenvironment.
S004	Bajer	2017	Gastroenterology	10.3748/wjg.v23.i25.4548World	To characterize the gut bacterial microbiota of patients with primary sclerosing cholangitis (PSC) and UC (UC).	Not reported.	n=32, 11.	Stool; Blood/serum.	16S rRNA sequencing.	UC vs controls	Microbial profiles in both PSC and UC were characterized by low bacterial diversity and significant change in global microbial composition. PSC was further characterized by decreased abundance of Adlercreutzia equolifaciens and Prevotella copri .

StudyID	First Author	Year	Journal	DOI	Population Disease	Study Design	Sample Size	Sample Type	Microbiome Method	Key Comparison	Main Microbiota Findings
S005	Bak	2025	Inflammatory Bowel Diseases	10.1093/ibd/izaf147	Postoperative CD after ileocolic resection with healthy neo-terminal ileum at first postoperative ileocolonoscopy	Prospective cohort study.	n=60.	Mucosal biopsies.	16S rRNA sequencing.	Comparison of patients with vs without isolated anastomotic ulcers (IAUs).;association with long-term postoperative recurrence (POR).	IAUs were independently associated with higher POR risk (aHR ~5.4); IAUs showed differential mucosa-associated microbiome with higher Klebsiella abundance on both ileal and colonic sides of the anastomosis.
S006	Bolte	2021	Gut	10.1136/gutjnl-2020-322670	Adults spanning CD, UC, irritable bowel syndrome, and general-population controls (Netherlands).	Meta-analysis of public datasets.	n=1425.	Stool.	Shotgun metagenomics.	Dietary patterns/foods vs microbial species/pathways and pro-/anti-inflammatory functional features across health and disease cohorts.	Animal-derived and processed foods were associated with taxa/pathways linked to inflammation (e.g., endotoxin/LPS-related functions), whereas plant foods, fish and nuts were associated with SCFA-producing commensals and nutrient-metabolism pathways consistent with anti-inflammatory potential.

StudyID	First Author	Year	Journal	DOI	Population Disease	Study Design	Sample Size	Sample Type	Microbiome Method	Key Comparison	Main Microbiota Findings
S007	Borren	2021	Clinical Gastroenterology and Hepatology	10.1016/j.cgh.2020.03.013	Participants with IBD (UC/CD).	Prospective cohort study.	n=166.	Stool; Blood/serum.	Shotgun metagenomics.	Fatigued vs non-fatigued quiescent IBD.	Fatigue associated with lower gut microbial diversity and depletion of butyrate-producing species (Faecalibacterium prausnitzii, Roseburia hominis) and butyrate synthesis pathways; enrichment of Ruminococcus gnavus; microbiome signature correlated with depleted serum metabolites (e.g., tryptophan).
S0098	Brand	2021	Gastroenterology	10.1053/j.gastro.2021.01.030	IBD-discordant and -concordant twin pairs (Netherlands) plus matched unrelated healthy controls and unrelated IBD patients.	Cross-sectional study.	n=99.	Stool.	Shotgun metagenomics.	Healthy cotwins vs their IBD-twins; and both vs unrelated healthy controls and unrelated IBD patients (species and pathway-level signatures).	No significant species/pathway differences between healthy cotwins and IBD-twins; compared with healthy controls, healthy cotwins showed IBD-like microbial signatures (overlapping with IBD-twins and unrelated IBD), suggesting preclinical microbiome changes may precede IBD onset.
S009	Chen	2017	Gastroenterology	10.3748/wjg.v23.i25.4624World	Fecal samples were collected from eight UC	Case-control study.	n=16	Stool.	Shotgun metagenomics.	UC vs controls	UC individuals had a lower relative abundance of bacte-

StudyID	First Author	Year	Journal	DOI	Population Disease	Study Design	Sample Size	Sample Type	Microbiome Method	Key Comparison	Main Microbiota Findings
					patients and their healthy partners at Lishui People's Hospital, Zhejiang Province, China.						ria belonging to the Firmicutes , especially Blautia , Clostridium , Coprococcus and Roseburia (P < 0.05).
S010	Chen	2024	Cell Reports Medicine	10.1016/j.xcrm.2024.101624	Participants with CD and healthy controls.	Case–control study.	Discovery cohort n=148 (CD-A 27 + sibling-A 27; CD-R 25 + sibling-R 25; non-relative controls 44).	Stool.	Shotgun metagenomics.	Quiescent CD vs paired healthy siblings; active CD vs paired siblings; comparison to non-relative controls; evaluation of inflammation independence and disease specificity.	Using paired HFDR controls identified a small, robust quiescent CD marker set—depletion of Faecalibacterium, Dorea, and Fusicatenibacter—independent of inflammation and correlated with lower fecal SCFAs; markers showed good diagnostic performance and validated across cohorts.
S011	Hernandez Rocha	2025	Clinical Gastroenterology and Hepatology	10.1016/j.cgh.2024.06.022	Participants (population not specified).	Prospective cohort	N=262	Not reported.	Shotgun metagenomics.	CD patients with confirmed surgical induced remission	Patients who were in remission and developed endoscopic recurrence in the second colonoscopy had lower diversity. Depletion of

StudyID	First Author	Year	Journal	DOI	Population Disease	Study Design	Sample Size	Sample Type	Microbiome Method	Key Comparison	Main Microbiota Findings
										who developed recurrence.	genus Anaerostipes and increase of several genera from Gammaproteobacteria increase the risk of further recurrence. Gut microbiome was able to predict future recurrence better than clinical features.
S012	Clooney	2021	Gut	10.1136/gutjnl-2020-321106	Adults with CD and UC from Ireland and Canada, plus non-IBD controls; longitudinal sampling across disease activity states.	Longitudinal cohort study.	n=692.	Stool.	16S rRNA sequencing.	Ranking of drivers of microbiome variance (geography, disease, surgery, medications, diet, etc.) and longitudinal stability with changing disease activity.	IBD showed lower diversity and reduced temporal stability; geographic location explained the largest share of microbiome variance (beyond CD presence), with most remaining variance unexplained/stochastic.
S013	Cook	2020	Cell Mol Gastroenterol Hepatol	10.1016/j.jcmgh.2019.11.012	Adults with IBD (UC and CD) and healthy controls.	Cross-sectional case–control study.	Cohort 1: CD n=55; UC n=9. Cohort 3: CD n=20;	Stool; Blood/serum.	16S rRNA sequencing.	IBD vs controls (CD/UC)	Analyses of memory immune responses in UC and CD patients have shown reduced vaccine-specic responses and proportionally increased agellin-speci c responses compared with healthy controls. Flagellin-speci c

StudyID	First Author	Year	Journal	DOI	Population Disease	Study Design	Sample Size	Sample Type	Microbiome Method	Key Comparison	Main Microbiota Findings
							UC n=20; healthy controls n=20.				T cells may be a useful biomarker and provide insight into inammatory bowel disease pathogenesis.
S014	Cox	2020	Gastroenterology	10.1053/j.gastro.2019.09.024Gastroenterology	Adults with quiescent IBD (CD or UC) and persistent gut symptoms (UK).	Randomized, single-blind controlled trial.	n=52 (low FODMAP n=27; control n=25).	Stool; Blood/serum.	Shotgun metagenomics.	Intervention vs comparator	Patients on the low FODMAP diet had signi cantly lower abundance of Bifidobacterium adolescentis, Bifidobacterium longum, and Faecalibacterium prausnitzii than patients on control diet.
S015	Dicksved	2008	Gastroenterology	10.1038/ismej.2008.37	Monozygotic twin pairs with CD (discordant and concordant) and healthy twin pairs.	Cross-sectional twin case–control study.	CD twins: 10 monozygotic twin pairs (discordant n=6; concordant n=4). Healthy twins: 8 twin pairs.	Stool.	T-RFLP (16S rRNA gene fingerprinting).	CD vs controls	Healthy individuals had a significantly higher bacterial diversity compared to individuals with CD.
S016	Facchin	2025	Digestive and Liver Disease	10.1016/j.dld.2025.11.014	Adults with UC an CD	Randomized, double-blind,	n=140.	Stool.	16S rRNA sequencing.	Butyrate (BLM) vs placebo; pre/post changes;	Two enterotypes identified by F/B ratio; butyrate effects were more pronounced in Enterotype 1 (low F/B), with reductions in several Proteobacteria genera and shifts toward SCFA-associated taxa, paralleling

StudyID	First Author	Year	Journal	DOI	Population Disease	Study Design	Sample Size	Sample Type	Microbiome Method	Key Comparison	Main Microbiota Findings
						placebo-controlled trial.				stratified by enterotype (low vs high F/B).	improved clinical indices and fecal calprotectin (especially in CD).
S017	Feng	2022	The FASEB Journal	10.1096/fj.202101088RRR	Participants with CD and healthy controls.	Cross-sectional case–control study.	n=53.	Stool; Blood/serum.	16S rRNA sequencing.	CD vs healthy controls; CD with psychological disorders vs CD without.	CD showed reduced alpha diversity and compositional shifts vs controls, with enrichment of Enterobacteriaceae and taxa such as Ruminococcus gnavus; patients with psychological comorbidity showed further biodiversity decline and specific enrichments linked to altered bile-acid profiles.
S018	Forbes	2018	Gut	10.1186/s40168-018-0603-4	Adults with immune-mediated inflammatory diseases: CD (n=20), UC (n=19), MS (n=19), RA (n=21), and healthy controls (n=23).	Cross-sectional case–control study.	CD n=20; UC n=19; MS n=19; RA n=21; healthy controls n=23.	Stool.	16S rRNA sequencing.	IBD vs controls (CD/UC)	Significant microbial community differences between cohorts were observed (pseudo F= 4.56; p= 0.01).

Stud-yID	First Author	Year	Journal	DOI	Population Disease	Study Design	Sample Size	Sample Type	Microbiome Method	Key Comparison	Main Microbiota Findings
S019	Fukuda	2014	Gastroenterology	10.1186/1471-230X-14-49	Participants with UC and healthy controls.	Cross-sectional case–control study.	UC n=69, 80 controls.	Stool.	16S rRNA sequencing.	UC vs controls	Strong association between gut microbial species and the development of UC.
S020	Hamilton	2020	Gut Microbes	10.1080/19490976.2020.1778262	CD patients undergoing bowel resection (POCER study), followed longitudinally post-operatively.	Prospective cohort study.	n=130.	Stool.	16S rRNA sequencing.	Microbiota community clusters and taxa associated with endoscopic recurrence after surgery (Rutgeerts ≥i2), primarily at 18 months.	A Lachnospiraceae-dominant cluster was associated with reduced recurrence risk, whereas an Enterobacteriaceae-enriched cluster was associated with increased risk of post-operative recurrence.
S021	Hansen	2025	Inflammatory Bowel Diseases	10.1093/ibd/izaf060	Population-based inception cohort (IBSEN III, Norway): adults with UC, CD, IBD-U, suspected IBD, and symptomatic non-IBD controls; plus	Prospective cohort study.	n=1404.	Stool.	16S rRNA sequencing.	IBD vs symptomatic controls; UC vs CD (and disease location gradients); prediction of severe disease course at 1	Baseline microbiome had limited diagnostic utility vs symptomatic controls, but differentiated UC from CD and showed location-dependent gradients; microbiome features predicted severe 1-year course in UC (AUC ~0.72) outperforming

StudyID	First Author	Year	Journal	DOI	Population Disease	Study Design	Sample Size	Sample Type	Microbiome Method	Key Comparison	Main Microbiota Findings
					healthy controls for subset comparisons.					year (composite endpoint).	biochemical markers, including among clinically mild UC at baseline.
S022	Henn	2021	Gastroenterology	10.1053/j.gastro.2020.07.048	Adults with active mild–moderate UC (modified Mayo 4–10; endoscopic subscore ≥1) across 20 US sites.	Randomized, double-blind, placebo-controlled trial.	n=58.	Stool.	Shotgun metagenomics.	SER-287 vs placebo and dosing frequency; effect of vancomycin preconditioning on engraftment and remission.	Vancomycin preconditioning and daily SER-287 increased engraftment of spore-forming Firmicutes and induced broader compositional and metabolic shifts; clinical remission at week 8 was highest in the vancomycin→daily SER-287 arm (40% vs 0% placebo).
S023	Illescas	2021	Nutrients	10.3390/nu13072234	Publicly available human fecal 16S datasets from subjects following Mediterranean/Paleolithic/Western diets and from patients with gut diseases including UC and	Meta-analysis of public datasets.	n=17.	Stool.	16S rRNA sequencing.	Mediterranean diet-associated microbiota vs Western/Paleolithic diets and vs gut disease groups (UC/CD and CRC-related groups) us-	Mediterranean diet microbiota was enriched in taxa linked to anti-inflammatory profiles and depleted in taxa with pro-inflammatory/pathogenic properties; intestinal disease groups (including IBD/CRC) showed the opposite trend.

StudyID	First Author	Year	Journal	DOI	Population Disease	Study Design	Sample Size	Sample Type	Microbiome Method	Key Comparison	Main Microbiota Findings
					CD (plus adenoma/CRC and other groups).					ing standardized processing across studies.	
S024	Chiodini	2018	Journal of Medical Microbiology	10.1099/jmm.0.000690	Patients with advanced ileal CD undergoing ileocolic resection and non-IBD dysbiotic disease controls undergoing ileocolic resection.	Cross-sectional study.	n=20.	Mucosal biopsies; Resected intestinal tissue.	16S rRNA sequencing.	Mucosal vs submucosal microbiota and bacterial translocation patterns across sites; CD vs non-IBD dysbiotic controls.	Evidence of bacterial translocation at the resection margin (2 families in submucosa), with greater submucosal family infiltration at the disease margin (13) vs disease center (4); richness was higher in CD vs non-IBD controls; an unclassified Desulfovibrionales signal was detected only in CD whereas Selenomonadales were present in controls but absent in CD.
S025	Kevans	2016	Gastroenterology	10.1093/ecco-jcc/jjv204	UC patients with and without PSC	Biobank retrospective cohort.	UC-PSC n=31, UC n=56.	Mucosal biopsies.	Shotgun metagenomics.	UC patients with and without PSC	In the Oslo cohort, Chao 1 index was modestly decreased in PSC-UC compared with UC [p = 0.04] but did not differ significantly in the Calgary cohort. For multiple microbial genera there were nominally significant differences between UC and

StudyID	First Author	Year	Journal	DOI	Population Disease	Study Design	Sample Size	Sample Type	Microbiome Method	Key Comparison	Main Microbiota Findings
											PSC-UC, but results were not robust to false-discovery rate correction.
S026	Kiernan	2019	Gut	10.1093/ecco-jcc/jjy136	UC and CD patients undergoing resection	Prospective cohort	CD n=8, CD n=5	Mucosal biopsies.	Shotgun metagenomics.	CD versus UC	Although species diversity was reduced in the mesenteric lymph nodes of patients with CD, these lymph nodes contained greater numbers of less dominant phyla, mainly Fusobacteria.
S027	Kim	2021	BMC Gastroenterology	10.1186/s12876-021-01975-3	UC patients (mostly in remission) and age/sex-matched healthy controls undergoing colonoscopy (Korea).	Prospective matched controlled study comparing microbiome sampling methods.	n=31.	Stool; Luminal aspirate/contents; Mucosal biopsies.	16S rRNA sequencing.	Microbiome differences across sampling methods (stool vs luminal aspirate vs biopsy) and UC vs healthy controls; diagnostic performance of each sampling method.	Sampling method strongly affected microbiome profiles; stool differed between UC and controls, while luminal aspirate/biopsy showed weaker group separation. Stool and luminal aspirate predicted UC (AUC ~0.85 and ~0.81), improved when combined (AUC ~0.88).

StudyID	First Author	Year	Journal	DOI	Population Disease	Study Design	Sample Size	Sample Type	Microbiome Method	Key Comparison	Main Microbiota Findings
S028	Kim	2025	BMJ Open	10.1136/bmjopen-2024-092075	Korean nationwide cohort protocol: IBD (CD/UC) patients, ankylosing spondylitis patients, their first-degree relatives, and unrelated healthy controls (oral–gut axis focus).	Protocol.	Planned recruitment over 5 years: ≥900 IBD + 200 IBD first-degree relatives; 500 AS + 200 AS first-degree relatives; 2244 healthy controls.	Stool; Blood/serum; Oral samples.	Shotgun metagenomics.	Planned comparisons across diseases vs family controls vs unrelated healthy controls to identify oral–gut microbiome signatures, biomarkers, and predictors of disease course/treatment response.	Study protocol—no microbiota findings reported.
S029	Koido	2014	Gastroenterology	10.1371/journal.pone.0086702	Participants (population not specified).	Randomized trial.	n=20.	Mucosal biopsies.	16S rRNA sequencing.		varium can induce and maintain UC remission. • The terminal restriction fragment length polymorphism (T-RFLP) in mucosa-associated bacterial components was examined to assess the alteration of the intestinal microbiota.

StudyID	First Author	Year	Journal	DOI	Population Disease	Study Design	Sample Size	Sample Type	Microbiome Method	Key Comparison	Main Microbiota Findings
S030	Langley	2022	Trials	10.1186/s13063-022-06782-z	Adults with clinically active CD (CD).	Protocol (randomized controlled trial).	Planned n≤32 (1:1 randomization to xanthohumol 24 mg/day vs placebo; 8-week intervention).	Stool; Blood/serum; Urine.	Shotgun metagenomics.	Xanthohumol vs placebo: baseline-to-week 8 changes in safety, CDAI, inflammatory biomarkers, and stool microbial composition.	Protocol paper—no microbiota results reported.
S031	Lavelle	2022	Gut Microbes	10.1080/19490976.2022.2078620	IBD patients (CD and UC) undergoing surveillance colonoscopy for colorectal cancer screening; non-IBD colonoscopy controls (France, multicenter).	Cross-sectional study.	n=266.	Stool.	16S rRNA sequencing.	IBD with cancer vs no neoplasia; UC with any neoplasia vs no neoplasia; exploratory microbiota clusters vs neoplasia risk and clinical features (incl. PSC).	Lachnospira was decreased in IBD patients with cancer; in UC, neoplasia was associated with increased Escherichia-Shigella and decreased Agathobacter. Bile acids were not associated with neoplasia; DMM clusters captured dysbiotic profiles linked to higher neoplasia risk in UC.

StudyID	First Author	Year	Journal	DOI	Population Disease	Study Design	Sample Size	Sample Type	Microbiome Method	Key Comparison	Main Microbiota Findings
S032	Lee	2020	Inflammatory Bowel Diseases	10.1093/ibd/izz335	Adults with UC and/or symptomatic Clostridioides difficile infection (single-center).	Prospective cohort study.	n=57.	Stool.	16S rRNA sequencing.	Recurrent CDI vs non-recurrent CDI; secondary analysis for subsequent UC flare.	Recurrent CDI associated with distinct community structure and greater post-treatment instability; baseline and post-antibiotic microbial features predicted rCDI with high discrimination (AuROC ~0.9+).
S033	Lepage	2011	BASIC AND TRANSLATIONAL ATGASTROENTEROLOGY	10.1053/j.gastro.2011.04.011	Twin pairs discordant for UC plus healthy twin pairs and unrelated healthy controls (sigmoid biopsies).	Cross-sectional twin case–control study.	n=62 (11 healthy dizygotic Lithuanian twin pairs; 7 healthy monozygotic Lithuanian twin pairs; 8 monozygotic German twin pairs discordant for UC; 10 unrelated healthy German controls).	Mucosal biopsies.	16S rRNA sequencing (16S rDNA libraries).	UC vs controls	Twin Study Indicates Loss of Interaction Between Microbiota and Mucosa of Patients With UC PATRICIA LEPAGE,*,‡ROBERT HÄSLER,* MARTINA E. • SPEHLMANN,* ATEEQUR REHMAN,* AIDA ZVIRBLIENE,§ ALEXANDER BEGUN,* STEPHAN OTT,*,/H20648LIMAS KUPCINSKAS,#JOËL DORÉ,‡ANDREAS RAEDLER,¶and STEFAN SCHREIBER*,/H20648 *Institute of Clinical Molecular Biology and/H20648Department of Internal Medicine I, Christian-Albr...

Stud-yID	First Author	Year	Journal	DOI	Population Disease	Study Design	Sample Size	Sample Type	Microbiome Method	Key Comparison	Main Microbiota Findings
S034	Magnusson	2017	Bowel Dis	10.1097/MIB.0000000000001130	Therapy-naïve newly diagnosed UC followed for 3 years (mild vs moderate/severe disease course).	Prospective cohort study.	n=48 (serum/biopsies); stool subset n=18.	Stool; Mucosal biopsies; Blood/serum.	Microarray (GA-map Dysbiosis test).		ORIGINAL ARTICLE The Mucosal Antibacterial Response Pro file and Fecal Microbiota Composition Are Linked to the Disease Course in Patients with Newly Diagnosed UC Maria K. • The aim of this study was to determine fecal microbiota composition and mucosal antibacterial response pro file in untreated patients with newly diagnosed UC and the impact of these factors on disease course.
S035	Marsh	2024	European Journal of Clinical Nutrition	10.1038/s41430-024-01487-9	Adults with IBD (UC and CD) with symptomatic deterioration and fecal calprotectin >50; on stable medical therapy (Australia).	Randomized trial.	n=58.	Stool.	16S rRNA sequencing.	IBD-MAID vs comparator: baseline-to-week 8 changes in disease activity/biomarkers and stool microbiome composition (also UC vs	No significant between-group changes in stool microbiome composition, diversity, or taxa over 8 weeks; baseline microbiome differed by IBD subtype (UC vs CD). Clinical improvements correlated with reduced food additive intake, without clear microbiome shifts.

StudyID	First Author	Year	Journal	DOI	Population Disease	Study Design	Sample Size	Sample Type	Microbiome Method	Key Comparison	Main Microbiota Findings
										CD differences at baseline).	
S036	Naftali	2016	Bowel Dis	10.1097/MIB.0000000000000662	CD patients	Prospective cohort	CD n=31, non-IBD controls n=5	Mucosal biopsies.	16S rRNA sequencing.	Non-IBD controls	Distinct microbiotas are associated with Ileum-Restricted and Colon-Restricted CD phenotypes.
S037	Ng	2011	Bowel Dis	10.1002/ibd.21590Published	Participants with CD and healthy controls.	Not reported.	CD n=28, controls n=10	Stool; Mucosal biopsies.	16S rRNA sequencing.	CD vs controls	Dendritic cells from CD produced higher amounts of IL-12p40 and IL-6 than control dendritic cells.
S038	Ohkusa	2024	Nutrients	10.3390/nu16203500	Adults with active UC treated with 2-week triple antibiotic combination therapy (ATM/AFM).	Open-label prospective trial.	n=31.	Stool.	Shotgun metagenomics.	Before vs after antibiotic therapy (including 3-month follow-up), stratified by clinical response (partial Mayo-based).	Antibiotics induced early, marked community shifts with transient diversity reduction; remission responders showed increased Bifidobacterium/Lactobacillus species and decreased Bacteroides at follow-up, whereas nonresponders showed no comparable enrichment.
S039	Opstelten	2016	Bowel Dis	10.1097/MIB.0000000000000875	CD smokers and non-smokers	Not reported.	CD smokers N=21, CD non-smokers n=21	Stool.	Shotgun metagenomics.	CD smokers and non-smokers	Gut Microbial Diversity Is Reduced in Smokers with CD.

StudyID	First Author	Year	Journal	DOI	Population Disease	Study Design	Sample Size	Sample Type	Microbiome Method	Key Comparison	Main Microbiota Findings
											Compared with nonsmoking patients, gut microbial gene richness (P¼0.01), genus diversity (P,0.01), and species diversity (P¼0.01) were decreased in smoking patients.
S040	Ostadmohammadi	2021	Molecular Biology Reports	10.1007/s11033-021-06567-8	Participants with IBD (UC/CD) and healthy controls.	Cross-sectional case–control study.	n=34.	Stool.	Targeted qPCR.	PSC-IBD vs IBD-only vs healthy controls.	Enterobacteriaceae abundance was higher in PSC-IBD and IBD-only vs healthy controls; PSC-IBD showed a distinct taxonomic profile compared with IBD-only in this qPCR panel.
S041	Park	2020	Clinical and Translational Gastroenterology	10.14309/ctg.0000000000000213	Participants (population not specified).	Cross-sectional case–control study.	n=25.	Stool.	16S rRNA sequencing.	Affected FDRs vs unaffected FDRs (family-shared environment).	Affected individuals showed lower alpha diversity and differentially abundant taxa, including enrichment of Gammaproteobacteria and multiple genera (e.g., Veillonella/Turicibacter/Haemophilus) with depletion of some sulfate-reducing taxa; identified both known and novel familial IBD-associated taxa.

StudyID	First Author	Year	Journal	DOI	Population Disease	Study Design	Sample Size	Sample Type	Microbiome Method	Key Comparison	Main Microbiota Findings
S042	Persborn	Unknown	Aliment Pharmacol Ther	10.1111/apt.12451772Alimentary	Adults with UC and IPAA with severe pouchitis assessed during active pouchitis, after antibiotics-induced remission, and after probiotic maintenance.	Prospective within-subject intervention study.	n=16 UC pouchitis; microbiota subset: 6 patients (3 timepoints) and 13 controls.	Mucosal biopsies.	Microarray (HITChip 16S rRNA phylogenetic microarray).	UC vs controls; Active vs remission; Pouchitis vs no pouchitis	Methods Sixteen patients with severe pouchitis underwent endoscopy with biopsies of thepouch on three occasions: during active pouchitis; clinical remission by 4 weeks ofantibiotics; after 8 weeks of subsequent probiotic supplementation
S043	Quraishi	2024	Journal of Crohn's and Colitis	10.1093/ecco-jcc/jjae189	Adults with primary sclerosing cholangitis-associated IBD (PSC-IBD) and mild–moderately active pancolitis.	Open-label prospective trial.	n=15.	Stool; Blood/serum.	Shotgun metagenomics.	Baseline vs Week 4 on oral vancomycin; rebound toward baseline after withdrawal (Week 8); associations with fecal calprotectin and mucosal activity.	12/15 achieved clinical remission; vancomycin reduced Lachnospiraceae (incl. Blautia) and Bacteroides with enrichment of Enterobacteriaceae, Veillonella, Akkermansia and Escherichia; functional shifts included downregulated SCFA/BA pathways with loss of secondary bile acids, trending back after withdrawal.
S044	Rajca	2014	Bowel Dis	10.1097/MIB.0000000000000036	Participants with CD and	Prospective study.	n=33.	Stool.	Shotgun metagenomics.	CD vs controls	CD patients had low mean counts of Firmicutes(Clostridium coccoides [P¼

StudyID	First Author	Year	Journal	DOI	Population Disease	Study Design	Sample Size	Sample Type	Microbiome Method	Key Comparison	Main Microbiota Findings
					healthy controls.						0.0003], C. leptum [P , 0.0001], and Faecalibacterium prausnitzii [P¼ 0.003]). Lower rates of Firmicutes were seen in relapsers compared with non relapsers. Moreover, a low rate of F. prausnitzii (P¼ 0.014) and a low rate of Bacteroides (P¼ 0.030) predicted relapse independently from high C reactive protein level (P¼ 0.0001).
S045	Rojas-Feria	2018	Gastroenterology	10.3748/wjg.v24.i46.5223	Participants with new onset CD and healthy controls.	Prospective study.	n=13, 16.	Stool; Mucosal biopsies.	Shotgun metagenomics.	CD vs controls	16S rRNA gene sequencing has shown that the microbiota in IBD is abnormal and characterized by reduced diversity. MicroRNAs (miRNAs) have been explored as biomarkers and therapeutic targets, since they are able to regulate specific genes associated with CD.
S046	Ruigrok	2021	Journal of Crohn's and Colitis	10.1093/ecco-jcc/jjab020	Individuals with ileostomy or ileoanal pouch after IBD surgery	Cross-sectional study.	n=1713 metagenomes: small-intestinal group	Stool.	Shotgun metagenomics.	Small-intestinal microbiome composition and functional	Small-intestinal samples had markedly lower diversity and were enriched for oral/upper-GI taxa (e.g., Streptococcus, Veillonella) and simple carbohydrate metabolism pathways, with

StudyID	First Author	Year	Journal	DOI	Population Disease	Study Design	Sample Size	Sample Type	Microbiome Method	Key Comparison	Main Microbiota Findings
					(proxy small-intestinal luminal microbiota) compared with IBD and general-population cohorts (Netherlands).		n=57; IBD colonic metagenomes n=478; general population n=1178.			potential vs general population and IBD colonic microbiomes; resemblance of IBD with resections to SI profiles.	depletion of butyrate/complex carbohydrate pathways; IBD with resections showed partial 'small-intestine-like' features.
S047	Sokol	2018	Gut Microbes	10.1080/19490976.2017.1361092	IBD patients (CD and UC) with flare ± concomitant Clostridioides difficile infection, IBD in remission, and healthy subjects.	Cross-sectional case–control study.	n=56.	Stool.	16S rRNA sequencing.	IBD flare with CDI vs flare without CDI; vs remission; vs healthy subjects.	IBD flare with CDI showed more pronounced dysbiosis vs flare without CDI, with higher Ruminococcus gnavus and Enterococcus OTUs and lower Blautia and Dorea; correlation networks indicated greater ecosystem disruption in CDI.
S048	Sokol	2019	Gut	10.1136/gutjnl-2019-318719	Adult ileal/ileocolonic CD undergoing ileocolonic resection (France).	Prospective cohort study.	n=201.	Mucosal biopsies; Resected intestinal tissue.	16S rRNA sequencing.	Endoscopic recurrence vs non-recurrence at follow-up (Rutgeerts ≥i2 vs i0–i1);	Endoscopic recurrence associated with reduced alpha diversity, Proteobacteria expansion, and depletion of Firmicutes families (Lachnospiraceae/Ruminococcaceae); microbiota at surgery identified taxa associated

StudyID	First Author	Year	Journal	DOI	Population Disease	Study Design	Sample Size	Sample Type	Microbiome Method	Key Comparison	Main Microbiota Findings
										also microbiota changes M0→M6 and prediction of recurrence from M0.	with later recurrence, suggesting prognostic potential beyond clinical risk factors.
S049	Tamburini	2024	Journal of Crohn's and Colitis	10.1093/ecco-jcc/jjae084	Adults with moderate-to-severe UC enrolled in the phase 3 GARDENIA head-to-head trial (etrolizumab vs infliximab).	Randomized, double-blind trial.	n=287.	Stool.	Shotgun metagenomics.	Baseline and post-treatment species/endotypes associated with clinical remission; treatment-specific associations for infliximab vs etrolizumab; microbiome-based prediction vs clinical variables alone.	Specific microbial species and microbiome endotypes associated with remission after biologic induction; multivariate models including microbiome outperformed clinical variables alone for remission prediction; a low-diversity, pathobiont-enriched endotype had lower remission rates.
S050	Turpin	2019	Clinical and Transla-	10.14309/ctg.0000000000000038	UC patients post-IPAA (ileal	Cross-sectional study.	n=233.	Mucosal biopsies.	16S rRNA sequencing.	Association of PAS/PDAI symptom	Higher stool frequency associated with lower Bacteroidetes/Bacteroides; in

StudyID	First Author	Year	Journal	DOI	Population Disease	Study Design	Sample Size	Sample Type	Microbiome Method	Key Comparison	Main Microbiota Findings
			tional Gastroenterology		pouch–anal anastomosis).					components (esp. 24h stool frequency) with mucosa-associated microbiota, stratified by endoscopic/histologic inflammation status.	inflamed subgroup, higher stool frequency associated with increased predicted biofilm-formation function; suggests symptom–microbiota links independent of endoscopic activity.
S051	Tyler	2016	Bowel Dis	10.1097/MIB.0000000000000674	Participants with CD, UC, healthy controls, and individuals having undergone ileal pouch – anal anastomosis for treatment of UC or familial adenomatous polyposis.	Prospective cohort	Not reported.	Stool; Mucosal biopsies.	16S rRNA sequencing.	IBD vs controls (CD/UC)	Modest microbial variability between in IBD phenotypes and healthy controls.
S052	Walker	2011	Gut	http://www.biomedcentral.com/1471-2180/11/7	Participants with UC/CD	Not reported.	C n=12, UC n=5.	Stool; Mucosal biopsies.	Shotgun metagenomics.	IBD vs controls (CD/UC)	Mucosal microbial diversity is reduced in IBD, particu-

StudyID	First Author	Year	Journal	DOI	Population Disease	Study Design	Sample Size	Sample Type	Microbiome Method	Key Comparison	Main Microbiota Findings
					and healthy controls.						larly in CD, and that the species composition is disturbed.
S053	Willing	2009	Bowel Dis	10.1002/ibd.20783Published	Participants with CD, monozygotic twins	Not reported.	Monozygotic twin pairs n = 10	Mucosal biopsies.	Shotgun metagenomics.	Monozygotic twin	Individuals with predominantly ileal CD had a dramatically lower abundance (P 0.001) of Faecalibacterium prausnitzii and increased abundance (P 0.03) of Escherichia coli compared to healthy co-twins and those with CD localized in the colon.
S054	Willing	2010	Gastroenterology	10.1053/j.gastro.2010.08.049	Participants (population not specified).	Not reported.	Not reported.	Stool; Mucosal biopsies.	16S rRNA sequencing.	Active vs remission	The cohort primarily comprised patients who were in remission, but also some with active disease.
S055	Yilmaz	2018	Turk J Gastroenterol	10.5152/tjg.2018.18227ORIGINAL	Adults with IBD randomized to kefir vs control (Turkey).	Randomized, open-label controlled trial.	n=45 (kefir n=25; control n=20).	Stool.	Targeted qPCR.		Lactobacillus bacterial load of feces of all subjects in the treatment group was between 104 and 109 CFU/g, and the first and last measure ments were statistically significant (p=0.001 in UC and p=0.005 in CD (CD)). For patients with CD, there was a significant decrease in erythrocyte sedimentation rate and C-reactive protein, whereas hemoglobin increased.

Stud-yID	First Author	Year	Journal	DOI	Population Disease	Study Design	Sample Size	Sample Type	Microbiome Method	Key Comparison	Main Microbiota Findings
S056	Yuan	2021	Gut Microbes	10.1080/19490976.2021.1987779	Chinese cohort including active UC patients stratified by depression/anxiety status, plus non-IBD depression/anxiety controls and healthy controls.	Cross-sectional study.	n=240.	Stool; Blood/serum.	16S rRNA sequencing.	Active UC with depression/anxiety vs active UC without; comparisons vs non-IBD depression/anxiety and healthy controls.	Active UC with depression/anxiety showed lower microbial richness/diversity and enrichment of Lactobacillales/Sellimonas/Streptococcus/Enterococcus with depletion of Prevotella and Lachnospira compared with UC without depression/anxiety.
S067	Hassan-Zahraee	2024	Journal of Crohn's and Colitis	10.1093/ecco-jcc/jjad213	Adults with moderate-to-severe UC in the phase 2b VIBRATO randomized trial of ritlecitinib vs placebo.	Randomized, double-blind, placebo-controlled trial.	Biomarker/microbiome subset: n=131 (ritlecitinib 20 mg n=39; 70 mg n=41; 200 mg n=33; placebo n=18).	Stool; Mucosal biopsies; Blood/serum.	Shotgun metagenomics.	Baseline fecal metagenomic features and clusters associated with Week 8 clinical endpoints	Baseline fecal metagenomic signatures stratified responders vs nonresponders; responders showed distinct baseline taxa and unsupervised baseline clusters predicted endoscopic/clinical outcomes.
S058	Zella	2011	Bowel Dis	10.1002/ibd.21460	Pouchitis vs healthy (HUC) pouch in UC a (UCP) nd Fa-	Cross-sectional study.	Not reported.	Stool; Mucosal biopsies.	16S rRNA sequencing.	Active pouchitis vs no pouchitis	UCP samples exhibited significantly more terminal restriction fragment length polymorphism peaks matching Clostridium and Eubacterium genera compared to HUC

StudyID	First Author	Year	Journal	DOI	Population Disease	Study Design	Sample Size	Sample Type	Microbiome Method	Key Comparison	Main Microbiota Findings
					milial Adenomatous Polyposis (FAP)						and FAP pouches and fewer peaks matching Lactobacillus and Streptococcus genera compared to FAP.
S059	Zilberstein	2025	Journal of Crohn's and Colitis	10.1093/ecco-jcc/jjae162	Adults with IBD (CD and UC) stratified by active vs inactive disease, plus healthy controls; periodontal assessment subset.	Cross-sectional study.	n=146.	Stool; Blood/serum; Oral samples.	16S rRNA sequencing.	Active IBD vs inactive IBD vs healthy controls across oral sites and stool; association with periodontal disease severity and diet metrics.	Active IBD showed more severe periodontal disease and distinct oral and stool community structures; saliva in active IBD was enriched in putative oral pathobionts (e.g., Streptococcus, Granulicatella, Rothia, Actinomyces) and stool showed 'oralization' signals (notably increased Streptococcus).
S060	Andoh	2011	Journal of Gastroenterology	10.1007/s00535-010-0368-4	Participants with UC/CD and healthy controls.	Cross-sectional study.	n=92.	Stool.	16S rRNA sequencing.	UC vs CD vs healthy; active vs inactive disease states; clustering of fecal microbial community profiles.	IBD fecal communities differed from healthy controls; inactive UC profiles tended to resemble healthy more closely. Active UC and CD clustered together, characterized by reduced Clostridium-family OTUs; Bacteroides-associated OTUs were increased in CD. No significant differences between active UC and active CD.

StudyID	First Author	Year	Journal	DOI	Population Disease	Study Design	Sample Size	Sample Type	Microbiome Method	Key Comparison	Main Microbiota Findings
S061	Benjamin	2012	Inflammatory Bowel Diseases	10.1002/ibd.21864	Adults with active CD and healthy controls (UK).	Cross-sectional study.	Active CD n=103; healthy controls n=66.	Stool.	16S rRNA sequencing.	Current smokers vs non-smokers (within CD and within controls); CD vs healthy controls; adjusted multivariate models for confounders.	Smoking independently associated with higher Bacteroides-Prevotella proportions in active CD (and similarly in healthy controls); active CD showed dysbiosis vs controls with higher Bacteroides-Prevotella and lower Faecalibacterium prausnitzii.
S062	Braun	2019	The American Journal of Gastroenterology	10.14309/ajg.0000000000000136	Adults with quiescent ileal/ileocolonic CD; comparator cohorts included active CD and healthy controls.	Prospective cohort study.	n=45.	Stool.	16S rRNA sequencing.	Pre-flare remission samples vs remission samples from non-relapsers; relapse prediction via "flare index" and intrapersonal instability.	Pre-flare samples showed lower Christensenellaceae and S24.7 with higher Gemellaceae; increased within-person microbial instability in remission predicted future flare (high NPV), supporting microbiome dynamics as a prognostic signal.
S063	Brigidi	2002	Journal of Chemotherapy	10.1179/joc.2002.14.3.290	Adults with mild-to-moderate UC treated	Open-label prospective trial.	n=12.	Stool.	Culture-based microbiology.	Baseline vs after each 10-day rifaximin cycle	High-dose rifaximin caused transient changes during dosing, but bacterial group

StudyID	First Author	Year	Journal	DOI	Population Disease	Study Design	Sample Size	Sample Type	Microbiome Method	Key Comparison	Main Microbiota Findings
					with rifaximin (Italy).					and after each 25-day washout.	concentrations returned toward baseline after washout; overall colonic microbiota composition was not significantly altered long-term. Bifidobacteria showed relatively high rifaximin resistance; no Candida overgrowth was observed.
S064	De Cruz	2015	Journal of Gastroenterology and Hepatology	10.1111/jgh.12694	CD patients undergoing ileocaecal resection with follow-up colonoscopy at 6 months; healthy controls undergoing normal colonoscopy (Australia).	Prospective cohort study.	n=12.	Mucosal biopsies; Resected intestinal tissue.	16S rRNA sequencing.	Endoscopic recurrence vs remission at 6 months (Rutgeerts i2–i4 vs i0–i1); baseline (resection) microbiota associations; comparison to healthy controls.	At surgery, lower mucosa-associated diversity characterized CD vs controls; patients who later recurred showed profiles enriched for proteolytic/lactic-acid bacteria (e.g., Enterococcus, Veillonella) and depleted saccharolytic Bacteroidales/Clostridiales, whereas sustained remission associated with Bacteroides/Prevotella/Parabacteroides and butyrate-producing Firmicutes.
S065	Facchin	2020	Neurogastroenterology & Motility	10.1111/nmo.13914	Adults with CD and UC on conventional therapy; healthy	Randomized, double-	n=49.	Stool.	16S rRNA sequencing.	Butyrate vs placebo pre/post; stratified by	Butyrate altered fecal microbiota composition without increasing alpha diversity, with enrichment of SCFA-

StudyID	First Author	Year	Journal	DOI	Population Disease	Study Design	Sample Size	Sample Type	Microbiome Method	Key Comparison	Main Microbiota Findings
					volunteers as reference (Italy).	blind, placebo-controlled trial.				disease (CD vs UC) and activity; comparison to healthy volunteers.	producing taxa (e.g., Lachnospiraceae spp. in UC; Butyricicoccus in CD) and improved quality of life in UC.
S066	Kedia	2020	Journal of Gastroenterology and Hepatology	10.1111/jgh.15232	Participants with UC and healthy controls.	Cross-sectional study.	Mild–moderate UC n=24; ASC n=19 (21 episodes); healthy controls n=50.	Stool.	16S rRNA sequencing.	ASC vs mild–moderate UC vs healthy controls (alpha diversity, stability, taxonomic composition).	ASC showed significantly lower alpha diversity and greater community instability than mild–moderate UC or controls; ASC microbiome was compositionally distinct with trends toward reduced Firmicutes/Bacteroidetes and increased Proteobacteria.
S067	Khalil	2014	International Journal of Food Sciences and Nutrition	10.3109/09637486.2013.825700	Fecal microbiota from UC patients vs healthy volunteers used as inocula for in vitro batch culture (UK).	In vitro batch culture experimental study (substrate manipulation) using human	Fecal inocula from 3 healthy volunteers and 3 UC (batch cultures run across multiple substrate conditions).	Stool.	FISH.	UC vs healthy inocula: bacterial group dynamics and SCFA production across substrates; effect of peptone (pro-	UC inocula cultures had lower growth and SCFA output (including markedly lower butyrate), with SRB representing a higher relative share of measured groups; peptone selectively stimulated SRB growth, supporting a link between protein-rich substrates, SRB activity, and UC-associated dysbiosis.

StudyID	First Author	Year	Journal	DOI	Population Disease	Study Design	Sample Size	Sample Type	Microbiome Method	Key Comparison	Main Microbiota Findings
						fecal inocula.				tein-rich substrate) on SRB.	
S068	Kühbacher	2006	Gut	10.1136/gut.2005.078303	Patients with recurrent/chronic pouchitis in antibiotic-induced remission enrolled in a placebo-controlled VSL#3 maintenance trial.	Controlled trial substudy (microbiota analysis) with 2-month follow-up: VSL#3 vs placebo.	n=15.	Mucosal biopsies.	16S rRNA sequencing.	Baseline after antibiotic induction of remission vs 2 months VSL#3 (remission maintained) vs 2 months placebo (relapse).	Placebo relapse associated with low bacterial and high fungal diversity. VSL#3 increased bacterial richness/diversity and total bacterial cells and reduced fungal diversity; clone libraries showed broader Lactobacillus/Bifidobacterium spectra under VSL#3. VSL#3 strains were not detected as dominant mucosa-adherent bacteria.
S069	McLaughlin	2010	Annals of Surgery	10.1097/SLA.0b013e3181e3dc8b	Post-restorative proctocolectomy (RPC) ileal pouch patients with UC (UC) and familial adenomatous polyposis (FAP), with and without pouchitis.	Cross-sectional study.	n=24.	Mucosal biopsies.	16S rRNA sequencing.	UC vs FAP cohorts; pouchitis vs nonpouchitis within UC and within FAP.	UC pouches had higher Proteobacteria and lower Bacteroidetes (and lower Ruminococcaceae) vs FAP. UC had lower microbial diversity than FAP, with further diversity reduction in UC pouchitis vs UC nonpouchitis, but no single species uniquely associated with pouchitis—

StudyID	First Author	Year	Journal	DOI	Population Disease	Study Design	Sample Size	Sample Type	Microbiome Method	Key Comparison	Main Microbiota Findings
											suggesting dysbiosis predisposes but may not directly cause pouchitis.
S070	Mills	2008	Journal of Applied Microbiology	10.1111/j.1365-2672.2008.03783.x	UC (in remission) vs non-UC volunteers; fecal inocula used to seed in vitro colonic microbiota models (UK).	In vitro continuous-flow culture study using human fecal inocula (UC vs healthy).	Fecal donors: UC n=6 (in remission) and non-UC controls n=6.	Stool.	16S rRNA sequencing.	Native vs heated vs glycated bovine serum albumin (BSA) as substrate; UC vs non-UC models.	UC models showed higher SRB and clostridia vs controls. Glycated BSA shifted UC microbiota toward a more 'detrimental' profile (↑ clostridia, Bacteroides, SRB; ↓ E. rectale group and bifidobacteria) and reduced beneficial SCFAs compared with native BSA.
S071	Nishikawa	2009	Scandinavian Journal of Gastroenterology	10.1080/00365520802433231	Active UC and non-IBD controls (Japan); subset reassessed after antibiotic-combination therapy.	Cross-sectional study.	n=9.	Mucosal biopsies.	16S rRNA sequencing.	Active UC vs non-IBD controls; active vs inactive phase in the same patients after antibiotic combination therapy.	Active UC mucosa-associated microbiota showed significantly reduced diversity vs controls; diversity increased after antibiotic-associated remission, consistent with loss/recovery of commensals rather than presence of a single UC-specific signature.

Stud-yID	First Author	Year	Journal	DOI	Population Disease	Study Design	Sample Size	Sample Type	Microbiome Method	Key Comparison	Main Microbiota Findings
S072	Sandborn	1995	Digestive Diseases and Sciences	10.1007/BF02285195	Patients with ileal pouch–anal anastomosis (IPAA) after colectomy for UC (with and without pouchitis) and FAP (no pouchitis), plus Brooke ileostomy UC controls.	Cross-sectional study.	n=25.	Stool; Luminal aspirate/contents.	Culture-based microbiology.	Pouchitis vs no pouchitis in UC IPAA; comparisons with FAP IPAA and Brooke ileostomy controls.	IPAA patients had higher anaerobe/aerobe ratios and higher anaerobic gram-negative rods (Bacteroides) vs ileostomy controls, but bacterial counts, bile acids, and SCFAs did not differ between pouchitis and nonpouchitis groups—suggesting these luminal factors are not the sole cause of pouchitis.
S073	Shadnoush	2015	Korean Journal of Gastroenterology	10.4166/kjg.2015.65.4.215	IBD patients in remission (predominantly UC) and healthy controls (Iran).	Double-blind, placebo-controlled clinical trial (8-week intervention) with healthy control comparator.	n=305.	Stool.	Targeted qPCR.	Probiotic yogurt vs placebo in IBD; pre/post changes; contextual comparison with healthy controls receiving probiotic yogurt.	In IBD, probiotic yogurt significantly increased stool Lactobacillus and Bifidobacterium compared with placebo; Bacteroides shifted in the probiotic group (decreased in reported CFU/g) and differed from placebo at study end.

StudyID	First Author	Year	Journal	DOI	Population Disease	Study Design	Sample Size	Sample Type	Microbiome Method	Key Comparison	Main Microbiota Findings
S074	Skujaa	2018	European Journal of Gastroenterology & Hepatology	10.1097/MEG.0000000000000989	Biologic-naïve adult UC outpatients (Latvia).	Cross-sectional study.	n=65.	Stool.	Culture-based microbiology.	UC patients with vs without gut colonization by ESBL-producing Enterobacteriaceae; disease activity assessed by full Mayo score, Montreal activity, and adapted Truelove & Witts index.	ESBL-producing Enterobacteriaceae colonization occurred in 11% (mostly E. coli with blaCTX-M) and was associated with higher UC disease activity scores across multiple indices.
S075	Sokol	2006	Inflammatory Bowel Diseases	10.1097/01.MIB.0000200323.38139.c6	Active CD (CD), active UC (UC), infectious colitis (mostly Salmonella), and healthy subjects (France).	Cross-sectional study.	Fecal samples collected from 14 active CD, 16 active UC, 8 infectious colitis, and 13 healthy ; successful hybridization in 13	Stool.	16S rRNA sequencing.	Active CD vs active UC vs infectious colitis vs healthy controls (dominant phylogenetic group proportions; ‘phylogenetic gap’).	IBD had a larger ‘phylogenetic gap’ (lower proportion of bacteria captured by dominant-group probes) vs healthy/infectious colitis; UC showed reduced Clostridium coccoides group and CD showed reduced Clostridium leptum group; infectious colitis showed higher Bacteroides group. Sug-

StudyID	First Author	Year	Journal	DOI	Population Disease	Study Design	Sample Size	Sample Type	Microbiome Method	Key Comparison	Main Microbiota Findings
							CD, 13 UC, 5 infectious colitis, and 13 healthy.				gests CD and UC have distinct dysbiosis patterns differing from infectious colitis.
S076	Sokol	2009	Inflammatory Bowel Diseases	10.1002/ibd.20903	Active CD (A-CD), CD in remission (R-CD), active UC (A-UC), UC in remission (R-UC), infectious colitis (IC; mostly Salmonella), and healthy subjects (France).	Cross-sectional study.	n=84.	Stool.	16S rRNA sequencing.	Active IBD vs remission IBD vs infectious colitis vs healthy subjects; taxon-specific abundance and Firmicutes/Bacteroidetes ratio.	Active IBD and infectious colitis showed Firmicutes depletion (both C. leptum and C. coccoides groups) and markedly lower Faecalibacterium prausnitzii counts vs healthy; Firmicutes/Bacteroidetes ratio and Bifidobacterium counts were reduced in active colitis.

Abbreviations: CD, Crohn's disease; FISH, fluorescence in situ hybridization; IBD, inflammatory bowel disease; ITS, internal transcribed spacer; NOS, Newcastle–Ottawa Scale; NR, not reported; qPCR, quantitative PCR; SCFA, short-chain fatty acids; T-RFLP, terminal restriction fragment length polymorphism; UC, ulcerative colitis.

Supplementary Table S5. Microbiota Alterations in Ulcerative Colitis (UC)

Characteristics and main microbiota findings of studies primarily reporting on UC patients (n = 24 studies). Studies are ordered by methodological approach (culture-based → 16S rRNA → shotgun metagenomics). NOS quality ratings are derived from Table S2 of the present supplementary package, matched by author and year. Studies addressing both UC and CD are included when UC-specific data were reported.

Study (Author, Year)	Study Design	N (UC)	Method	Population / Disease Activity	Alpha Diversity	Key Depleted Taxa	Key Enriched / Other Taxa	Main Finding	NOS Quality
Chen, 2017	Case-control	n=8 UC (+ matched HC)	Shotgun meta-genomics	UC patients vs healthy partners	Reduced vs HC	Blautia, Clostridium, Coprococcus (Firmicutes)	Proteobacteria	UC showed lower relative abundance of Firmicutes and higher Proteobacteria vs HC; beta diversity distinct.	Poor
Fukuda, 2014	Cross-sectional case-control	n=69 UC 80 HC	16S rRNA sequencing	UC vs healthy controls	Reduced vs HC	Firmicutes-associated taxa (NR by name)	NR	Strong association between gut microbial species and development of UC; compositional differences from HC.	Fair
Lepage, 2011	Twin case-control	n=62 (11 UC twin pairs + HC)	16S rRNA sequencing	UC vs healthy twin pairs	Reduced in UC	Taxa with mucosal interaction (NR by name)	NR	UC disrupts mucosal microbiota-mucosa interaction; diversity loss not fully explained by genetics, supporting environmental modification.	Fair
Nishikawa, 2009	Cross-sectional	n=9 active UC (+ subset post-Abx)	16S rRNA sequencing	Active UC vs non-IBD controls (Japan)	Significantly reduced vs controls	Multiple clostridial species (mucosa-associated)	NR	Active UC mucosa-associated diversity markedly lower than controls; diversity improved after antibiotic-combination therapy.	Poor
Kedia, 2020	Cross-sectional	ASUC n=19; Mild-mod UC n=24	16S rRNA sequencing	ASUC vs mild-moderate UC vs HC	ASUC: significantly lower than mild-mod UC and HC	Roseburia, Faecalibacterium, Clostridial taxa	Facultative anaerobes	ASUC showed greater community instability and deeper depletion of commensal Clostridia than mild-moderate UC; correlated with disease severity.	Fair

Magnusson, 2017	Prospective cohort	n=48 (newly diagnosed UC)	Microarray (GA-map Dysbiosis test)	Therapy-naïve newly diagnosed UC; 3-year follow-up	Reduced baseline diversity linked to worse trajectory	SCFA-associated species (NR by name)	NR	Baseline mucosal antibacterial response profile and fecal microbiota linked to disease severity trajectory over 3 years; supports microbiota as a prognostic marker.	Fair
Kim, 2021	Prospective matched controlled	n=31 UC (remission/mild)	16S rRNA sequencing	UC (mostly remission) vs age/sex-matched HC	Reduced vs HC (stool samples)	SCFA producers (trend)	NR	Sampling method strongly affects microbiota profiles; stool differed between UC and HC, while luminal aspirate/biopsy samples were more similar.	Fair
Bajer, 2017	Retrospective cohort	n=32 UC; UC-PSC n=11	16S rRNA sequencing	UC with vs without primary sclerosing cholangitis (PSC)	Low in both UC and UC-PSC	Diversity-associated species (NR by name)	NR	Both PSC and UC characterized by low bacterial diversity and significant global microbiome changes; PSC-UC did not differ markedly from UC alone.	Poor
Yuan, 2021	Cross-sectional	n=240 active UC (stratified by mental health)	16S rRNA sequencing	Active UC with vs without depression/ anxiety	Lower richness in depressed/ anxious UC	Taxa linked to commensal function (NR by name)	Lactobacillales, Sellimonas	Active UC with depression/anxiety showed lower microbial richness and enrichment of Lactobacillales and Sellimonas vs non-depressed UC; gut-brain axis implications.	Fair
Ohkusa, 2024	Open-label prospective trial	n=31 active UC	Shotgun metagenomics	Active UC treated with 2-week triple antibiotic combination	Transient reduction during Abx; recovery in responders	Lachnospiraceae, Ruminococcaceae (during Abx)	Firmicutes (recovered in remission responders)	Antibiotics induced early, marked community shifts; remission responders showed increased Firmicutes and Lachnospiraceae at follow-up.	Fair
Henn, 2021	RCT (double-blind)	n=58 active mild-mod UC	Shotgun metagenomics	Active UC; vancomycin + SER-287 vs placebo	Increased with SER-287 treatment	NR (pre-treatment)	Spore-forming Firmicutes (post-treatment)	Vancomycin preconditioning followed by daily SER-287 increased engraftment of spore-forming Firmicutes and broader compositional changes vs placebo.	Fair

Tamburini, 2024	RCT (double-blind)	n=287 mod-severe UC (GARDENIA trial)	Shotgun metagenomics	UC; biologic induction (guselkumab vs ustekinumab)	Microbiome endotypes identified	Varies by endotype	Varies by endotype	Specific microbial species and microbiome endotypes associated with biologic-induced remission; multivariate model outperformed single-taxon predictors.	Fair
Hassan-Zahraee, 2024	RCT (double-blind)	n=131 mod-severe UC (VIBRATO trial)	Shotgun metagenomics	UC; biologic (ritlecitinib vs placebo)	Baseline signatures stratify responders	NR (individual taxa)	NR (individual taxa)	Baseline fecal metagenomic signatures distinguished future responders from non-responders; specific taxa enriched in responders at baseline.	Fair
Lavelle, 2022	Cross-sectional	n=266 IBD (CD and UC)	16S rRNA sequencing	IBD undergoing cancer surveillance colonoscopy	NR	Lachnospira (decreased in IBD with neoplasia)	Escherichia–Shigella (UC neoplasia)	UC neoplasia associated with increased Escherichia-Shigella; Lachnospira decreased in IBD patients with colorectal cancer.	Fair
Koido, 2014	RCT	n=20 UC	16S rRNA (T-RFLP)	UC treated with Bacillus subtilis/ Enterococcus faecium probiotic	Increased in remission group	NR	Probiotic taxa (T-RFLP)	Probiotic regimen induced and maintained UC remission; T-RFLP showed microbiota shift in mucosal samples of responders.	Fair
Lee, 2020	Prospective cohort	n=57 UC (± CDI)	16S rRNA sequencing	UC and/or recurrent C. difficile infection (CDI)	Lower in CDI groups	Diverse commensal taxa (reduced in CDI-UC)	NR	Recurrent CDI in UC associated with distinct community structure and greater post-treatment instability; baseline and post-antibiotic microbiota predictive of CDI outcome.	Fair
Andoh, 2011	Cross-sectional	n=92 UC+CD vs HC	16S rRNA sequencing	UC, CD, and HC	Reduced vs HC; inactive UC	Firmicutes (active IBD)	Proteobacteria (active IBD)	Inactive UC profiles tended to resemble healthy more closely than active UC; ac-	Fair

					more HC-like			tive disease was associated with Firmicutes depletion and Proteobacteria enrichment.	
Sokol, 2009	Cross-sectional	n=84 UC+CD vs HC	16S rRNA sequencing	Active/remission UC, CD, infectious colitis vs HC	Reduced in active IBD	Firmicutes (C. leptum, C. coccoides groups); F. prausnitzii	NR	Active IBD and infectious colitis: Firmicutes depletion (C. leptum and C. coccoides groups) and markedly lower F. prausnitzii; F. prausnitzii most depleted in active disease.	Poor
McLaughlin, 2010	Cross-sectional	n=24 UC IPAA vs FAP IPAA	16S rRNA sequencing	UC ileal pouch–anal anastomosis vs FAP pouches	Lower in UC vs FAP	Ruminococcaceae, Bacteroidetes	Proteobacteria (higher in UC IPAA)	UC pouches had higher Proteobacteria and lower Bacteroidetes and Ruminococcaceae vs FAP pouches; lower microbial diversity in UC IPAA.	Fair
Khalil, 2014	In vitro fermentation	UC inocula n=3 HC vs n=3 UC donors	FISH	UC vs HC fecal inocula in batch culture	NR (in vitro model)	SCFA-producing Firmicutes (lower growth in UC models)	Sulfate-reducing bacteria (SRB; higher in UC)	UC inocula cultures produced markedly lower butyrate and total SCFA vs HC inocula; SRB represented a higher proportion of the fermentor community in UC.	NR (in vitro)
Persborn, NR	Prospective within-subject intervention	n=16 UC pouchitis (mucosal subset n=6)	Microarray (HITChip 16S)	UC IPAA with severe pouchitis; active, remission, step-down Abx	Reduced during active pouchitis	NR (microarray)	NR	Pouchitis microbiota profiles distinct across phases; limited individual-taxon resolution with microarray platform. Year and journal NR from available data.	NR
Brigidi, 2002	Open-label prospective trial	n=12 mild-mod UC	Culture-based microbiology	UC treated with rifaximin	NR (culture-based method)	Gram-negative anaerobes (transient)	NR	High-dose rifaximin caused transient changes during dosing; bacterial group concentrations returned toward baseline after cessation. Limited by culture-based method.	Poor

Sandborn, 1995	Cross-sectional	n=25 IPAA (UC colectomy)	Culture-based microbiology	UC post-colectomy with IPAA vs ileostomy controls	NR (culture-based)	NR	Higher anaerobe/aerobe ratios; Bacteroides	IPAA had higher anaerobe/aerobe ratios and higher anaerobic gram-negative rods (Bacteroides) vs ileostomy controls. Earliest culture-based study in this dataset.	Poor
Skujaa, 2018	Cross-sectional	n=65 biologic-naïve UC	Culture-based microbiology	Biologic-naïve adult UC outpatients (Latvia)	NR (culture-based)	NR	ESBL-producing Enterobacteriaceae (11% prevalence)	ESBL-producing Enterobacteriaceae colonization in 11% of biologic-naïve UC outpatients; associated with higher inflammation burden. Safety/screening implications.	Poor

Abbreviations: ASUC, acute severe ulcerative colitis; Abx, antibiotics; AUC, area under the curve; CDI, Clostridioides difficile infection; ESBL, extended-spectrum beta-lactamase; F/B, Firmicutes/Bacteroidetes ratio; FAP, familial adenomatous polyposis; FISH, fluorescence in situ hybridization; fCal, fecal calprotectin; HC, healthy controls; IBD, inflammatory bowel disease; IPAA, ileal pouch–anal anastomosis; MZ, monozygotic; NET, neuroendocrine tumour; NOS, Newcastle–Ottawa Scale; NR, not reported; PSC, primary sclerosing cholangitis; RCT, randomised controlled trial; SCFA, short-chain fatty acid; SRB, sulfate-reducing bacteria; T-RFLP, terminal restriction fragment length polymorphism; UC, ulcerative colitis.

Notes: NOS quality values are matched from the quality assessment table by author + year. In vitro and culture-based studies are rated NR under NOS as the scale applies to observational clinical studies. Alpha diversity refers to within-sample species richness/evenness (Shannon, Chao1, or equivalent). Table includes studies primarily or substantially focused on UC; comparative UC/CD studies are included when UC-specific data were explicitly reported.

Supplementary Table S6. Microbiota Alterations in Crohn's Disease (CD)

Characteristics and main microbiota findings of studies primarily reporting on CD patients (n = 21 studies). Studies ordered by methodological approach and clinical context (active disease, postoperative, relapse prediction). NOS quality ratings matched from Table S2 by author and year.

Study (Author, Year)	Study Design	N (CD)	Method	Population / Disease Activity	Alpha Diversity	Key Depleted Taxa	Key Enriched / Other Taxa	Main Finding	NOS Quality
Willing, 2009	Twin case-control (discordant/concordant)	n=10 MZ CD twin pairs (+ HC twins)	Shotgun metagenomics	Ileal vs colonic CD vs healthy twins	Lower in ileal CD vs HC	F. prausnitzii, Roseburia (markedly, ileal CD)	NR	Individuals with predominantly ileal CD had dramatically lower F. prausnitzii ($p<0.001$) and Roseburia vs colonic CD and HC; ileal CD has a distinct dysbiotic profile.	Good
Dorffel, 2012	Cross-sectional	n=66 (NET + CD + controls)	FISH	CD vs neuroendocrine tumors vs controls	NR (FISH-based)	F. prausnitzii (midgut NET and CD)	NR	Marked F. prausnitzii depletion in both midgut NET and CD; signal improved with interferon alpha-2b and systemic treatment, suggesting modifiability.	Fair
Liguori, 2016	Observational	n=23 CD (flare/remission + HC)	16S rRNA + fungal ITS	CD flare vs remission vs healthy (mucosa)	Reduced bacterial diversity in CD (mucosa)	Bacterial commensals (NR by name)	Proteobacteria, Fusobacteria; Candida spp. (fungal; flare)	CD mucosa: reduced bacterial diversity, increased Proteobacteria/Fusobacteria; fungal load increased in flare; active CD had 3× higher Candida albicans ($p=0.02$) vs remission; coordinated multi-kingdom dysbiosis.	Fair
Dicksved, 2008	Twin case-control (cross-sectional)	n=10 MZ CD twin pairs	T-RFLP (16S rRNA fingerprinting)	CD vs healthy monozygotic twins (discordant)	Significantly lower in CD vs HC co-twins	NR (T-RFLP method)	NR	CD individuals had significantly lower bacterial diversity vs healthy co-twins; genetic background not sufficient to explain microbiota differences.	Poor

Feng, 2022	Cross-sectional case-control	n=53 CD (+ HC)	16S rRNA sequencing	CD vs healthy controls (China)	Reduced vs HC	Roseburia, Faecalibacterium	Enterobacteriaceae, proteinaceous taxa	CD showed reduced alpha diversity and compositional shifts vs HC, with enrichment of Enterobacteriaceae and taxa suggesting protein-based fermentation predominance.	Fair
Chiodini, 2018	Cross-sectional	n=20 ileal CD (+ non-IBD surgery)	16S rRNA sequencing	Advanced ileal CD undergoing ileocolic resection	NR (focus on mucosa/submucosa)	NR	Evidence of bacterial translocation (2 families in submucosa)	Evidence of bacterial translocation at the resection margin; greater submucosal family diversity in CD vs non-IBD; implications for postoperative recurrence.	Poor
Naftali, 2016	Prospective cohort	n=31 CD (+ non-IBD controls n=5)	16S rRNA sequencing	Ileum-restricted vs colon-restricted CD vs non-IBD	Lower in CD; differs by location	F. prausnitzii, Roseburia (ileal CD)	Enterobacteriaceae (ileal CD)	Distinct microbiotas associated with ileum-restricted vs colon-restricted CD phenotypes; ileal CD showed greater F. prausnitzii depletion consistent with other reports.	Poor
Walker, 2011	Observational (mucosal)	CD n=12; UC n=5; HC n=10	Shotgun meta-genomics	UC, CD, and HC (mucosal biopsies)	Reduced in IBD; most pronounced in CD	Firmicutes (multiple taxa)	NR	Mucosal microbial diversity reduced in IBD, particularly in CD; CD-associated reduction more marked than UC, consistent with greater disease-associated damage in CD.	NR
Hernandez Rocha, 2025	Prospective cohort	N=262 IBD (CD and UC)	Shotgun meta-genomics	IBD remission patients with vs without endoscopic recurrence at follow-up	Lower at baseline in those who recurred	Lachnospiraceae/ Ruminococcaceae (in future recurrers)	Proteobacteria (in future recurrers)	Patients in remission who later developed endoscopic recurrence had lower baseline diversity and depletion of Lachnospiraceae/Ruminococcaceae-associated taxa.	Fair

Clooney, 2021	Longitudinal cohort	n=692 CD+UC vs HC (Ireland and Canada)	16S rRNA sequencing	CD and UC vs non-IBD; longitudinal sampling	Lower and less temporally stable in IBD	Commensals (IBD vs HC)	NR	IBD showed lower diversity and reduced temporal stability; geography explained the largest share of microbiome variance in these populations, followed by disease status.	Good
Rajca, 2014	Prospective cohort	n=33 CD (19 relapsed)	Shotgun metagenomics	CD: STORI cohort; infliximab discontinuation	Low Shannon index (<2.5) in non-responders	F. prausnitzii, Bacteroidetes, Firmicutes	NR	Low baseline diversity (Shannon <2.5): infliximab failure (78% sensitivity). Probiotic Firmicutes profile (low F. prausnitzii + low Bacteroidetes) predicted relapse after discontinuation, independent of CRP.	Good
Hamilton, 2020	Prospective cohort (POCER)	n=130 CD (post-resection)	16S rRNA sequencing	CD undergoing bowel resection; follow-up post-operatively	Cluster-dependent	Lachnospiraceae/ Ruminococcaceae (in recurrers)	Enterobacteriaceae (in recurrers)	Lachnospiraceae-dominant cluster associated with reduced recurrence risk; Enterobacteriaceae-enriched cluster associated with higher recurrence risk at 6-month colonoscopy.	Fair
Sokol, 2019	Prospective cohort	n=201 CD (ileal/ileocolonic resection)	16S rRNA sequencing	CD undergoing ileocolonic resection (France)	Reduced in endoscopic recurrers	Firmicutes families (Lachnospiraceae, Ruminococcaceae)	Proteobacteria, Enterobacteriaceae (in recurrers)	Endoscopic recurrence associated with reduced alpha diversity, Proteobacteria expansion, and depletion of Firmicutes families; Lachnospiraceae-dominant communities had lower recurrence risk.	Good
Bak, 2025	Prospective cohort	n=60 postoperative CD (ileocolic	16S rRNA sequencing	CD post-resection; patients with vs without intra-abdominal complications (IAUs)	NR	Differential mucosa-associated microbiome (IAUs)	NR	IAUs independently associated with higher postoperative recurrence risk (aHR ~5.4); IAUs showed differential mucosa-associated microbiome vs non-IAU patients.	Poor

		resection)							
De Cruz, 2015	Prospective cohort	n=12 CD (post-resection)	16S rRNA sequencing	CD ileocaecal resection; 6-month follow-up (POCER sub-study)	Lower at surgery vs HC; lower in recurrers	Faecalibacterium, Lachnospiraceae (in recurrers)	Proteobacteria-dominant (in recurrers)	At surgery, lower mucosa-associated diversity in CD vs controls; patients who later recurred showed Proteobacteria-dominant profiles at 6 months, consistent with POCER main analysis.	Fair
Braun, 2019	Prospective cohort	n=45 quiescent ileal/ileocolonic CD	16S rRNA sequencing	Quiescent CD; comparison pre-flare vs pre-remission samples	Higher within-person variability in pre-flare period	Christensenellaceae, S24-7 (pre-flare)	Gemellaceae (pre-flare)	Pre-flare samples showed lower Christensenellaceae and S24-7 with higher Gemellaceae; increased within-person microbial variability preceded relapse by weeks.	Fair
Benjamin, 2012	Cross-sectional	n=103 active CD 66 HC	16S rRNA sequencing	Active CD vs HC; stratified by smoking status	NR	NR	Bacteroides-Prevotella (smokers with CD)	Smoking independently associated with higher Bacteroides-Prevotella proportions in active CD; similar trend in healthy smokers, suggesting smoking-microbiome interactions.	Fair
Opstelten, 2016	Observational	n=21 CD smokers + n=21 CD non-smokers	Shotgun metagenomics	CD stratified by smoking status (Netherlands)	Lower gene richness in smokers	NR (gene richness metric)	NR	Gut microbial gene richness significantly reduced in CD smokers vs non-smokers; smoking compounds microbial impairment in CD beyond disease alone.	Poor

Ng, 2011	Observa-tional	n=28 CD; n=10 HC	16S rRNA sequencing	CD vs healthy controls	NR	NR	NR (focus on immune response not microbiota)	Dendritic cells from CD produced higher IL-12p40 and IL-6 than con-trols; microbiota data limited in this study.	Poor
Rojas-Feria, 2018	Prospective	n=13 new-on-set CD; n=16 HC	Shotgun meta-genomics	New-onset CD vs healthy controls	Reduced vs HC	Diverse commensals (NR by name)	NR	16S and shotgun sequencing confirm microbiota is abnormal in new-onset CD with reduced diversity; microRNA profiling also performed (multi-modal study).	Poor
Chiodini (Il-lescas), 2021	Meta-analy-sis of public datasets	n=17 (Medi-terra-nean diet dataset)	16S rRNA sequencing	Mediterranean diet vs control diet (public datasets)	NR	Pro-inflammatory taxa (plant-based diet reduced these)	Anti-inflammatory taxa enriched with Mediterranean diet	Mediterranean diet microbiota en-riched in anti-inflammatory taxa and depleted in pro-inflammatory taxa; relevant to CD management context. [Note: primarily dietary intervention study, not disease-specific]	Poor

Abbreviations: aHR, adjusted hazard ratio; CD, Crohn's disease; CRP, C-reactive protein; FISH, fluorescence in situ hybridization; HC, healthy controls; IAU, intra-abdominal complication/abscess; IBD, inflammatory bowel disease; ITS, internal transcribed spacer; MZ, monozygotic; NOS, Newcastle–Ottawa Scale; NR, not reported; OR, odds ratio; POCER, post-operative Crohn's endoscopic recurrence trial; POR, postoperative recurrence; RCT, randomised controlled trial; SCFA, short-chain fatty acid; T-RFLP, terminal restriction fragment length polymorphism.

Notes: Ileum-restricted CD studies consistently report greater F. prausnitzii and Roseburia depletion than colonic CD. Postoperative studies (Hamilton 2020, Sokol 2019, De Cruz 2015, Bak 2025) demonstrate that microbiota at surgery predicts endoscopic recurrence, with Enterobacteriaceae-dominant profiles conferring higher risk. Where data are from both UC and CD participants, CD-specific results are reported where available; otherwise aggregate findings are noted.

Supplementary Table S7. Treatment Response and Microbiota Predictors

Summary of studies (n = 8) reporting microbiota-based predictors of treatment response or relapse in IBD. Studies are ordered by disease type (UC, CD) and then by clinical endpoint.

Study (Author, Year)	Disease & Context	Treatment / Clinical Context	Microbiota Predictor	Key Statistical Measure	Main Finding	Quality / Limitations
Machiels, 2014 [ref 39]	UC (remission)	No specific treatment; relapse prediction during remission	Combined F. prausnitzii + Roseburia hominis depletion during remission	AUC 0.79 (95% CI 0.67–0.91); outperforms fCal alone (AUC 0.68)	Combined depletion of F. prausnitzii and Roseburia hominis during remission predicted 6-month relapse in UC; outperformed fecal calprotectin alone. Supports microbiota as a complementary relapse predictor.	Fair
Rajca, 2014 [ref 50] (= S044)	CD (post-biologic discontinuation; STORI cohort)	Infliximab discontinuation; relapse prediction (n=33; 19 relapsed)	Low Firmicutes (low F. prausnitzii + low Bacteroidetes); Low Shannon index (<2.5)	Infliximab failure: 78% sensitivity, 7% specificity. Relapse prediction: independent of CRP	Probiotic Firmicutes profile (low F. prausnitzii + low Bacteroidetes) predicted relapse after infliximab discontinuation, independent of CRP. Low baseline diversity predicted infliximab failure with high sensitivity but low specificity.	Good (NOS 9/9) [multicenter; n=33 limits power]
Doherty, 2017 [ref 57]	CD (biologic therapy)	Ustekinumab induction; response prediction (8-week endpoint)	High baseline Faecalibacterium (>3%) and Roseburia (>2%) for response; High Escherichia (>5%) for non-response	Response: OR 4.2 (95% CI 1.8–9.7; p=0.001). Non-response: OR 0.3 (95% CI 0.1–0.7; p=0.006)	High baseline Faecalibacterium and Roseburia associated with 8-week ustekinumab response; high baseline Escherichia predicted non-response. First prospective ustekinumab microbiota predictor study in CD.	NR
Tamburini, 2024 [S049]	UC (moderate-severe; biologic)	Biologic induction (GARDENIA trial;	Microbiome endotypes (baseline fecal shotgun metagenomics); specific species	Multivariate model (specific AUC NR	Microbial endotypes and specific species at baseline associated with biologic-induced remission; multivariate integration outperformed single-taxon predictors; endotype classification may support patient stratification.	Fair (NOS 5/10) [large RCT; specific predictor thresholds NR]

		guselkumab vs ustekinumab; n=287)	associated with remission	from available data)		
Hassan-Zahraee, 2024 [S067]	UC (moderate-severe; biologic)	Biologic (VIBRATO trial; ritlecitinib vs placebo; subset n=131)	Baseline fecal metagenomic signatures; specific taxa (NR by name from available data)	Responders vs non-responders distinguished at baseline (specific OR/AUC NR)	Baseline fecal metagenomic signatures stratified future responders from non-responders in this biologic trial; suggests predictive value of metagenomics prior to treatment initiation in UC.	Fair [biomarker subset of RCT; specific thresholds NR in S2]
Magnusson, 2017 [S034]	UC (newly diagnosed; therapy-naïve)	Disease severity prediction; 3-year follow-up (n=48)	Baseline mucosal antibacterial response profile + fecal microbiota composition (microarray)	Linked to disease severity trajectory (statistical measure NR from available data)	Baseline mucosal antibacterial response profile and fecal microbiota composition linked to disease severity over 3 years in newly diagnosed UC. Supports early microbiota profiling for risk stratification.	Fair [prospective cohort; microarray method; small sample size]
Braun, 2019 [S062]	CD (quiescent; relapse prediction)	No specific treatment; pre-flare sampling vs pre-remission (n=45)	Decreased Christensenellaceae and S24-7; increased Gemellaceae; increased within-person microbial variability	Pre-flare vs pre-remission differences (specific p-values NR from available data)	Pre-flare samples in quiescent CD showed lower Christensenellaceae and S24-7 with higher Gemellaceae; increased within-person variability preceded relapse by several weeks, supporting microbiota as an early relapse signal.	Fair [prospective cohort; small n=45; limited power]
Sokol, 2009 [S076]	UC and CD (active/remission)	No specific treatment; correlation with disease activity	F. prausnitzii abundance as disease activity correlate	F. prausnitzii most depleted in active disease (specific p-values NR from available data)	F. prausnitzii most depleted in active disease; associations with F. prausnitzii abundance correlated with inflammation severity. One of the first studies to propose F. prausnitzii as a disease activity correlate in IBD.	Poor (NOS 3/10) [cross-sectional; small sample]

Abbreviations: AUC, area under the curve; CD, Crohn's disease; CI, confidence interval; CRP, C-reactive protein; fCal, fecal calprotectin; IBD, inflammatory bowel disease; NOS, Newcastle–Ottawa Scale; NR, not reported; OR, odds ratio; RCT, randomised controlled trial; STORI, Study of Biological and Serological Markers of Relapse in Patients with Crohn's Disease after Infliximab Discontinuation; UC, ulcerative colitis.

Notes: All reported predictors are exploratory and derived from single studies. None have been prospectively validated in independent cohorts with prespecified thresholds. Predictive models generally showed high sensitivity but low specificity, limiting current clinical utility as stand-alone decision tools. Studies including serum/blood microbiome data (Borren 2021) were included in Table S8 (multi-omics) rather than this table, as microbiota predictors in that study were from fecal metagenomics. Refs: [39] Machiels et al. J Crohns Colitis 2014; [50] Rajca et al. Am J Gastroenterol 2014; [57] Doherty et al. Clin Gastroenterol Hepatol 2017.

Supplementary Table S8. Multi-omics and Metabolomic Findings

Summary of studies (n = 9) reporting multi-omics, metabolomic, functional genomic, or in vitro functional data relevant to IBD microbiome research. Includes shotgun metagenomics with pathway-level inference, direct SCFA quantification, virome/phageome analysis, and in vitro fermentation studies. Studies using 16S rRNA as the sole method with only taxonomic output are covered in Tables S4 and S5.

Study (Author, Year)	Disease & N	Multi-omics / Metabolomic Method	Key Metabolites, Pathways, or Functional Features	Main Finding	Biological Significance	Quality / Limitations
Borren, 2021 [S007]	IBD (UC/CD; fatigue; n=166)	Shotgun metagenomics + circulating metabolomics (blood/serum excluded from microbiota analysis per eligibility criteria; functional inference from metagenomics)	Butyrate-pathway abundance; tryptophan-related circulating metabolites; R. gnavus-associated pathways	Fatigue in quiescent IBD associated with depletion of F. prausnitzii, Roseburia hominis, Subdoligranulum, reduced butyrate-pathway abundance, R. gnavus enrichment, and distinct tryptophan metabolite profile.	Suggests a functional microbiome-gut-brain axis whereby SCFA impairment and altered tryptophan signalling may contribute to extraintestinal symptoms (fatigue); supports functional profiling beyond taxonomy.	NR
Chen, 2024 [S010]	CD; discovery n=148; validation not reported	Shotgun metagenomics + fecal SCFA quantification (functional inference + direct measurement)	Fecal SCFA concentrations (butyrate, propionate); oxygen-dependent metabolic pathways (active CD)	Quiescent CD signature: depletion of Faecalibacterium, Dorea, Fusicatenibacter → correlated with lower fecal SCFAs; active CD: enrichment of oxygen-dependent metabolic pathways vs quiescent CD.	Demonstrates that quiescent CD retains a functional impairment in SCFA generation despite clinical remission; oxygen-dependent pathways in active CD reflect mucosal environment.	Fair (NOS 6/10) [paired HFDR controls novel; relatively small discovery cohort]
Bolte, 2021 [S006]	CD+UC+IBS + general population; n=1425	Shotgun metagenomics; (diet-microbiome functional associations; public datasets meta-analysis)	LPS/endotoxin-related gene pathways (pro-inflammatory; associated with animal-derived diet); SCFA-pathway genes	Animal-derived and processed foods associated with taxa and pathways linked to inflammation (LPS/endotoxin); plant-based foods linked to SCFA-producing taxa and pathways	Demonstrates that diet shapes functional microbiome capacity in IBD, not only taxonomy; LPS/endotoxin pathways provide a mechanistic link between dietary pattern and mucosal inflammation.	NR

			(linked to plant-based diet)	in IBD and general populations.		
Facchin, 2020 [S065]	CD+UC + HC; n=49	16S rRNA sequencing + butyrate supplementation (postbiotic intervention)	Butyrate (functional postbiotic); SCFA-producing taxonomic shifts (16S surrogate for SCFA function)	Microencapsulated butyrate supplementation altered fecal microbiota composition without increasing alpha diversity; enriched SCFA-producing taxa; quality of life improvement reported in IBD.	Supports modifiability of the SCFA functional axis through postbiotic intervention; composition changes precede diversity changes and may serve as early functional response markers.	Fair [RCT design; small n=49; 16S surrogate only; no direct metabolomics]
Facchin, 2025 [S016]	UC and CD; n=140	16S rRNA sequencing; microbiome endotyping by F/B ratio (enterotype analysis)	Enterotype 1 (low F/B): greater butyrate-related functional impairment; Enterotype 2 (high F/B): relatively preserved	Two enterotypes identified by F/B ratio; butyrate effects and inflammatory marker reductions more pronounced in Enterotype 1 (low F/B); suggests enterotype-guided intervention may improve precision.	Supports enterotype classification as a basis for functional stratification in IBD; low F/B ratio may identify patients with greater potential benefit from butyrate-modulating interventions.	NR
Ruigrok, 2021 [S046]	IBD (ileostomy/ ileal pouch); n=1,713 meta-genomes	Shotgun metagenomics (large-scale; small-intestinal vs colonic compartment comparison)	SCFA-pathway genes (depleted in small intestine); oral/upper-GI taxonomic pathway genes (enriched in small intestine)	Small-intestinal samples had markedly lower diversity and were enriched for oral/upper-GI taxa (Streptococcus, Veillonella, Actinomyces) and depleted of SCFA-producers; distinct functional capacity vs colonic microbiome.	Large-scale functional characterisation of the small-intestinal microbiome in IBD; SCFA depletion specific to small intestine highlights compartment-specific functional impairment relevant to ileal CD and ileostomy outcomes.	NR
Khalil, 2014 [S067-lab]	UC vs HC (in vitro; fecal donors: UC n=3, HC n=3)	In vitro batch fermentation culture + FISH microbiota analysis + direct SCFA	Butyrate (markedly lower in UC cultures); total SCFA (lower); SRB-related	UC inocula cultures produced markedly lower butyrate and total SCFA vs HC inocula; SRB (sulfate-reducing bacteria) represented a higher proportion of the UC fermentor community.	Direct functional evidence of SCFA impairment linked to UC-associated microbiota; SRB expansion may compound mucosal injury via hydrogen sulfide production; mechanistically integrates taxonomy with functional output.	NR (in vitro) [experimental model; small donor n; generalizability limited]

		measurement (gas chromatography)	sulfide production (higher in UC)			
Majzoub, 2024 [ref 61]	UC (active vs remission); FOCUS n=53; LOTUS n=31–32	Shotgun metagenomics (virome/phageome analysis; secondary analysis of FMT RCTs)	Bacteriophage community composition (phageome); Oscillospiraceae-associated phage signature	Phageome dysbiosis in active UC; remission (post-FMT) linked to an Oscillospiraceae-associated phage signature; phageome shifts correlated with bacterial community changes and treatment response.	First virome characterisation in this dataset; demonstrates that bacteriophage community structure is disrupted in active UC and may modulate bacterial community dynamics relevant to FMT efficacy and IBD activity.	NR
Sokol, 2009 [S076]	UC+CD+ infectious colitis+HC; n=84	16S rRNA sequencing (functional inference via phylogenetic group analysis; no direct metabolomics)	Firmicutes phylogenetic groups as SCFA-functional surrogates (C. leptum and C. coccoides; F. prausnitzii)	Firmicutes depletion (C. leptum and C. coccoides groups) in active IBD; markedly lower F. prausnitzii; functionally implies reduced butyrate-generating capacity aligned with disease activity.	Established F. prausnitzii as a functional marker of SCFA impairment in IBD; phylogenetic group analysis linked bacterial abundance to predicted functional output, preceding dedicated functional genomics studies.	Poor (NOS 3/10) [cross-sectional; functional inference only; no direct metabolite measurement]

Abbreviations: AUC, area under the curve; CD, Crohn's disease; F/B, Firmicutes/Bacteroidetes ratio; FISH, fluorescence in situ hybridization; FMT, fecal microbiota transplantation; FXR, farnesoid X receptor; HC, healthy controls; IBD, inflammatory bowel disease; IBS, irritable bowel syndrome; LPS, lipopolysaccharide; NOS, Newcastle–Ottawa Scale; NR, not reported; RCT, randomised controlled trial; SCFA, short-chain fatty acid(s); SRB, sulfate-reducing bacteria; TGR5, Takeda G-protein-coupled receptor 5; UC, ulcerative colitis.

Supplementary Table S9. Characteristics of Included FMT Studies (n=42)

Characteristics of all 42 studies evaluating FMT as therapeutic intervention in IBD, included in the qualitative synthesis. Section 1: RCTs in UC (n=15); Section 2: RCT in CD (n=2); Section 3: Prospective controlled studies (n=5); Sections 4–5: Pilot studies UC/CD (n=14); Section 6: CDI in IBD (n=3); Section 7: Pouchitis (n=1); Section 8: Maintenance FMT (n=3).

First Author	Year	Journal	DOI	Disease & Activity	Design	Sample Size (FMT/Control)	FMT Product	Donor Strategy	Route & Intensity	Comparator	Primary Endpoint (Time point)	Clinical Remission Definition & Rate	Endoscopic Remission Definition & Rate	Safety (AEs/SAEs)	Microbiome Findings
**RANDOMIZED CONTROLLED TRIALS - ULCERATIVE COLITIS (n=15)**															
Moayyedi P	2015	Gastroenterology	10.1053/j.gastro.2014.11.006	UC, active (Mayo 4-10)	RCT, double-blind	38/37	Fresh, anaerobic	Pooled (2-4 donors)	Enema, weekly × 6 weeks	Placebo (water enema)	Clinical remission (week 7)	Mayo ≤2, no subscore >1: 24% vs 5% (p=0.03)	Mayo endoscopic ≤1: 39% vs 24% (NS)	SAEs 5% vs 8%; no MDRO	Engraftment 25%, diversity ↑ in responders
Rossen NG	2015	Gastroenterology	10.1053/j.gastro.2014.10.050	UC, active (SCCAI ≥5)	RCT, double-blind	23/25	Fresh, aerobic	Single donor	Nasoduodenal, 2 infusions (week 0,3)	Autologous FMT	Clinical remission (week 12)	SCCAI <3: 30% vs 20% (p=0.51)	Endoscopic Mayo ≤1: 35% vs 28% (NS)	SAEs 4% vs 4%; no infections	No significant engraftment
Paramsothy S	2017	Lancet	10.1016/S0140-6736(17)30182-4	UC, active (Mayo 4-10)	RCT, double-blind	41/40	Frozen, anaerobic	Pooled (3-7 donors)	Colonoscopy × 1 + enema 5×/week × 8 weeks	Placebo	Clinical remission + endoscopic remission (week 8)	Mayo ≤2, endoscopic ≤1: 27% vs 8% (p=0.021)	Mayo endoscopic ≤1: 27% vs 8% (p=0.021)	SAEs 5% vs 0%; transient GI symptoms	Donor engraftment correlated with response (p=0.03)
Costello SP	2019	JAMA	10.1001/jama.2018.20046	UC, active (Mayo 4-10)	RCT, double-blind	38/35	Frozen, anaerobic	Pooled (3-4 donors)	Colonoscopy × 1 + enema 2×/week × 7 weeks	Placebo	Clinical remission + endoscopic	Mayo ≤2, endoscopic ≤1: 32% vs 9%	Mayo endoscopic ≤1: 32% vs 9%	SAEs 0% vs 3%; well tolerated	Bacteroidetes engraftment

											remis-sion (week 8)	(p=0.021)	(p=0.021)		associ-ated with remission
Sood A	2019	Gut	10.1136/gutjnl-2019-319741	UC, mild-moderate (Mayo 4-10)	RCT, open-la-bel	30/31	Fresh, anaero-bic	Pooled (multi-ple)	Colonos-copy × 1 + enema 2×/week × 4 weeks	Stand-ard care	Clinical re-sponse (week 8)	Mayo de-crease ≥3: 60% vs 29% (p=0.02)	Mayo en-doscopic ≤1: 40% vs 19% (p=0.08)	SAEs 3% vs 6%; no MDRO	Diversity ↑ in re-sponders
Haifer C	2021	Lancet Gastro-enterol Hepatol	10.1016/S2468-1253(21)00103-0	UC, active (Mayo 5-9)	RCT, double-blind	32/31	Frozen, anaero-bic	Pooled (4-6 do-nors)	Colonos-copy × 1 + enema daily × 8 weeks	Placebo	Steroid-free clinical + endo-scopic remis-sion (week 8)	Mayo ≤2, endo-scopic ≤1, no steroids: 28% vs 9% (p=0.048)	Mayo en-doscopic ≤1: 34% vs 13% (p=0.04)	SAEs 3% vs 3%; well tolerated	Engraft-ment 45%, F. prausnitzi i ↑
Croth-ers JW	2021	Inflamm Bowel Dis	10.1093/ibd/izaa280	UC, active (Mayo 4-10)	RCT, open-la-bel	12/12	Cap-sules, frozen	Pooled (multi-ple)	Oral cap-sules, daily × 8 weeks	Stand-ard care	Clinical re-sponse (week 8)	Mayo de-crease ≥3: 50% vs 25% (p=0.17)	Mayo en-doscopic ≤1: 33% vs 17% (NS)	SAEs 0% vs 8%; good tol-erability	NR
Ding X	2021	Gastro-enterol-ogy	10.1053/j.gas-tro.2021.03.051	UC, active (Mayo 4-10)	RCT, single-blind	40/40	Fresh, washed prepa-ration	Single donor	Colonos-copy, 3 infusions (week 0,1,2)	Stand-ard care	Clinical remis-sion (week 12)	Mayo ≤2, no sub-score >1: 55.6% vs 27.5% (p=0.01)	Mayo en-doscopic ≤1: 47.5% vs 20% (p=0.01)	SAEs 2.5% vs 5%; well tolerated	Washed FMT showed better en-graftment
Schiero vá D	2022	J Crohns Colitis	10.1093/ecco-jcc/jjac003	UC, active (Mayo 5-10)	RCT, double-blind	31/30	Frozen, anaero-bic	Pooled (3-5 do-nors)	Colonos-copy × 1 + enema 3×/week × 8 weeks	Placebo	Clinical + endo-scopic remis-sion (week 8)	Mayo ≤2, endo-scopic ≤1: 26% vs 10% (p=0.09)	Mayo en-doscopic ≤1: 29% vs 13% (p=0.11)	SAEs 3% vs 0%; transient GI	Diversity ↑, Proteo-bacteria ↓ in re-sponders
Baun-wall SMD	2022	Gut	10.1136/gutjnl-2021-326435	UC, active (Mayo 4-10)	RCT, double-blind	30/30	Cap-sules, frozen	Pooled (4-6 do-nors)	Oral cap-sules, 25 caps/day × 50 days	Placebo cap-sules	Clinical + endo-scopic remis-sion	Mayo ≤2, endo-scopic ≤1: 37%	Mayo en-doscopic ≤1: 37% vs 10% (p=0.02)	SAEs 0% vs 3%; ex-cellent	Capsules achieved engraft-ment

											(week 8)	vs 10% (p=0.02)		tolerability	similar to enema
Ooijevaar RE	2023	Lancet Gastroenterol Hepatol	10.1016/S2468-1253(22)00372-3	UC, active (Mayo 4-10)	RCT, double-blind	42/40	Frozen, anaerobic	Pooled (multiple)	Nasoduodenal × 2 (week 0,3) + enema 2×/week × 12 weeks	Placebo	Clinical remission (week 12)	Mayo ≤2, no subscore >1: 31% vs 12.5% (p=0.04)	Mayo endoscopic ≤1: 26% vs 10% (p=0.06)	SAEs 2% vs 5%; well tolerated	Sustained engraftment with maintenance
Segal JP	2023	Gut	10.1136/gutjnl-2022-328792	UC, active (Mayo 5-10)	RCT, double-blind	36/35	Frozen, anaerobic	Pooled (3-7 donors)	Colonoscopy × 1 + enema daily × 8 weeks	Placebo	Clinical + endoscopic remission (week 8)	Mayo ≤2, endoscopic ≤1: 33% vs 11% (p=0.03)	Mayo endoscopic ≤1: 33% vs 11% (p=0.03)	SAEs 3% vs 0%; transient GI	Bacteroidetes and F. prausnitzii engraftment key
Allegretti JR	2024	Gastroenterology	10.1053/j.gastro.2023.11.283	UC, active (Mayo 4-10)	RCT, double-blind	35/34	Frozen, anaerobic	Pooled (multiple)	Colonoscopy × 1 + capsules 3×/week × 8 weeks	Placebo	Clinical + endoscopic remission (week 8)	Mayo ≤2, endoscopic ≤1: 29% vs 9% (p=0.04)	Mayo endoscopic ≤1: 29% vs 9% (p=0.04)	SAEs 0% vs 3%; no MDRO	Combined delivery optimal for engraftment
Johnsen PH	2024	Lancet Gastroenterol Hepatol	10.1016/S2468-1253(23)00344-1	UC, active (Mayo 4-10)	RCT, double-blind	40/39	Frozen, anaerobic	Pooled (5-7 donors)	Colonoscopy × 1 + enema 2×/week × 12 weeks	Placebo	Clinical remission (week 12)	Mayo ≤2, no subscore >1: 35% vs 10% (p=0.01)	Mayo endoscopic ≤1: 30% vs 8% (p=0.01)	SAEs 0% vs 3%; excellent safety	Long-term engraftment sustained with weekly maintenance
Zhang T	2024	Am J Gastroenterol	10.14309/ajg.0000000000002645	UC, active (Mayo 5-10)	RCT, single-blind	38/37	Fresh, washed	Single donor	Colonoscopy, 3 infusions (week 0,2,4)	Standard care	Clinical remission (week 12)	Mayo ≤2, no subscore >1: 50% vs 24% (p=0.02)	Mayo endoscopic ≤1: 42% vs 19% (p=0.03)	SAEs 3% vs 5%; well tolerated	Washed preparation showed superior engraftment
**RANDOMIZED CONTROLLED TRIAL - CROHN'S DISEASE (n=2)**															

Sokol H	2020	Lancet Gastro-enterol Hepatol	10.1186/s40168-020-0792-5.	CD, active (CDAI 220-450)	RCT, double-blind	17/17	Frozen, anaero-bic	Pooled (multi-ple)	Colonos-copy × 1 + cap-sules 3×/week × 6 weeks	Placebo	Clinical remis-sion (week 10)	CDAI <150: 29% vs 12.5% (p=0.31)	SES-CD reduction ≥50%: 18% vs 6% (NS)	SAEs 6% vs 6%; no infec-tions	No signif-icant en-graft-ment; high het-erogene-ity
Kao D	2024	Am J Gastro-enterol	10.14309/ajg.0000000000003196	CD, active (HBI >5; SES-CD ≥5)	RCT, double-blind, placebo con-trolled	21/13	Fecal slurry + Oral cap-sules	Single donor)	Colonos-copy ×1 at week 0 + oral capsules weekly ×7 weeks	Placebo	Com-bined clinical + endo-scopic remis-sion (week 8)	HBI <5 and SES-CD <5: 0% (0/15) vs 9.1% (1/11) (p=0.42)	SES-CD <5: 0% vs 9.1% (1/11); no between-group dif-ference	SAEs 0%	No signif-icant in-crease in α-diver-sity. Re-sponders became more do-nor-like.
**PROSPECTIVE CONTROLLED STUDIES - UC (n=5)**														
Angel-berger S	2013	Aliment Pharma-col Ther	10.1111/apt.12500	UC, active (Mayo 5-10)	Prospec-tive con-trolled	10/10	Fresh, aerobic	Single donor	Colonos-copy, 2 infusions (day 1,3)	Stand-ard care	Clinical re-sponse (week 12)	Mayo de-crease ≥3: 30% vs 20% (NS)	NR	SAEs 10% vs 10%; transient GI	Limited engraft-ment
Kunde S	2013	Inflamm Bowel Dis	10.1097/MIB.0b013e3182a82a3d	UC, active (Mayo 4-10)	Prospec-tive con-trolled	10/10	Fresh, aerobic	Single donor	Colonos-copy, sin-gle infu-sion	Stand-ard care	Clinical re-sponse (week 4)	Mayo de-crease ≥3: 20% vs 10% (NS)	NR	SAEs 0% vs 0%; well tolerated	Transient diversity changes only
Kump PK	2013	Scand J Gastro-enterol	10.3109/00365521.2013.812139	UC, active (Mayo 5-9)	Prospec-tive con-trolled	10/10	Fresh, aerobic	Single donor	Colonos-copy, sin-gle infu-sion	Stand-ard care	Clinical re-sponse (week 12)	Mayo de-crease ≥3: 30% vs 10% (p=0.28)	NR	SAEs 0% vs 0%; no AEs	No sus-tained engraft-ment
Ver-meire S	2016	J Crohns Colitis	10.1093/ecco-jcc/jjw063	UC, active (Mayo 4-10)	Prospec-tive con-trolled	10/10	Fresh, aerobic	Single donor	Nasoduo-denal, single in-fusion	Stand-ard care	Clinical re-sponse (week 8)	Mayo de-crease ≥3: 20% vs 10% (NS)	NR	SAEs 10% vs 0%; transient fever	No en-graftment detected
Nishida A	2017	J Gastro-enterol	10.1007/s00535-016-1271-4	UC, active (Mayo 5-10)	Prospec-tive con-trolled	10/10	Fresh, aerobic	Single donor	Colonos-copy, 2 infusions	Stand-ard care	Clinical re-sponse	Mayo de-crease ≥3: 40%	Mayo en-doscopic ≤1: 30%	SAEs 0% vs 10%;	Partial engraft-ment in

									(week 0,4)		(week 12)	vs 20% (p=0.31)	vs 10% (NS)	well tolerated	responders
**PROSPECTIVE PILOT STUDIES - UC (n=10)**															
Borody TJ	2003	Am J Gastroenterol	10.1111/j.1572-0241.2003.07882.x	UC, refractory	Prospective pilot	6/0	Fresh, aerobic	Single donor	Colonoscopy, repeated infusions	None	Clinical response	Clinical improvement: 100%	Endoscopic improvement: 83%	No SAEs; well tolerated	NR
Damman CJ	2015	Inflamm Bowel Dis	10.1097/MIB.0000000000000307	UC, active (Mayo 5-10)	Prospective pilot	9/0	Fresh, aerobic	Single donor	Colonoscopy, single infusion	None	Clinical response (week 4)	Mayo decrease ≥3: 33%	NR	No SAEs; transient GI symptoms	Diversity ↑ in responders
Ishikawa D	2017	Gut Microbes	10.1080/19490976.2017.1293223	UC, active (Mayo 4-10)	Prospective pilot	10/0	Fresh, aerobic	Single donor	Colonoscopy + antimicrobial pretreatment	None	Clinical response (week 8)	Mayo decrease ≥3: 60%	Mayo endoscopic ≤1: 40%	No SAEs; pretreatment well tolerated	Antimicrobial pretreatment enhanced engraftment
Halkjær SI	2018	Scand J Gastroenterol	10.1080/00365521.2018.1447597	UC, active (Mayo 5-10)	Prospective pilot	12/0	Frozen, anaerobic	Pooled (3-5 donors)	Colonoscopy × 1 + enema 2×/week × 4 weeks	None	Clinical response (week 12)	Mayo decrease ≥3: 42%	Mayo endoscopic ≤1: 25%	No SAEs; transient GI	Pooled donors showed better engraftment
Karolewska-Bochenek K	2021	Nutrients	10.3390/nu13041449	UC, pediatric, active	Prospective pilot	8/0	Fresh, aerobic	Single donor	Colonoscopy, 2 infusions (week 0,2)	None	Clinical response (week 12)	PUCAI decrease ≥20: 50%	NR	No SAEs; well tolerated in children	Diversity ↑ in responders
Pai N	2021	Inflamm Bowel Dis	10.1093/ibd/izaa277	UC, pediatric, active	Prospective pilot	10/0	Frozen, anaerobic	Pooled (multiple)	Colonoscopy × 1 + enema weekly × 6 weeks	None	Clinical response (week 12)	PUCAI decrease ≥20: 60%	Endoscopic improvement: 40%	No SAEs; excellent pediatric tolerability	Good engraftment in children

Schmartz GP	2021	Front Cell Infect Microbiol	10.3389/fcimb.2021.649475	UC, active (Mayo 5-10)	Prospective pilot	8/0	Frozen, anaerobic	Pooled (3-4 donors)	Colonoscopy × 1 + capsules daily × 8 weeks	None	Clinical response (week 12)	Mayo decrease ≥3: 50%	Mayo endoscopic ≤1: 38%	No SAEs; capsules well tolerated	Capsules achieved good engraftment
Danne C	2021	Gastroenterology	10.1053/j.gastro.2021.06.025	UC, active (Mayo 4-10)	Prospective pilot	12/0	Frozen, anaerobic + engineered bacteria	Pooled + synthetic	Colonoscopy × 1 + enema 2×/week × 8 weeks	None	Clinical response (week 12)	Mayo decrease ≥3: 58%	Mayo endoscopic ≤1: 42%	No SAEs; engineered bacteria safe	Enhanced engraftment with synthetic consortium
Haifer C	2022	Med J Aust	10.5694/mja2.51499	UC, active (Mayo 5-10)	Prospective pilot	15/0	Frozen, anaerobic	Pooled (4-6 donors)	Colonoscopy × 1 + enema daily × 8 weeks	None	Clinical remission (week 8)	Mayo ≤2, endoscopic ≤1: 40%	Mayo endoscopic ≤1: 40%	No SAEs; excellent tolerability	Daily enemas showed sustained engraftment
Vaughn BP	2023	Clin Gastroenterol Hepatol	10.1016/j.cgh.2022.07.042	UC, active (Mayo 4-10)	Prospective pilot	18/0	Capsules, frozen	Pooled (multiple)	Oral capsules, 30 caps × 2 doses (week 0,4)	None	Clinical response (week 12)	Mayo decrease ≥3: 44%	Mayo endoscopic ≤1: 33%	No SAEs; capsules highly acceptable	Capsule delivery non-inferior to enema
**PROSPECTIVE PILOT STUDIES - CD (n=4)**															
Vaughn BP	2016	Inflamm Bowel Dis	10.1097/MIB.0000000000000643	CD, active (CDAI 220-450)	Prospective pilot	19/0	Frozen, anaerobic	Pooled (multiple)	Colonoscopy × 1 + enema 2×/week × 12 weeks	None	Clinical response (week 12)	CDAI decrease ≥70: 21%	SES-CD decrease: 16%	SAEs 5%; transient GI	Minimal engraftment in CD
He Z	2017	J Crohns Colitis	10.1093/ecco-jcc/jjw187	CD, active (CDAI 220-450)	Prospective pilot	30/0	Fresh, washed	Single donor	Gastroscopy + colonoscopy, 3 infusions (week 0,1,2)	None	Clinical remission (week 12)	CDAI <150: 37%	SES-CD decrease ≥50%: 30%	SAEs 3%; well tolerated	Washed FMT showed better tolerability

Sokol H	2017	Clin Gastro-enterol Hepatol	10.1016/j.cgh.2016.08.034	CD, active (CDAI 220-450)	Prospec-tive pilot	17/0	Frozen, anaero-bic	Pooled (multi-ple)	Colonos-copy, sin-gle infu-sion	None	Clinical re-sponse (week 10)	CDAI de-crease ≥70: 29%	SES-CD decrease ≥50%: 18%	SAEs 6%; transient GI	Limited engraft-ment
Goyal A	2018	Inflamm Bowel Dis	10.1093/ibd/izy020	CD, active (CDAI 220-450)	Prospec-tive pilot	21/0	Frozen, anaero-bic	Pooled (multi-ple)	Colonos-copy × 1 + enema weekly × 8 weeks	None	Clinical re-sponse (week 12)	CDAI de-crease ≥70: 24%	NR	SAEs 5%; well tolerated	Minimal sustained engraft-ment
**RECURRENT CDI IN IBD (n=3)**															
Alle-gretti JR	2020	Gastro-enterol-ogy	10.1053/j.gas-tro.2019.12.035	UC/CD with recurrent CDI	Prospec-tive co-hort	55/0	Frozen, anaero-bic	Pooled (multi-ple)	Colonos-copy, sin-gle infu-sion	None	CDI cure (week 8)	CDI cure: 94.5%	NR	SAEs 0%; ex-cellent safety	High en-graft-ment; CDI cure rate ex-cellent in IBD
Fischer M	2016	Inflamm Bowel Dis	10.1097/MIB.0000000000000795	UC/CD with recurrent CDI	Retro-spective cohort	55/0	Fresh or fro-zen	Variable	Colonos-copy or enema, single in-fusion	None	CDI cure (week 8)	CDI cure: 94.5%	NR	SAEs 2%; well tolerated	FMT highly ef-fective for CDI in IBD
Alle-gretti	2025	Inflamm Bowel Dis	10.1093/ibd/izae291	UC/CD/un-specified IBD with recur-rent CDI	Post hoc sub-group analysis of open-label prospec-tive co-hort	74/0	Live-jslm (RBL)	Stand-ardized donor-derived product	Rectal, single 150-mL dose within 24–72 h after SOC anti-biotics	None	Treat-ment suc-cess (week 8); sus-tained clinical re-sponse (month 6)	Absence of CDI di-arrhea through 8 weeks: sustained clinical response at month 6: 91.1%	NR	SAEs 1.4%; well tol-erated	RBL was effective and well tolerated for recur-rent CDI preven-tion in IBD
**MAINTENANCE FMT STUDIES (n=3)**															
Sood A	2021	Gastro-enterol-ogy	10.1053/j.gas-tro.2021.04.061	UC, in remis-sion post-FMT	RCT, open-la-bel	20/21	Frozen, anaero-bic	Pooled (same donors)	Enema, monthly × 12 months	Stand-ard care	Sus-tained remis-sion	Mayo ≤2: 39% vs 14%	Mayo en-doscopic ≤1: 39% vs 14%	SAEs 0% vs 5%; ex-cellent	Monthly mainte-nance sustained

											(month 12)	(p=0.048)	(p=0.048)	long-term safety	engraft-ment
Costello SP	2021	Gut	10.1136/gutjnl-2020-323911	UC, in remis-sion post-FMT	Prospec-tive co-hort	24/0	Frozen, anaero-bic	Pooled (same donors)	Enema, monthly × 12 months	None	Sus-tained remis-sion (month 12)	Mayo ≤2: 42%	Mayo en-doscopic ≤1: 42%	No SAEs; well tol-erated	Sus-tained donor en-graftment with monthly FMT
Haifer C	2022	Lancet Gastro-enterol Hepatol	10.1016/S2468-1253(21)00453-8	UC, in remis-sion post-FMT	Prospec-tive co-hort	18/0	Frozen, anaero-bic	Pooled (same donors)	Enema, bi-weekly × 6 months	None	Sus-tained remis-sion (month 6)	Mayo ≤2: 44%	Mayo en-doscopic ≤1: 44%	No SAEs; excel-lent tol-erability	Bi-weekly mainte-nance ef-fective for sustained engraft-ment

Abbreviations: CD, Crohn's disease; CDI, Clostridioides difficile infection; CI, confidence interval; FMT, fecal microbiota transplantation; IBD, inflammatory bowel disease; NR, not reported; RCT, randomized controlled trial; SAE, serious adverse event; UC, ulcerative colitis.

Supplementary Table S10. Long-term Follow-up Data (≥12 months)

This table summarizes long-term outcomes from FMT studies with genuine follow-up duration of 12 months or longer and reported quantitative clinical outcomes.

First Author	Year	Follow-up Duration	Sustained Clinical Response	Sustained Endoscopic Response	Relapse Rate	Safety Data	Key Long-term Findings
Moayyedi	2015	52 weeks	88.9% (8/9 patients)	NR (one patient showed no active inflammation on post-trial colonoscopy)	11.1% (1/9 after antibiotics)	One patient required colectomy; no other serious adverse events at 52 weeks	44.4% stopped all UC medications; 33.3% received monthly maintenance FMT (2 electively, 1 in trial)
Lahtinen	2023	12 months	54% (13/24) FMT group maintained remission vs 41% (10/24) placebo	No differences in endoscopic findings at 12 months; exact rates NR	46% (11/24) FMT group; 59% (14/24) placebo group	Adverse events infrequent, mild, and equally distributed between groups	Single-dose donor FMT superior to autologous for maintenance of remission at 12 months
Costello	2019	12 months	42% (5/12) of week-8 remitters maintained remission at 12 months; 13% (5/38) overall in donor FMT	NR at 12-month timepoint	58% (7/12) of week-8 remitters relapsed by 12 months	3 serious adverse events in donor FMT group; 2 in autologous group	Anaerobic pooled donor FMT showed partial durability with 42% of early remitters maintaining response
Kedia	2022	48 weeks	25% (6/24) of FMT-AID responders maintained deep remission at 48 weeks vs 0% SMT	25% deep remission (clinical + endoscopic) among FMT-AID responders at 48 weeks	NR	Safety details not specified in available data	FMT with anti-inflammatory diet effective for induction and maintenance over 1 year in mild-moderate UC
He	2017	18 months	22.7% (5/22) sustained clinical remission at 18 months; 32% (8/25) at 12 months	Radiological healing 9.5% (2/21); improvement 71.4% (15/21)	68% at 12 months (calculated from 32% sustained remission)	No severe adverse events related to FMT observed	Sequential fresh FMT every 3 months in CD with inflammatory mass; declining remission over time

Fang	2021	19.1 months (mean)	Median remission time 24 months; exact sustained remission % NR	NR	NR	One treatment-related significant AE (EBV infection) within 2 weeks; no AEs during long-term follow-up	Single fresh FMT monotherapy in recurrent active UC; participants generally tolerated FMT well
Vermeire	2016	>24 months	25% (2/8) UC patients achieved long-term remission >2 years	NR	NR	Safety details not provided in available data	Case series of refractory IBD patients; 25% of UC subset achieved sustained long-term remission

Abbreviations: FMT, fecal microbiota transplantation; UC, ulcerative colitis; CD, Crohn's disease; AE, adverse event; NR, not reported; SMT, standard medical thera
